# Supplementary material for: Quantitative Analysis of Differential Expression of HOX Genes in Multiple Cancers
Source: Cancers (Basel). 2020 Jun 14;12(6):1572. doi: 10.3390/cancers12061572 (PMC7352544; doi:10.3390/cancers12061572)
Supplement: Supplementary file 1 [file cancers-12-01572-s001.zip › cancers-832750-supp-final-update.pdf]

Supplementary Materials: Quantitative Analysis of Differential Expression of HOX Genes in Multiple Cancers

Orit Adato, Yaron Orenstein, Juri Kopolovic, Tamar Juven-Gershon and Ron Unger

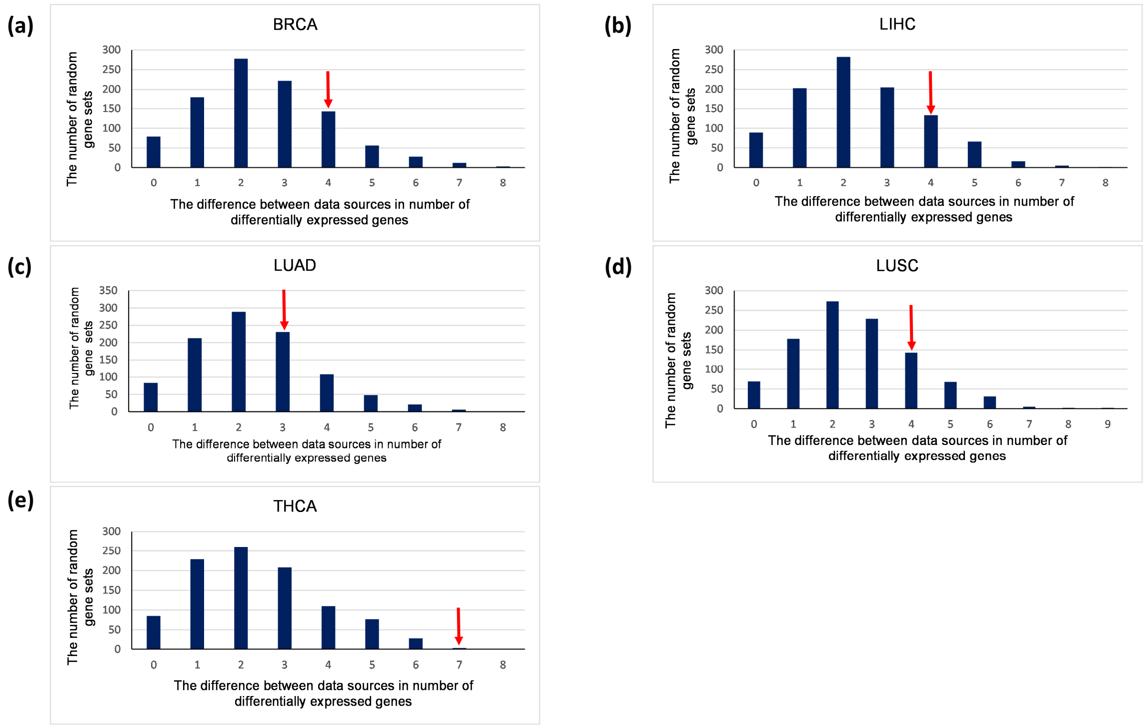

Figure S1. Differences in the number of differentially expressed genes between tumor and healthy cancer tissue samples from TCGA:TCGA as compared to TCGA:GTEx for *HOX* genes and random sets of genes for five cancer types for which TCGA contains at least 50 healthy samples. The *HOX* genes (marked by red arrows) are within the range of differences found for 1000 random sets of 39 genes. (a) Breast cancer (BRCA). (b) Liver hepatocellular carcinoma (LIHC). (c) Lung adenocarcinoma (LUAD). (d) Lung squamous cell carcinoma (LUSC). (e) Thyroid carcinoma (THCA). Note that only in THCA there is a significant difference in the behavior of *HOX* genes compared to random genes, which can be explained by the heterogeneity of Thyroid carcinoma (see text).

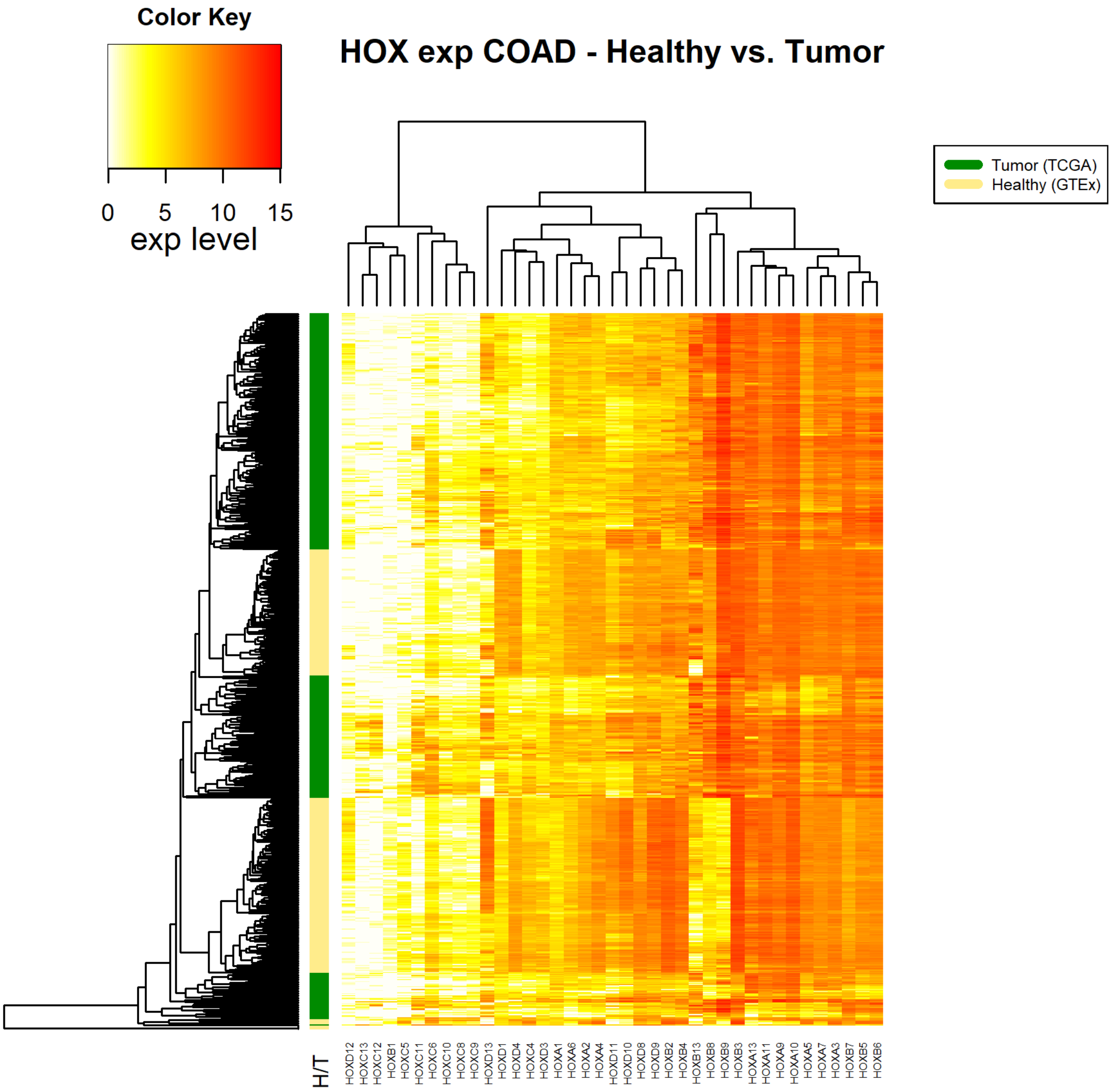

Figure S2. *HOX* gene expression in samples of healthy colon tissue and colon adenocarcinoma (COAD) tumor tissue. The source of expression data of healthy and tumor samples is GTEx and TCGA, respectively. H/T stands for healthy/tumor.

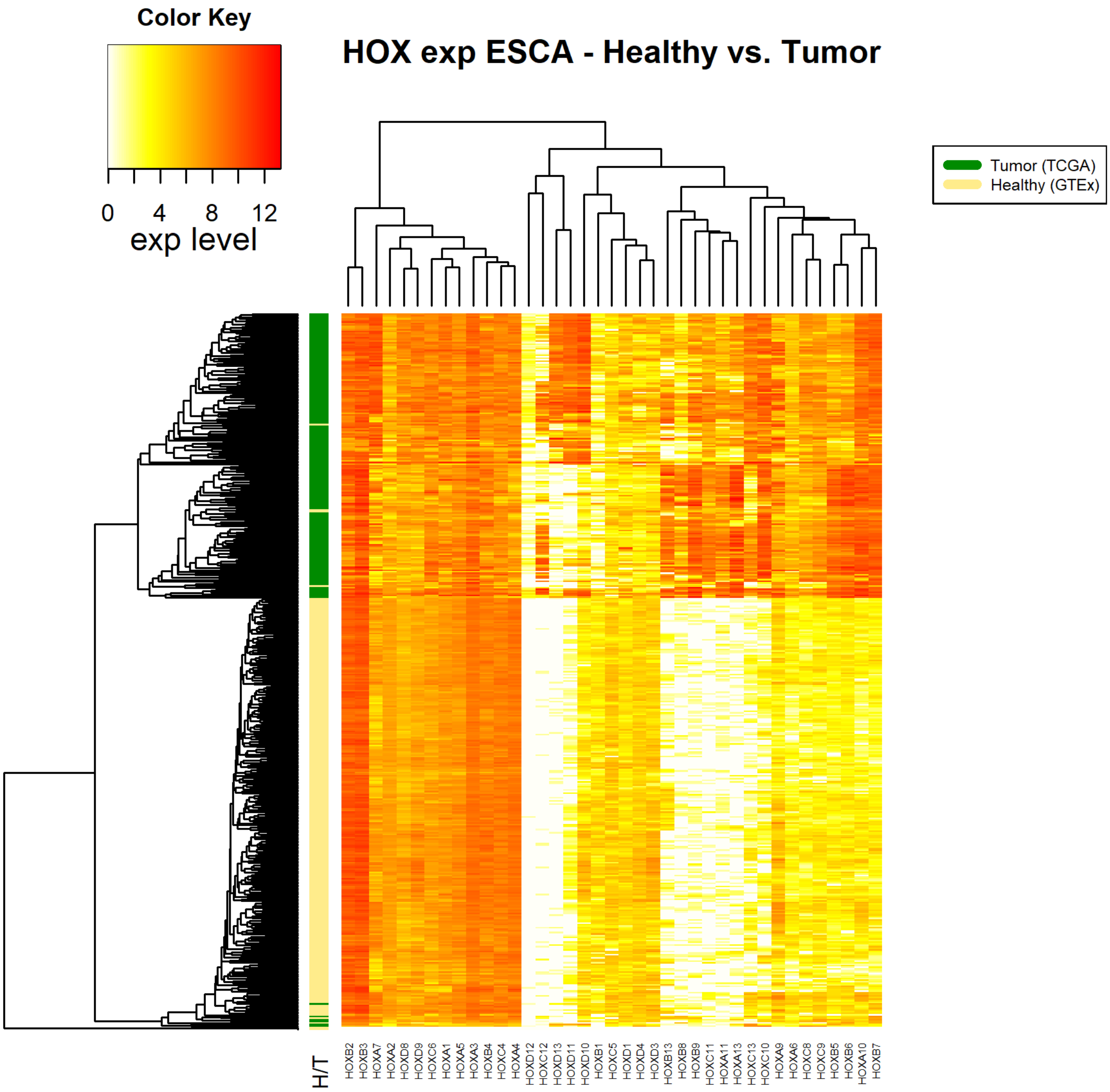

Figure S3. *HOX* gene expression in samples of healthy esophagus and esophageal carcinoma (ESCA) tumor tissue. The source of expression data of healthy and tumor samples is GTEx and TCGA, respectively. H/T stands for healthy/tumor.

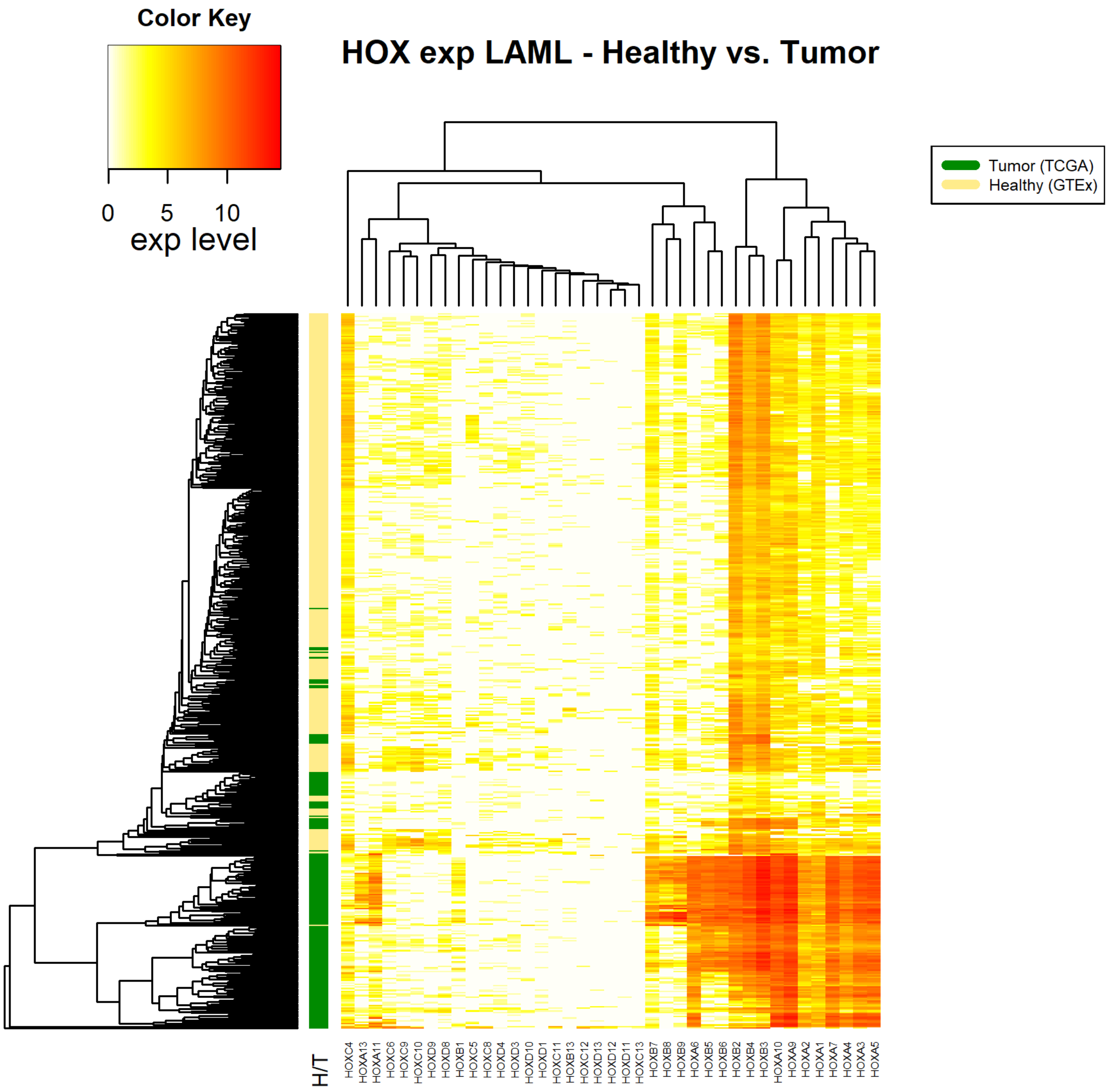

FigureS4. *HOX* gene expression in samples of healthy blood tissue and acute meloid leukemia (LAML) tumor tissue. The source of expression data of healthy and tumor samples is GTEx and TCGA, respectively. H/T stands for healthy/tumor.

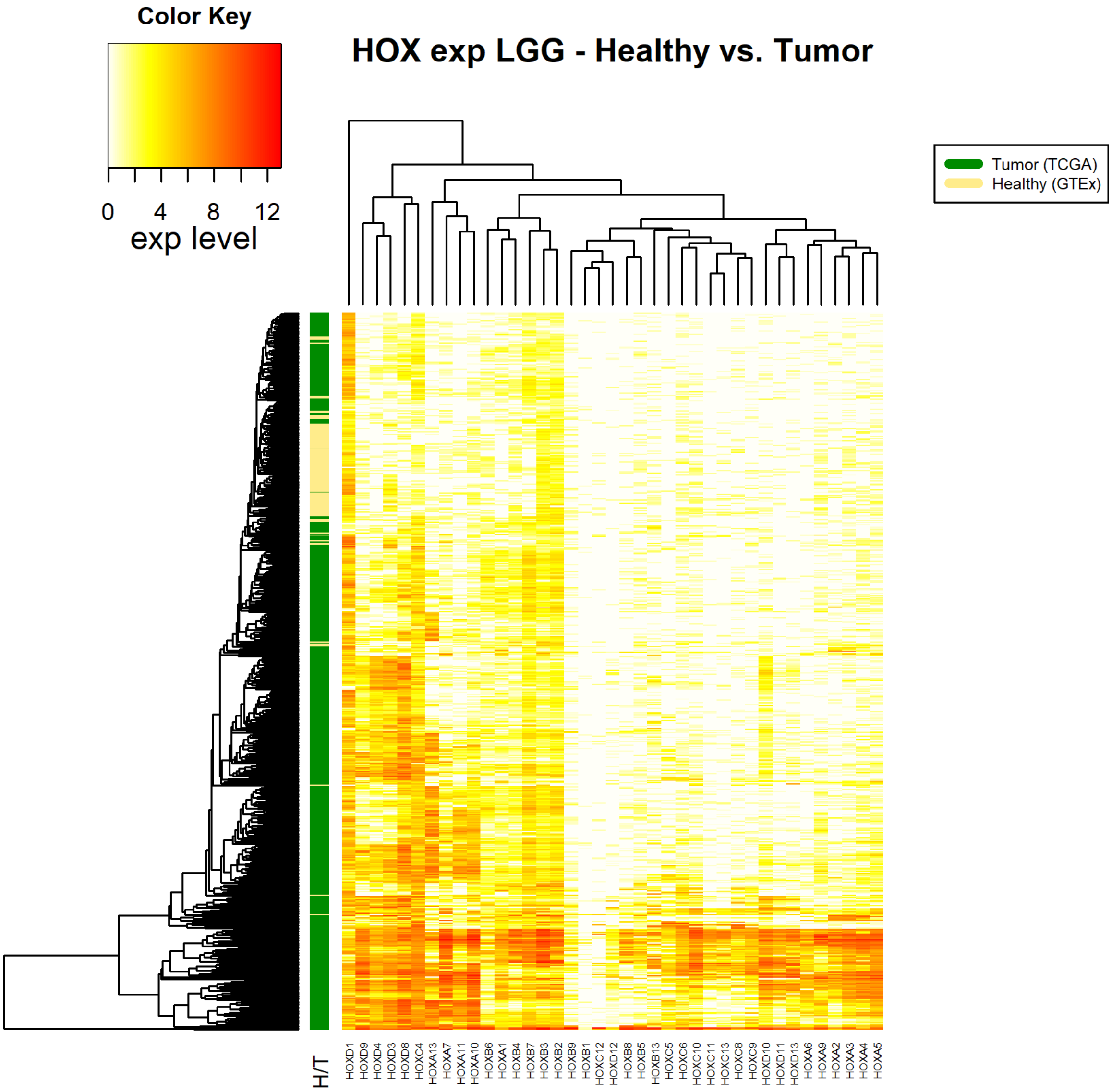

Figure S5. *HOX* gene expression in samples of healthy brain cortex tissue and tumor lower grade glioma (LGG) tissue. The source of expression data of healthy and tumor samples is GTEx and TCGA, respectively. H/T stands for healthy/tumor.

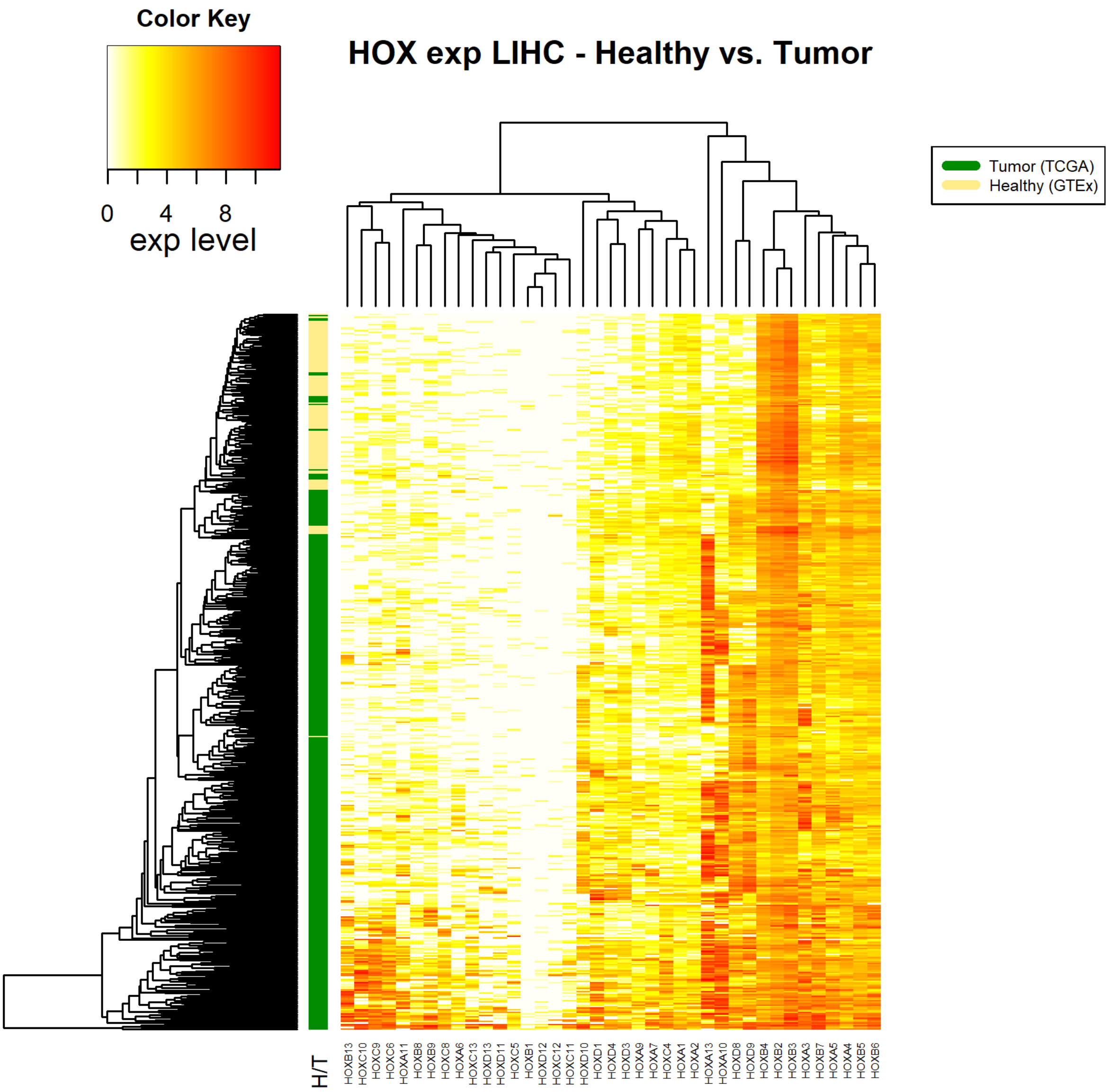

Figure S6. *HOX* gene expression in samples of healthy liver and liver hepatocellular carcinoma (LIHC) tumor tissue. The source of expression data of healthy and tumor samples is GTEx and TCGA, respectively. H/T stands for healthy/tumor.

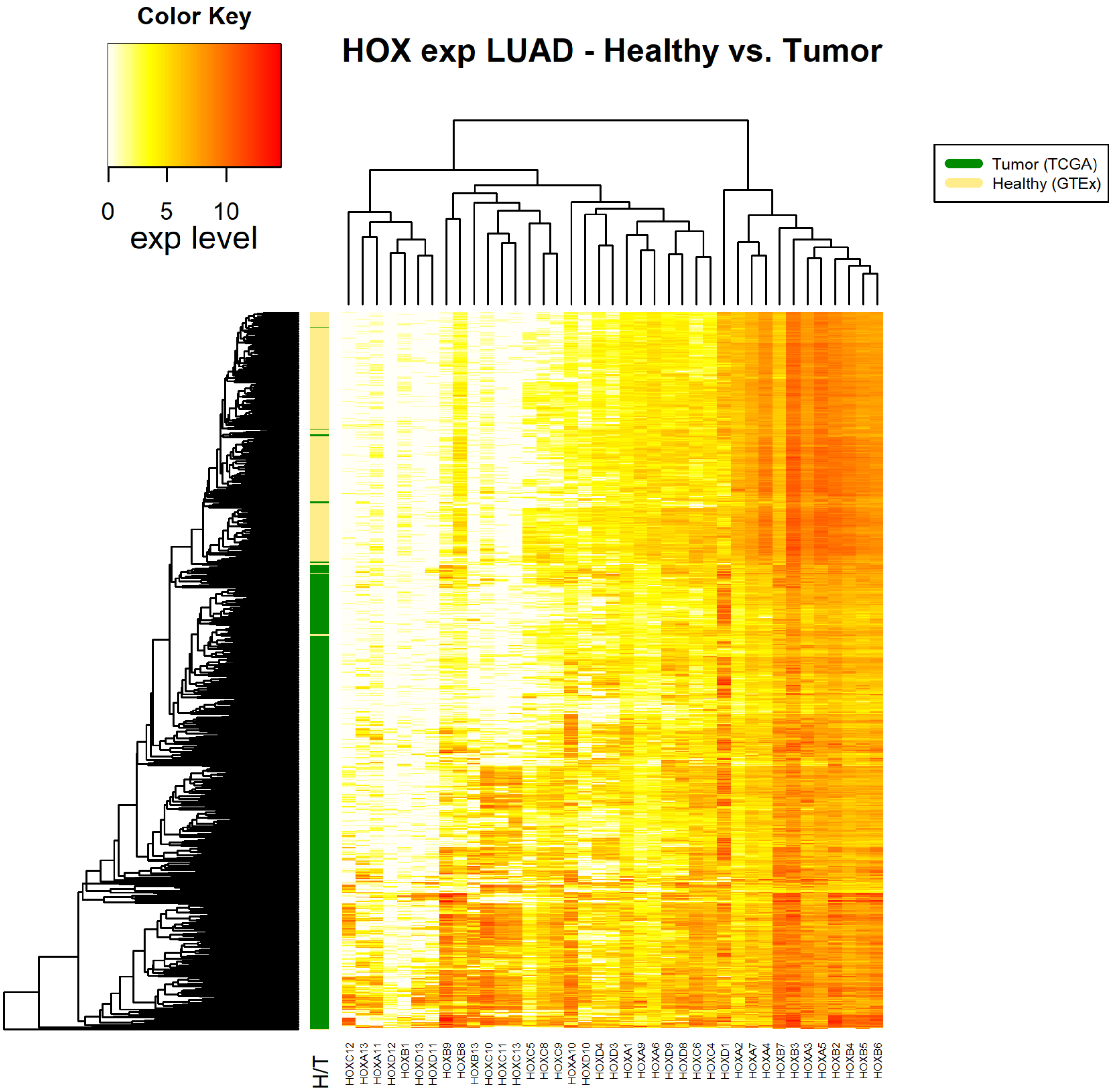

Figure S7. *HOX* gene expression in samples of healthy lung tissue and lung adenocarcinoma (LUAD) tumor tissue. The source of expression data of healthy and tumor samples is GTEx and TCGA, respectively. H/T stands for healthy/tumor.

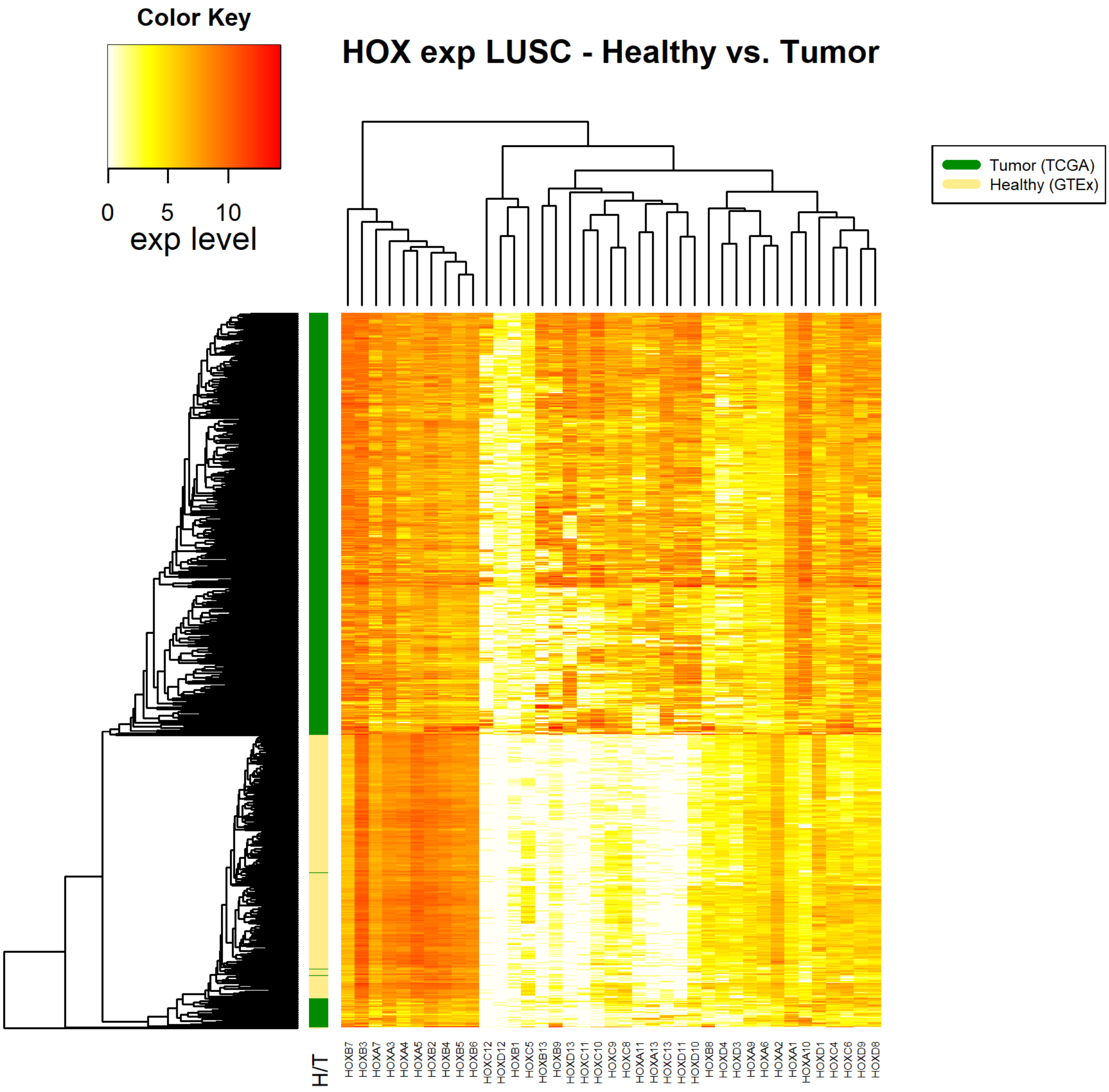

Figure S8. *HOX* gene expression in samples of healthy lung tissue and lung squamous cell carcinoma (LUSC) tumor tissue. The source of expression data of healthy and tumor samples is GTEx and TCGA, respectively. H/T stands for healthy/tumor.

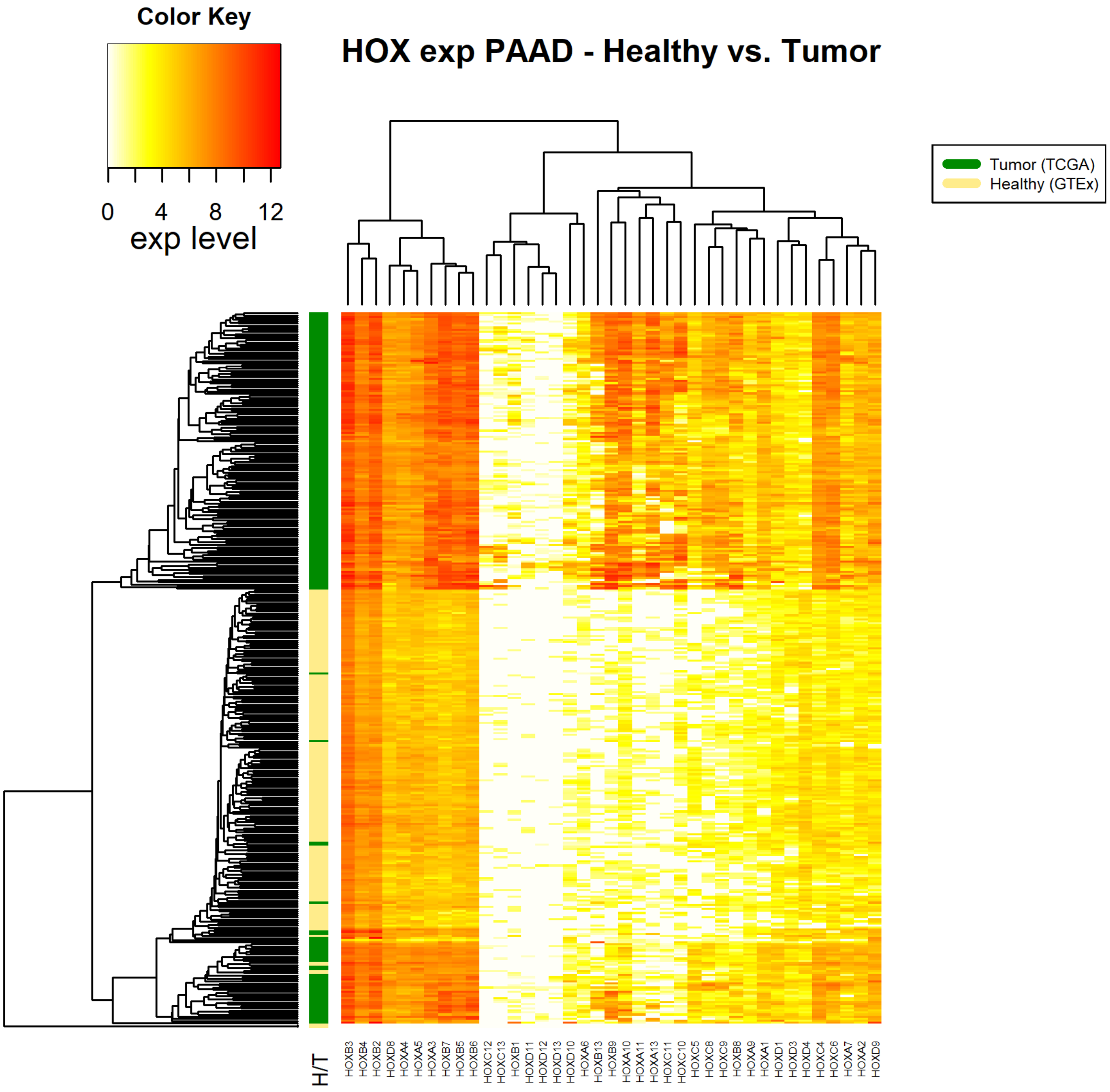

Figure S9. *HOX* gene expression in samples of healthy pancreas tissue and pancreatic adenocarcinoma (PAAD) tumor tissue. The source of expression data of healthy and tumor samples is GTEx and TCGA, respectively. H/T stands for healthy/tumor.

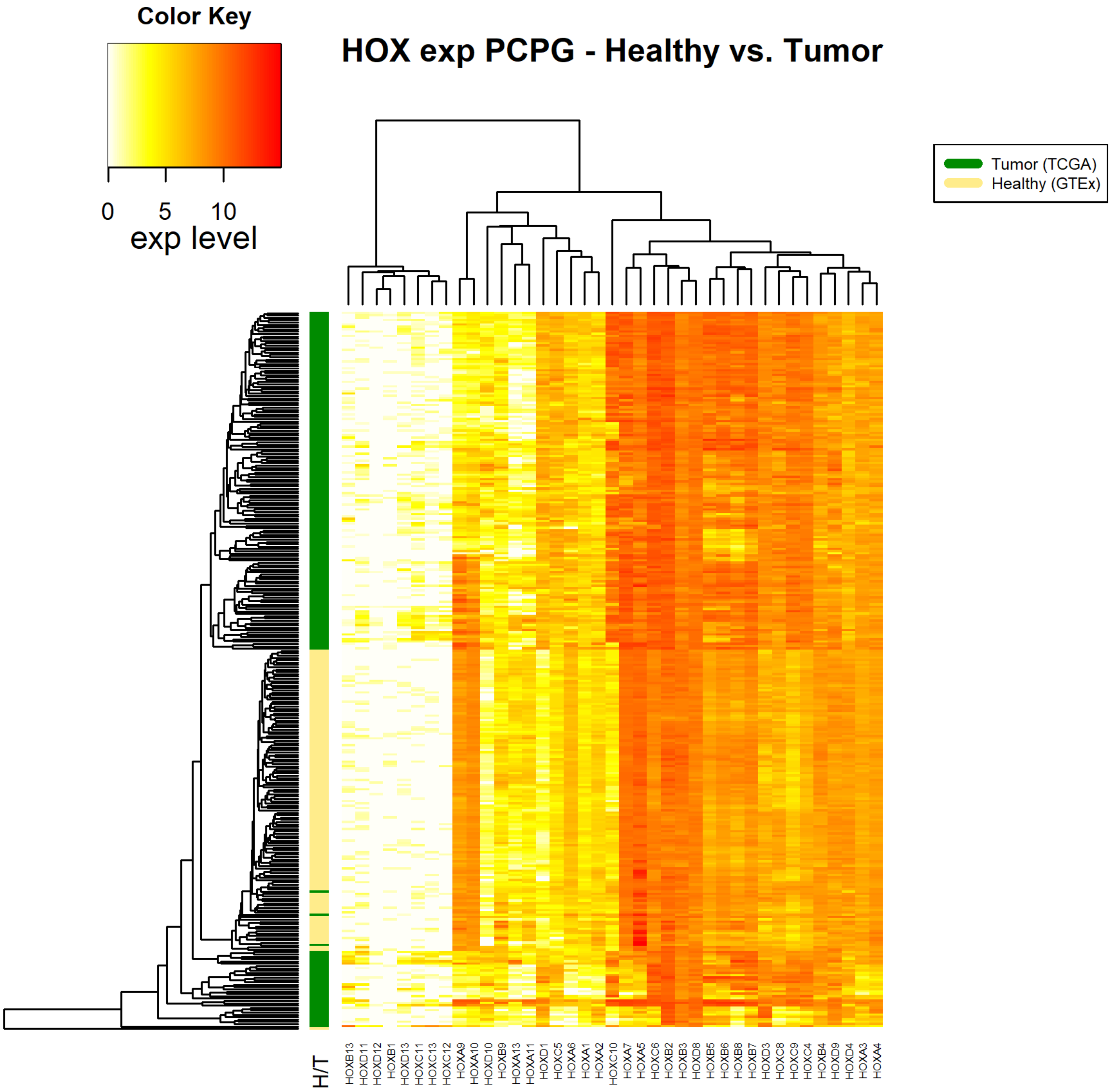

Figure S10. *HOX* gene expression in samples of healthy adrenal gland tissue and pheochromocytoma and paraganglioma (PCPG) tumor tissue. The source of expression data of healthy and tumor samples is GTEx and TCGA, respectively. H/T stands for healthy/tumor.

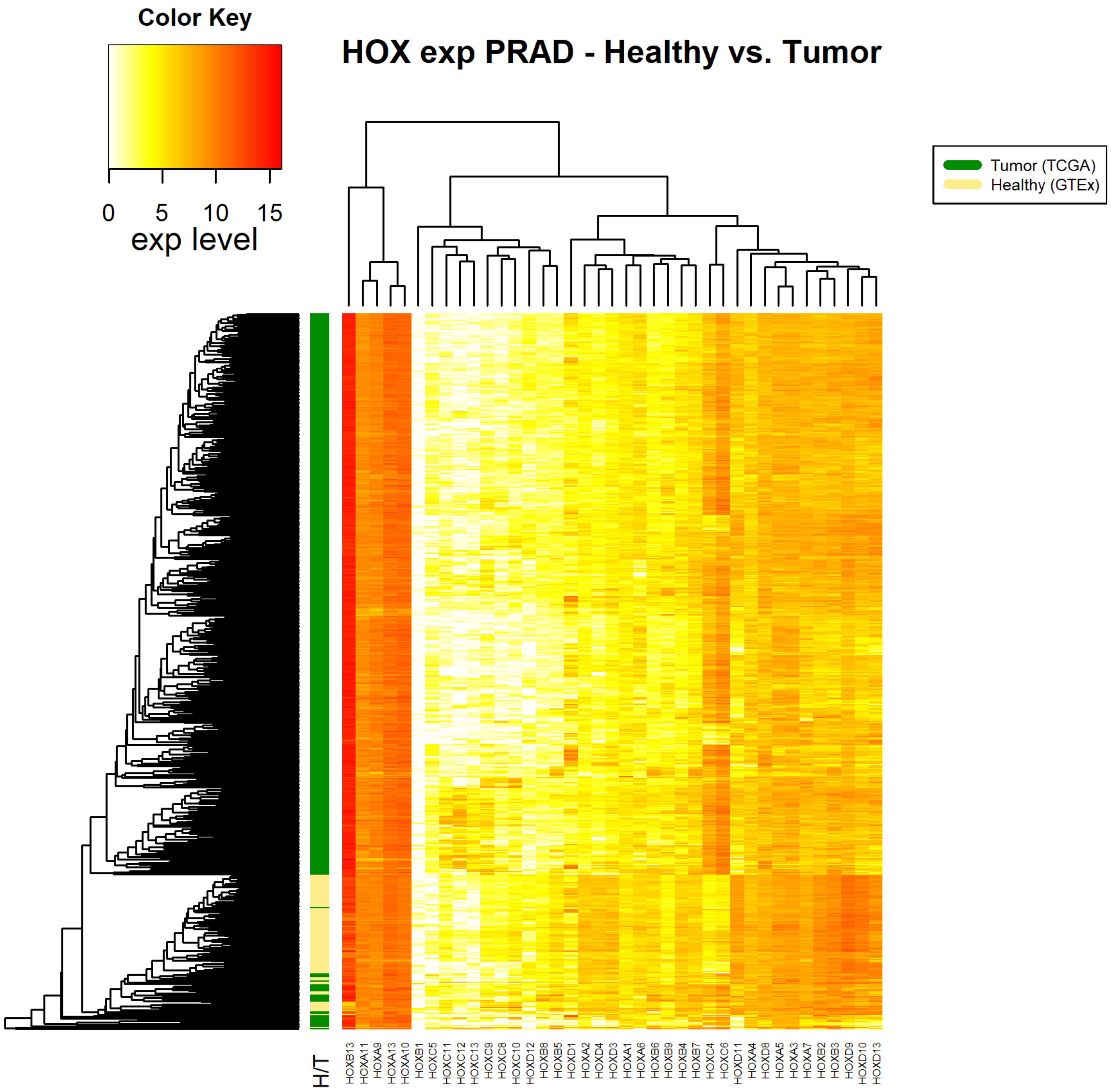

Figure S11. *HOX* gene expression in samples of healthy prostate tissue and prostate adenocarcinoma (PRAD) tumor tissue. The source of expression data of healthy and tumor samples is GTEx and TCGA, respectively. H/T stands for healthy/tumor.

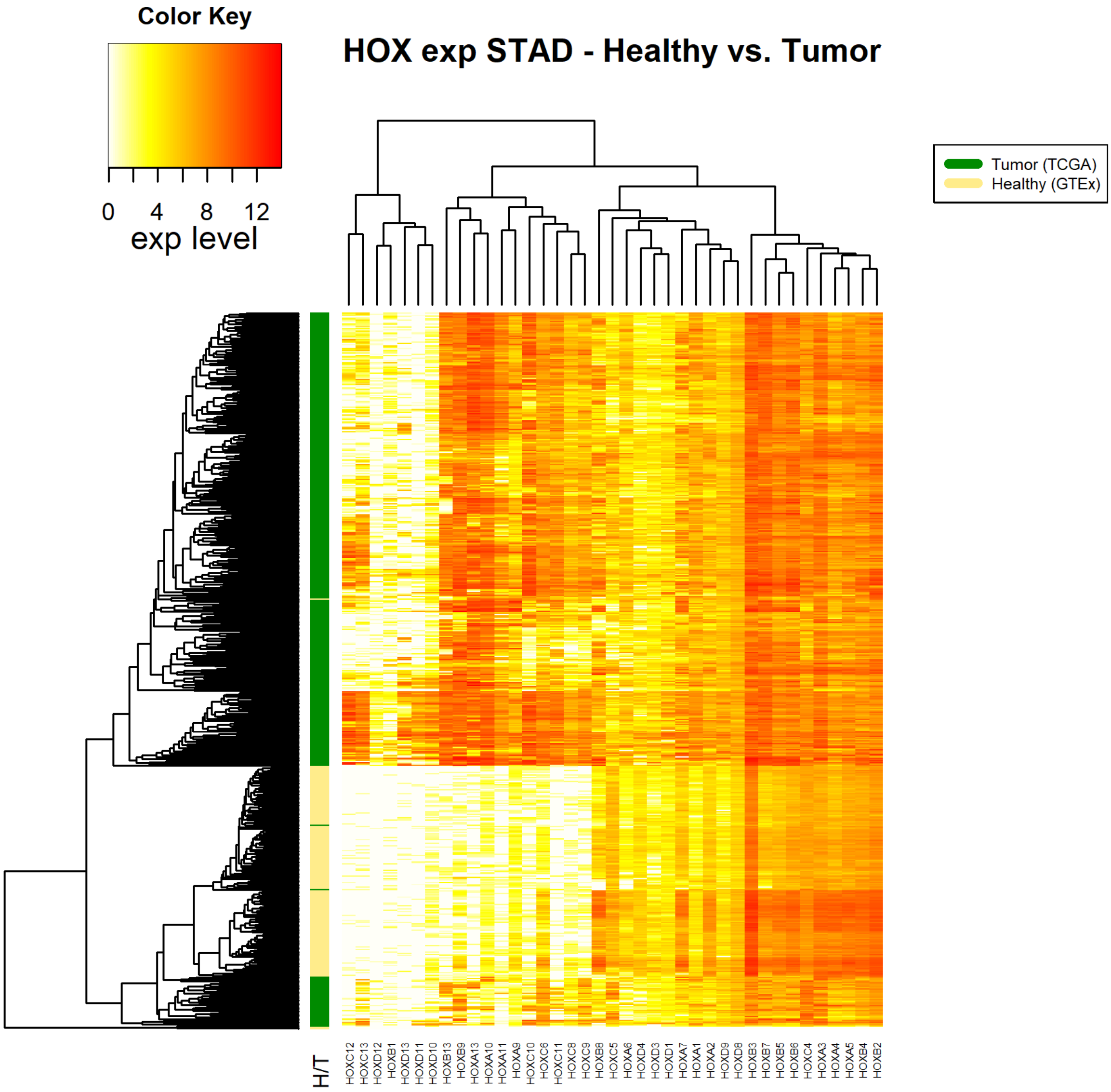

Figure S12. *HOX* gene expression in samples of healthy stomach tissue and stomach adenocarcinoma (STAD) tumor tissue. The source of expression data of healthy and tumor samples is GTEx and TCGA, respectively. H/T stands for healthy/tumor.

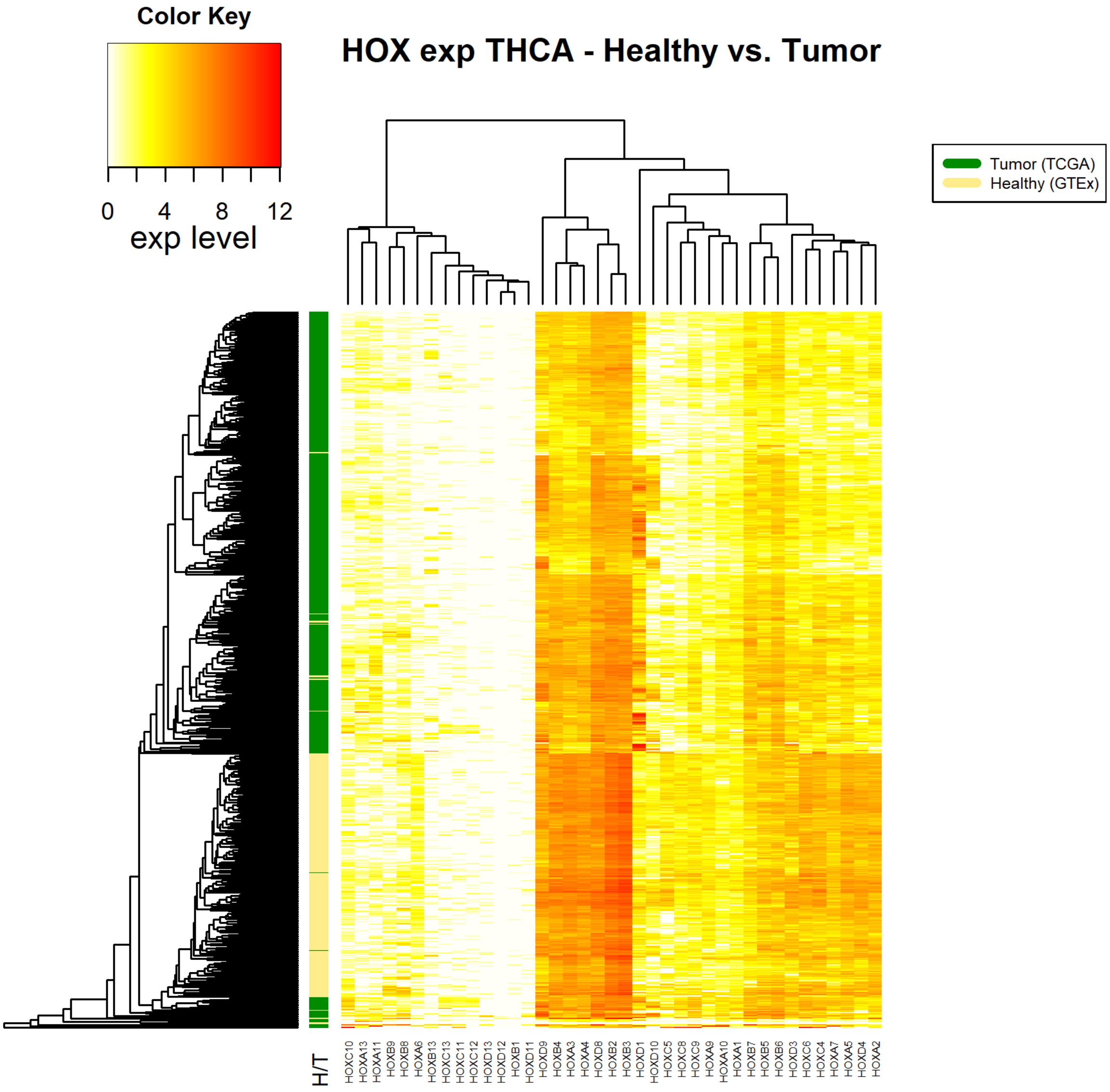

Figure S13. *HOX* gene expression in samples of healthy thyroid tissue and tumor thyroid carcinoma (THCA). The source of expression data of healthy and tumor samples is GTEx and TCGA, respectively. H/T stands for healthy/tumor.

median expression - GTEx																					
Cancer type\HOX	HOXA1	HOXA2	HOXA3	HOXA4	HOXA5	HOXA6	HOXA7	HOXA9	HOXA10	HOXA11	HOXA13	HOXB1	HOXB2	HOXB3	HOXB4	HOXB5	HOXB6	HOXB7	HOXB8	HOXB9	HOXB13
BRCA	5.3	6.6	8.9	8.2	9.0	5.9	9.0	8.5	8.5	4.5	3.7	0.0	9.7	10.6	8.5	6.4	7.4	8.6	6.2	4.5	0.0
COAD	5.5	7.5	9.4	8.0	9.3	6.7	8.9	10.0	10.6	10.0	10.1	1.2	10.4	11.5	9.4	9.0	9.0	8.1	5.7	6.2	5.9
ESCA	7.3	6.9	8.7	8.7	7.6	3.1	6.6	4.6	3.4	0.0	0.0	4.2	9.7	10.0	8.2	3.5	3.8	3.3	0.0	0.0	1.0
GBM	0.0	0.0	0.2	0.0	1.1	0.0	0.0	1.1	1.3	0.0	0.0	0.0	2.7	3.0	1.3	0.0	0.8	0.0	0.0	0.0	0.0
LAML	4.5	3.2	2.9	3.7	3.4	0.0	2.5	5.1	4.9	0.0	0.0	0.0	7.7	7.0	5.9	0.0	1.7	3.1	0.0	2.1	0.0
LGG	0.0	0.0	0.2	0.0	1.1	0.0	0.0	1.1	1.3	0.0	0.0	0.0	2.7	3.0	1.3	0.0	0.8	0.0	0.0	0.0	0.0
LIHC	3.0	3.3	4.4	5.0	4.0	0.0	1.4	2.1	2.0	0.0	0.0	0.0	7.2	8.0	6.3	4.7	4.9	3.4	0.0	0.0	0.0
LUAD	4.2	6.4	8.3	8.4	9.6	4.9	7.2	4.5	3.1	0.0	0.0	0.0	9.1	9.9	8.7	8.1	8.0	6.2	3.4	1.2	0.0
LUSC	4.2	6.4	8.3	8.4	9.6	4.9	7.2	4.5	3.1	0.0	0.0	0.0	9.1	9.9	8.7	8.1	8.0	6.2	3.4	1.2	0.0
PAAD	2.4	3.9	5.2	5.5	5.7	1.4	3.4	2.5	2.1	0.0	0.0	0.0	7.1	8.2	6.7	5.3	5.6	4.9	1.7	0.0	0.0
PCPG	4.7	5.5	7.4	7.8	10.4	6.5	9.9	8.1	8.6	5.2	4.4	0.0	9.9	9.7	8.1	7.5	8.3	8.5	7.9	4.9	0.0
PRAD	5.1	6.6	8.5	7.1	8.2	5.8	8.0	9.6	10.4	9.9	10.9	0.0	9.0	9.5	6.3	5.3	5.9	6.2	4.2	4.3	12.5
STAD	4.0	6.1	7.5	7.2	7.8	3.9	6.2	2.8	2.2	0.0	0.0	0.0	8.4	9.6	7.9	6.5	7.4	6.4	5.5	1.5	0.0
THCA	2.8	5.4	6.9	6.5	5.5	2.1	4.7	3.3	2.4	0.0	0.0	0.0	7.9	8.5	6.5	4.6	4.8	3.9	1.2	0.8	0.0

median expression -TCGA																					
Cancer type\HOX	HOXA1	HOXA2	HOXA3	HOXA4	HOXA5	HOXA6	HOXA7	HOXA9	HOXA10	HOXA11	HOXA13	HOXB1	HOXB2	HOXB3	HOXB4	HOXB5	HOXB6	HOXB7	HOXB8	HOXB9	HOXB13
BRCA	5.0	4.9	6.0	5.1	5.5	3.9	5.7	5.7	6.7	3.9	3.2	1.9	9.3	9.9	8.5	5.5	6.9	7.5	3.5	5.1	2.4
COAD	5.9	5.6	8.3	5.5	7.8	5.7	8.8	10.3	10.7	8.9	10.4	0.0	6.7	10.2	7.7	9.2	9.6	9.6	8.9	11.7	8.2
ESCA	7.8	6.0	9.0	7.6	7.6	5.5	8.6	6.8	9.3	6.7	7.6	2.2	8.9	9.8	8.0	7.3	7.8	9.4	4.9	8.0	7.1
GBM	5.7	5.4	6.9	7.5	7.6	4.9	8.2	4.1	8.0	4.9	5.3	0.0	7.7	8.5	5.5	3.7	3.8	7.0	2.6	3.3	4.2
LAML	5.9	5.9	8.5	7.3	8.8	7.5	9.1	11.0	10.1	2.2	0.0	0.0	8.5	10.2	9.0	6.2	5.1	3.1	1.2	1.6	0.0
LGG	2.2	0.0	0.7	1.1	0.9	0.0	1.7	0.8	2.4	1.4	2.5	0.0	3.4	2.9	1.9	0.7	1.6	3.7	0.0	0.0	0.6
LIHC	2.6	2.6	5.4	4.7	4.3	0.0	2.2	1.8	4.9	0.9	6.8	0.0	5.9	6.2	5.0	4.7	4.7	4.8	1.0	1.0	0.0
LUAD	4.5	4.5	6.9	5.2	7.4	3.8	5.4	3.3	4.2	1.0	0.9	0.0	7.5	8.6	6.9	6.7	6.9	7.6	3.1	2.8	0.0
LUSC	7.4	4.5	8.0	6.7	6.9	4.3	6.8	4.6	8.6	5.2	6.1	1.5	7.6	8.9	6.8	6.4	6.9	9.0	4.2	5.6	5.7
PAAD	5.4	5.6	8.2	6.6	7.1	2.9	5.0	4.1	7.2	4.3	6.6	0.6	9.1	9.9	8.0	8.2	9.0	9.1	5.0	7.4	4.3
PCPG	5.1	5.0	7.6	7.7	9.4	5.9	10.2	4.4	4.7	2.7	2.2	0.0	10.8	9.1	8.1	8.9	9.6	10.1	9.3	4.2	0.0
PRAD	5.0	4.7	7.7	6.1	7.5	5.6	7.2	9.8	11.1	9.4	11.2	0.0	7.1	7.6	5.1	2.8	3.9	5.8	2.8	4.5	14.3
STAD	6.9	6.1	8.9	7.2	7.7	5.2	6.1	5.6	9.3	7.1	9.4	1.2	8.4	9.7	7.7	8.3	8.7	9.5	6.0	9.0	8.2
THCA	2.4	3.1	4.4	4.8	3.1	0.0	2.7	1.4	2.4	1.3	1.3	0.0	5.9	5.9	4.8	3.8	4.6	4.1	0.7	0.5	0.0

a. HOX A and B.

median expression - GTEx<br>Cancer type\HOX	HOXC4	HOXC5	HOXC6	HOXC8	HOXC9	HOXC10	HOXC11	HOXC12	HOXC13	HOXD1	HOXD3	HOXD4	HOXD8	HOXD9	HOXD10	HOXD11	HOXD12	HOXD13
BRCA	7.7	5.4	9.3	7.9	8.0	6.1	2.0	0.0	1.7	6.1	7.6	8.1	9.4	9.2	6.8	3.1	0.0	1.0
COAD	5.7	2.3	3.9	1.9	2.2	2.3	0.0	0.0	0.0	5.9	5.9	7.7	8.4	8.9	8.5	7.3	0.0	5.5
ESCA	8.2	5.2	6.9	3.3	3.8	2.0	0.0	0.0	1.5	4.6	5.2	5.2	6.1	6.8	4.3	1.2	0.0	0.0
GBM	1.4	0.0	1.1	0.0	0.0	0.9	0.0	0.0	0.0	4.4	1.4	0.0	0.0	0.0	0.0	0.0	0.0	0.0
LAML	4.6	0.0	1.4	0.0	0.0	1.6	0.0	0.0	0.0	0.0	0.0	0.0	0.0	0.0	0.0	0.0	0.0	0.0
LGG	1.4	0.0	1.1	0.0	0.0	0.9	0.0	0.0	0.0	4.4	1.4	0.0	0.0	0.0	0.0	0.0	0.0	0.0
LIHC	2.6	0.0	1.2	0.0	0.0	1.5	0.0	0.0	0.0	0.0	0.0	1.5	2.3	1.7	0.0	0.0	0.0	0.0
LUAD	4.7	1.6	4.5	2.0	2.0	1.2	0.0	0.0	0.0	5.7	3.2	3.8	4.8	4.7	2.0	0.0	0.0	0.0
LUSC	4.7	1.6	4.5	2.0	2.0	1.2	0.0	0.0	0.0	5.7	3.2	3.8	4.8	4.7	2.0	0.0	0.0	0.0
PAAD	3.6	0.0	4.1	1.6	1.7	1.7	0.0	0.0	0.0	3.5	3.2	4.5	5.2	3.8	0.0	0.0	0.0	0.0
PCPG	7.1	4.9	9.0	7.3	5.9	4.6	0.0	0.0	0.0	3.0	7.0	8.1	9.2	8.0	2.0	0.0	0.0	0.0
PRAD	4.4	0.0	5.1	3.4	3.4	4.3	3.1	1.6	1.2	4.6	6.8	6.6	7.6	10.5	10.0	8.4	3.4	9.1
STAD	7.4	6.2	3.1	1.2	0.0	1.2	0.0	0.0	0.0	4.2	3.7	5.1	5.6	4.3	1.3	0.0	0.0	0.0
THCA	5.3	3.2	5.4	3.0	3.3	1.3	0.0	0.0	0.0	3.1	4.9	5.4	6.9	5.8	2.3	0.0	0.0	0.0

median expression -TCGA<br>Cancer type\HOX	HOXC4	HOXC5	HOXC6	HOXC8	HOXC9	HOXC10	HOXC11	HOXC12	HOXC13	HOXD1	HOXD3	HOXD4	HOXD8	HOXD9	HOXD10	HOXD11	HOXD12	HOXD13
BRCA	7.2	4.5	8.3	6.8	8.1	9.1	7.4	3.6	7.6	4.0	5.6	5.4	7.5	7.4	6.4	2.1	0.0	2.5
COAD	4.5	0.0	5.2	3.2	3.2	1.9	2.9	0.0	0.0	4.1	4.7	4.4	7.6	7.2	5.7	5.3	0.0	5.9
ESCA	7.8	4.4	7.9	6.7	7.0	9.2	7.3	3.9	7.7	4.7	4.7	4.1	7.0	6.9	7.3	6.5	1.4	4.2
GBM	7.6	3.9	6.4	4.8	5.8	7.8	5.2	0.0	5.0	3.8	6.1	5.1	6.6	7.0	7.7	6.9	2.7	6.7
LAML	2.4	0.0	0.0	0.0	0.0	0.0	0.0	0.0	0.0	0.0	0.0	0.0	0.0	0.0	0.0	0.0	0.0	0.0
LGG	4.8	0.0	0.8	0.0	0.0	0.8	0.0	0.0	0.0	5.2	3.4	2.9	5.1	2.0	1.1	0.0	0.0	0.0
LIHC	2.5	0.0	1.3	0.0	1.2	0.0	0.0	0.0	0.0	2.9	2.7	2.4	5.1	5.2	2.1	0.0	0.0	0.0
LUAD	4.9	1.7	4.8	2.7	3.3	1.3	1.8	0.0	1.0	7.7	4.1	2.2	5.1	4.4	3.0	0.0	0.0	0.0
LUSC	6.0	3.1	7.1	6.4	5.8	5.9	5.4	0.9	7.2	6.0	4.2	3.3	6.7	6.7	7.2	7.0	1.6	7.1
PAAD	7.0	4.5	7.1	5.5	5.8	4.9	5.4	0.0	0.7	4.4	4.0	3.7	6.7	6.1	1.9	0.0	0.0	0.0
PCPG	9.4	6.7	10.9	8.6	9.5	9.5	1.1	0.0	0.0	6.8	8.7	6.8	9.8	8.1	4.0	0.0	0.0	0.0
PRAD	7.2	2.4	8.6	1.6	3.4	3.2	2.2	1.7	1.4	3.8	4.6	3.8	7.1	7.5	7.5	6.4	2.6	7.8
STAD	7.8	4.8	7.4	5.6	6.6	8.7	7.4	2.1	3.4	4.4	4.1	3.9	6.4	5.7	2.4	0.0	0.0	0.0
THCA	3.3	1.2	3.0	1.1	2.1	0.7	0.0	0.0	0.0	4.1	3.3	2.6	6.1	5.2	1.4	0.0	0.0	0.0

b. HOX C and D.

Figure S14. HOX genes median expression obtained from GTEx or TCGA, categorized by cancer type.

Figures S15-S33: Differentially expressed HOX pairs that demonstrate expression correlation in healthy tissue (Pearson correlation coefficient > 0.4) and demonstrate expression correlation in tumor tissue (Pearson correlation coefficient > 0.4)

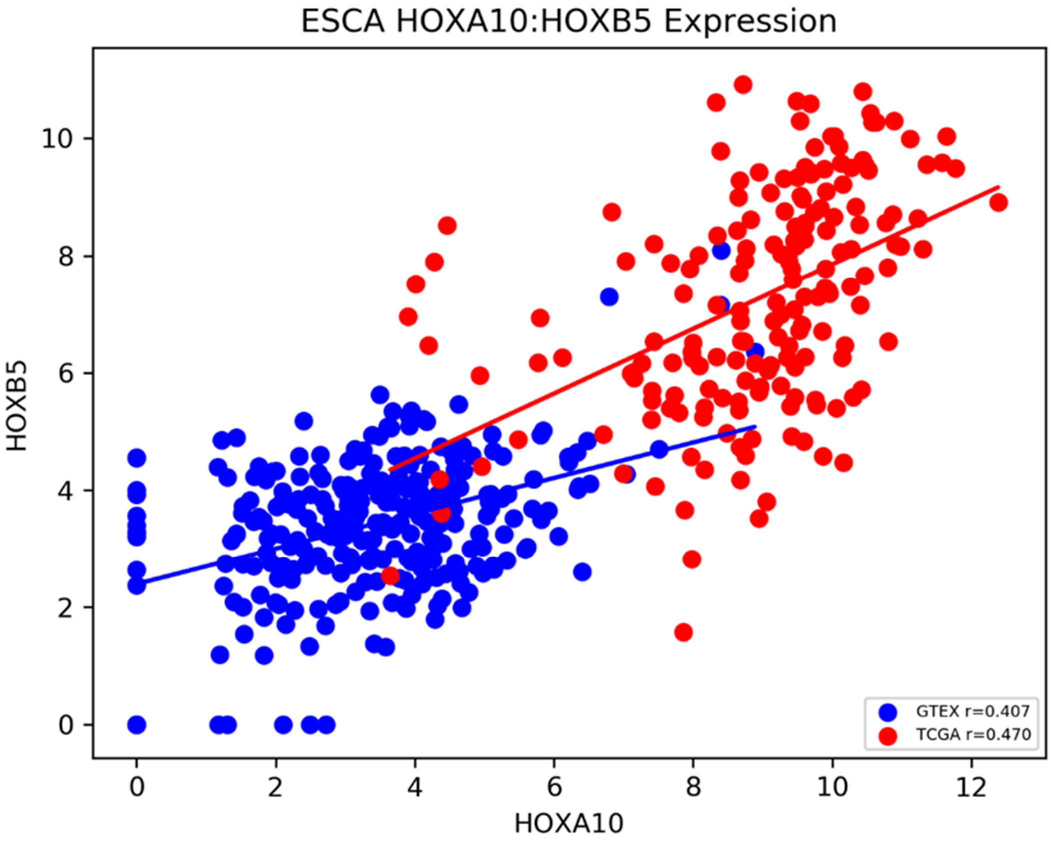

Figure S15. Expression correlation between *HOXA10* and *HOXB5* in samples of healthy esophagus and samples of esophageal carcinoma (ESCA) tumor tissue. The source of expression data of healthy and tumor samples is GTEx and TCGA, respectively. Pierson correlation coefficient (r) indicated in the figure legend.

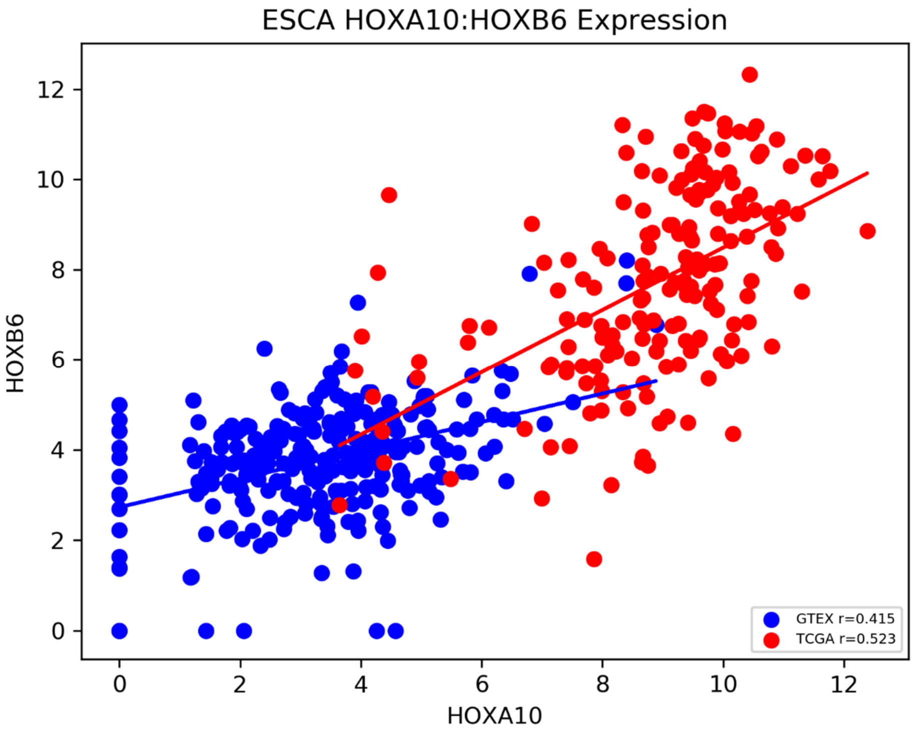

Figure S16. Expression correlation between *HOXA10* and *HOXB6* in samples of healthy esophagus and samples of esophageal carcinoma (ESCA) tumor tissue. The source of expression data of healthy and tumor samples is GTEx and TCGA, respectively. Pierson correlation coefficient (r) indicated in the figure legend.

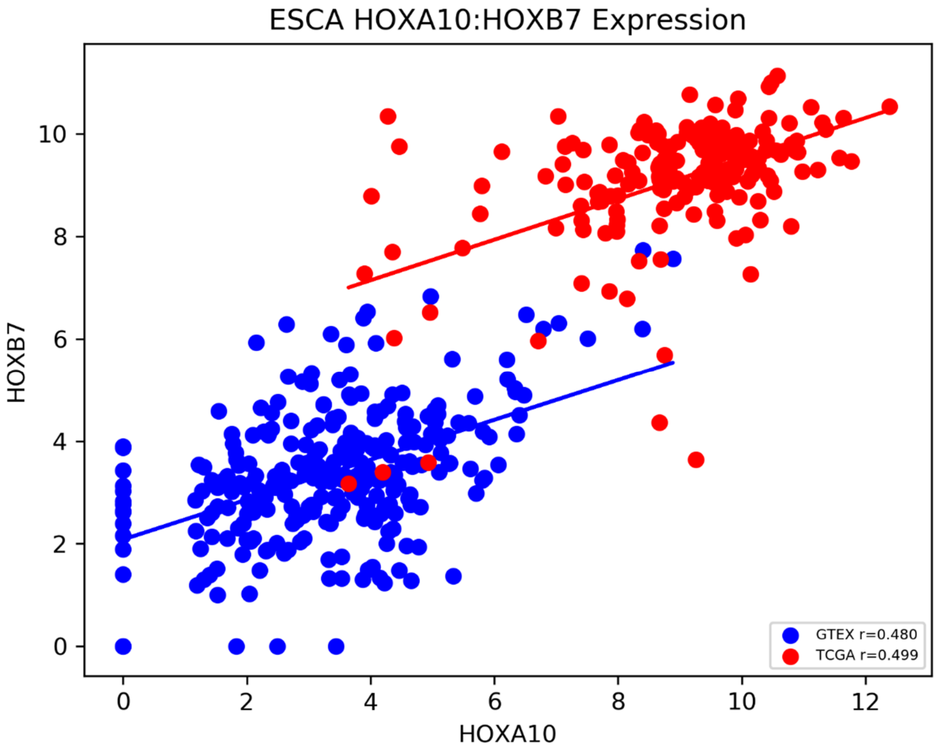

Figure S17. Expression correlation between *HOXA10* and *HOXB7* in samples of healthy esophagus and samples of esophageal carcinoma (ESCA) tumor tissue. The source of expression data of healthy and tumor samples is GTEx and TCGA, respectively. Pierson correlation coefficient (r) indicated in the figure legend.

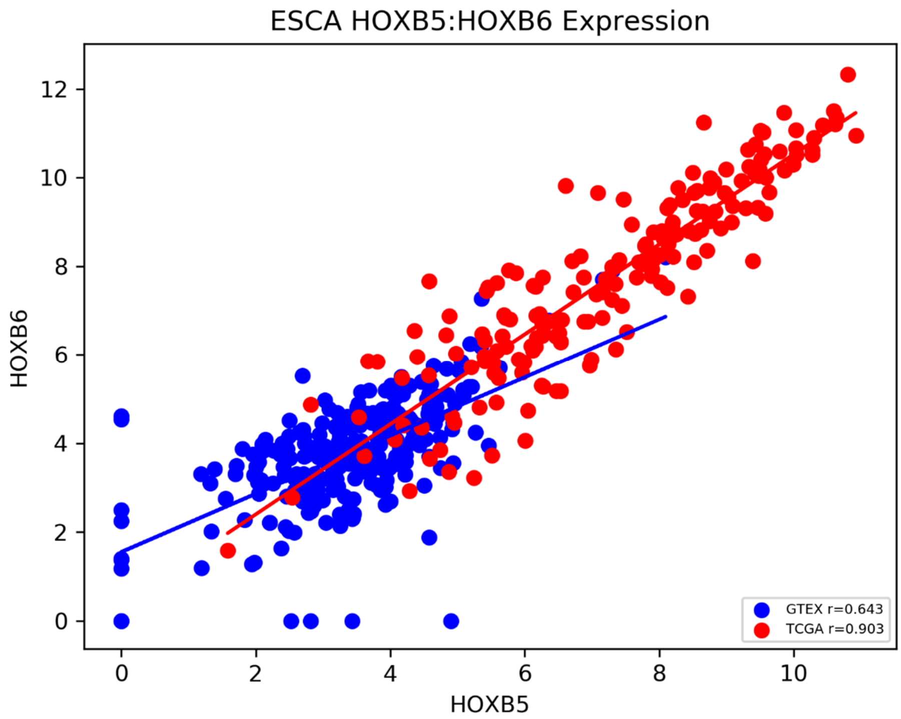

Figure S18. Expression correlation between *HOXB5* and *HOXB6* in samples of healthy esophagus and samples of esophageal carcinoma (ESCA) tumor tissue. The source of expression data of healthy and tumor samples is GTEx and TCGA, respectively. Pierson correlation coefficient (r) indicated in the figure legend.

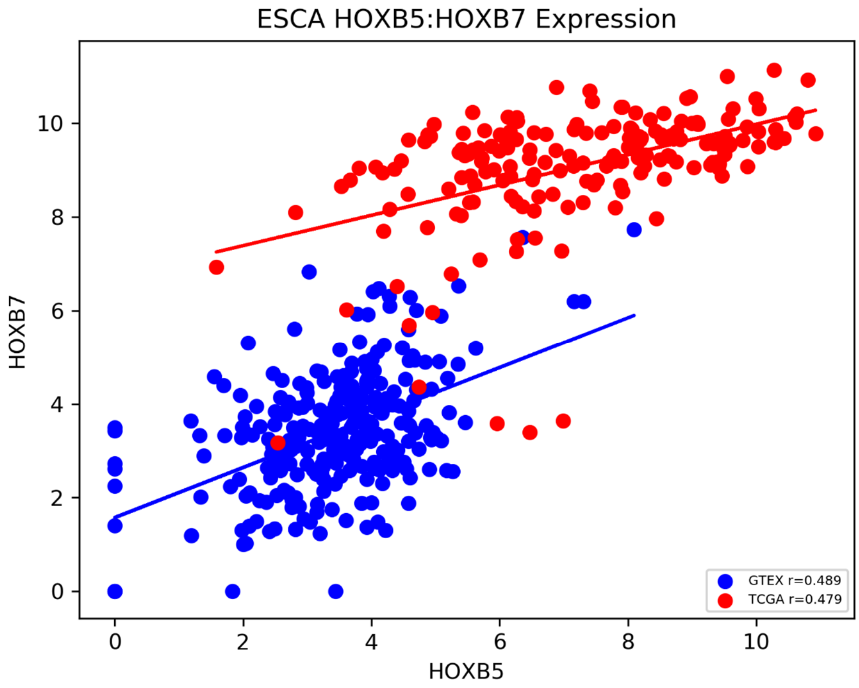

Figure S19. Expression correlation between *HOXB5* and *HOXB7* in samples of healthy esophagus and samples of esophageal carcinoma (ESCA) tumor tissue. The source of expression data of healthy and tumor samples is GTEx and TCGA, respectively. Pierson correlation coefficient (r) indicated in the figure legend.

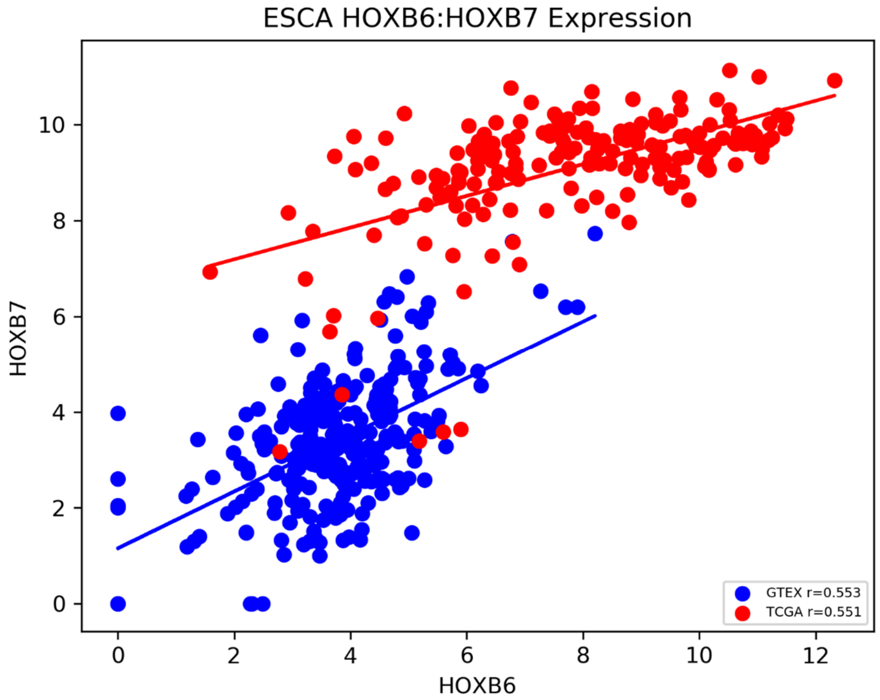

Figure S20. Expression correlation between *HOXB6* and *HOXB7* in samples of healthy esophagus and samples of esophageal carcinoma (ESCA) tumor tissue. The source of expression data of healthy and tumor samples is GTEx and TCGA, respectively. Pierson correlation coefficient (r) indicated in the figure legend.

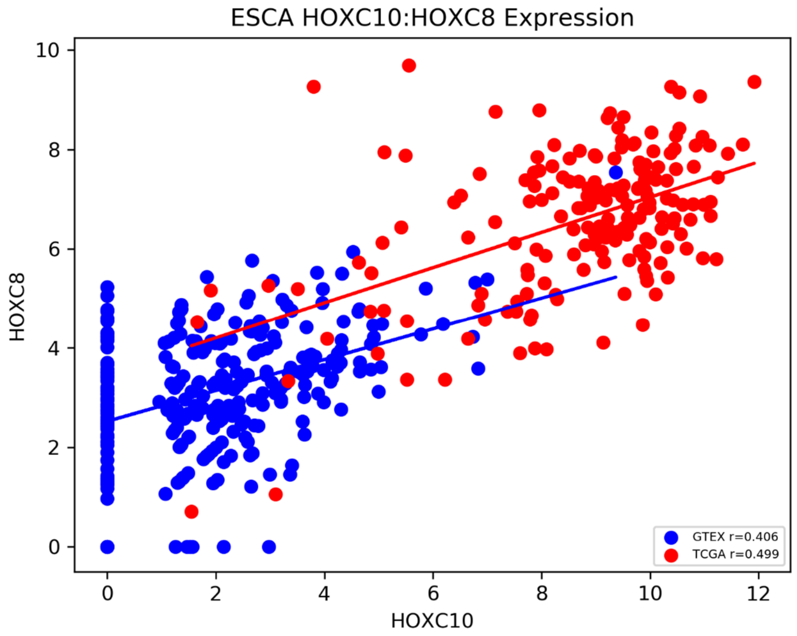

Figure S21. Expression correlation between *HOXC10* and *HOXC8* in samples of healthy esophagus and samples of esophageal carcinoma (ESCA) tumor tissue. The source of expression data of healthy and tumor samples is GTEx and TCGA, respectively. Pierson correlation coefficient (r) indicated in the figure legend.

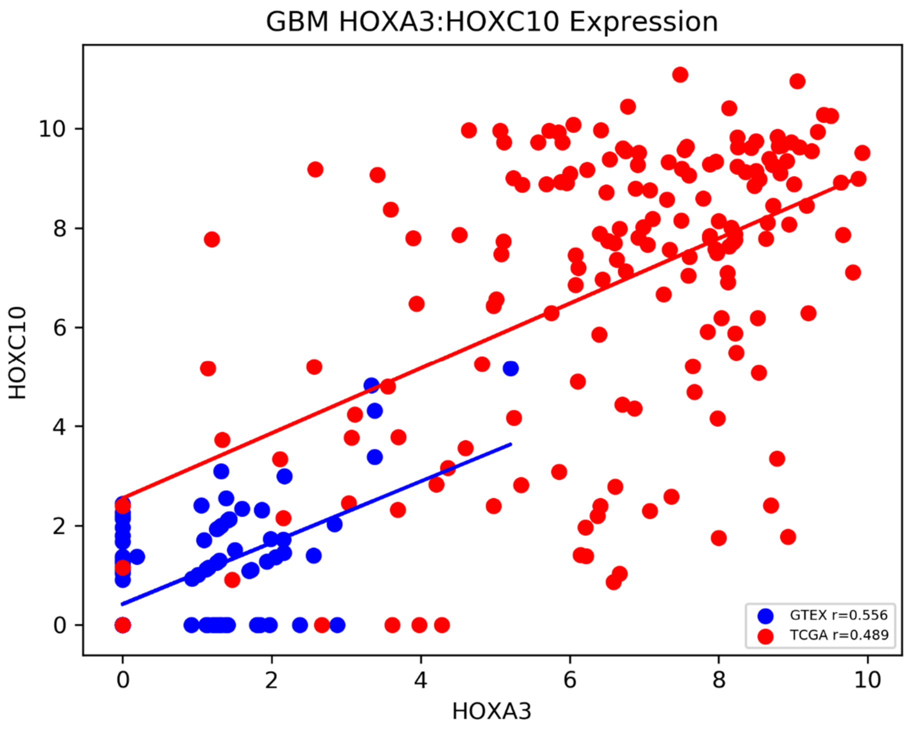

Figure S22. Expression correlation between *HOXA3* and *HOXC10* in samples of in samples of healthy brain tissue and glioblastoma multiforme (GBM) tumor tissue. The source of expression data of healthy and tumor samples is GTEx and TCGA, respectively. Pierson correlation coefficient (r) indicated in the figure legend.

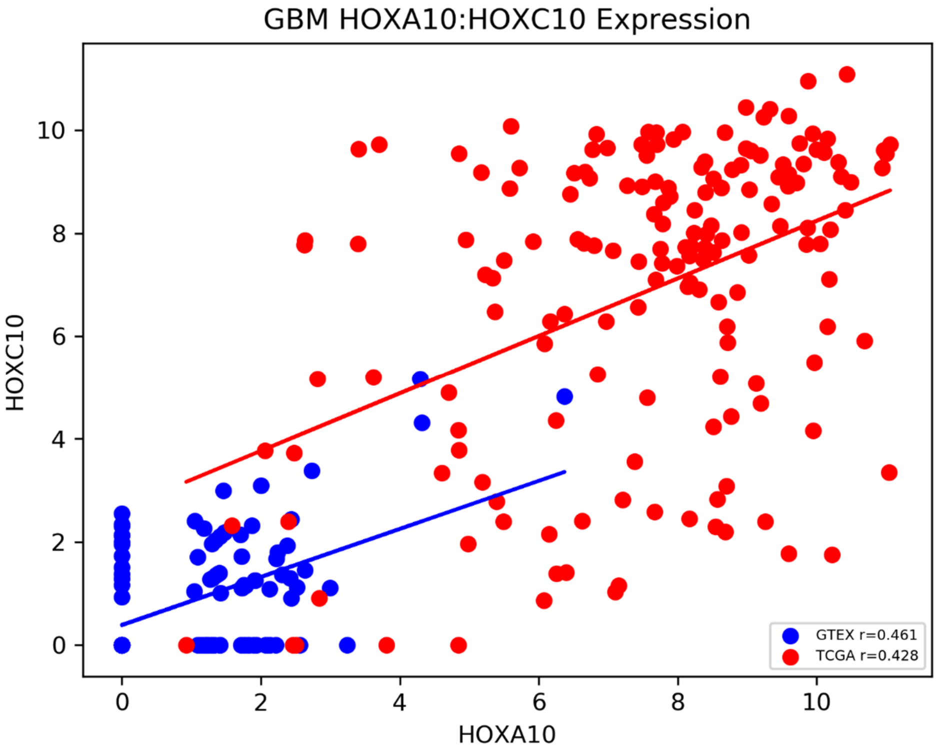

Figure S23. Expression correlation between *HOXA10* and *HOXC10* in samples of in samples of healthy brain tissue and glioblastoma multiforme (GBM) tumor tissue. The source of expression data of healthy and tumor samples is GTEx and TCGA, respectively. Pierson correlation coefficient (r) indicated in the figure legend.

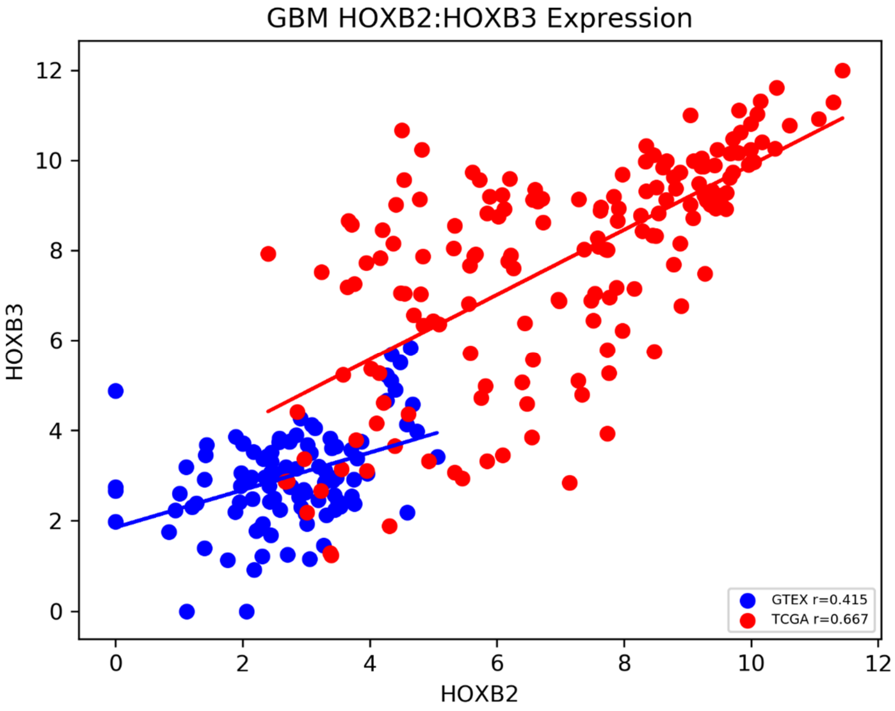

Figure S24. Expression correlation between *HOXB2* and *HOXB3* in samples of in samples of healthy brain tissue and glioblastoma multiforme (GBM) tumor tissue. The source of expression data of healthy and tumor samples is GTEx and TCGA, respectively. Pierson correlation coefficient (r) indicated in the figure legend.

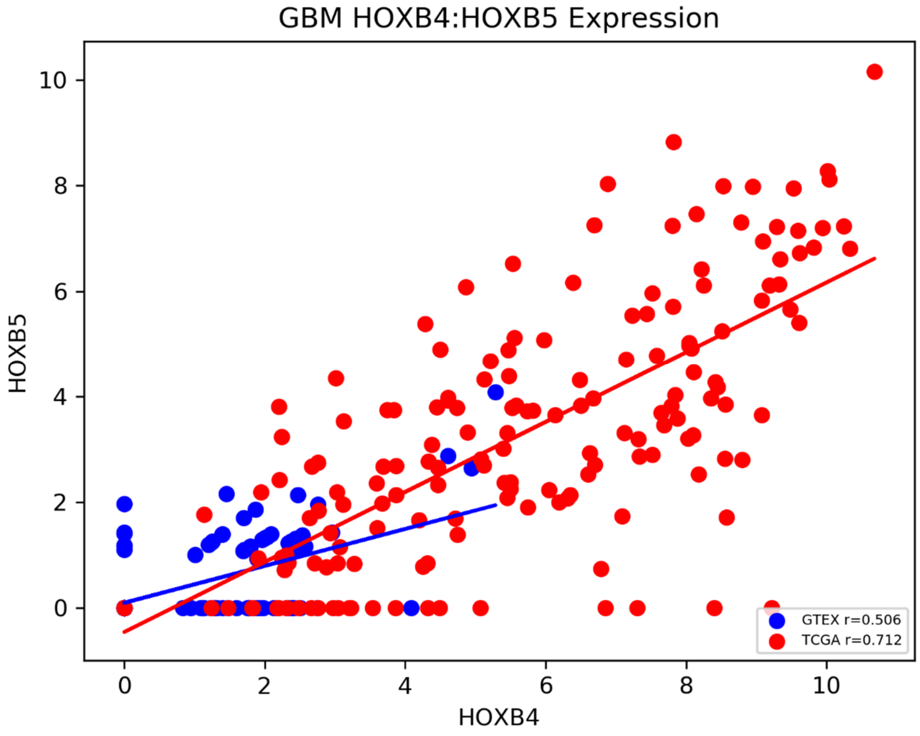

Figure S25. Expression correlation between *HOXB4* and *HOXB5* in samples of in samples of healthy brain tissue and glioblastoma multiforme (GBM) tumor tissue. The source of expression data of healthy and tumor samples is GTEx and TCGA, respectively. Pierson correlation coefficient (r) indicated in the figure legend.

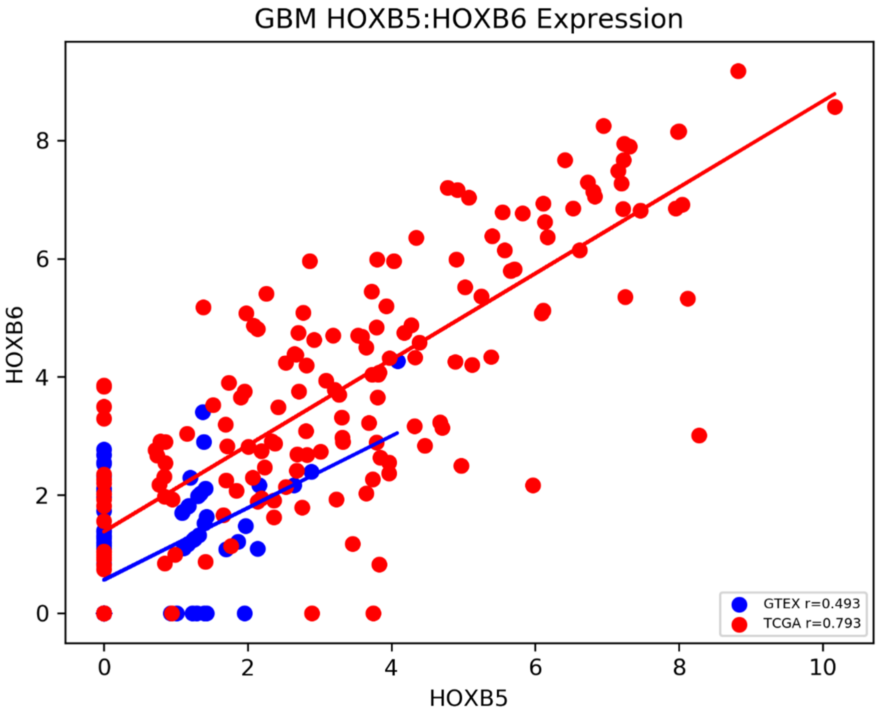

Figure S26. Expression correlation between *HOXB5* and *HOXB6* in samples of in samples of healthy brain tissue and glioblastoma multiforme (GBM) tumor tissue. The source of expression data of healthy and tumor samples is GTEx and TCGA, respectively. Pierson correlation coefficient (r) indicated in the figure legend.

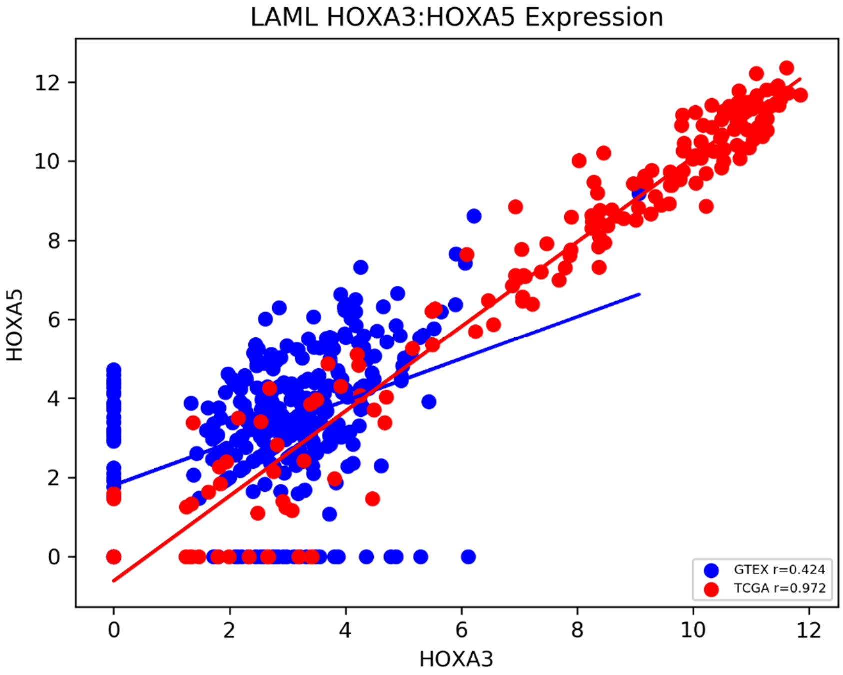

Figure S27. Expression correlation between *HOXA3* and *HOXA5* in samples of healthy blood tissue and acute meloid leukemia (LAML) tumor tissue. The source of expression data of healthy and tumor samples is GTEx and TCGA, respectively. Pierson correlation coefficient (r) indicated in the figure legend.

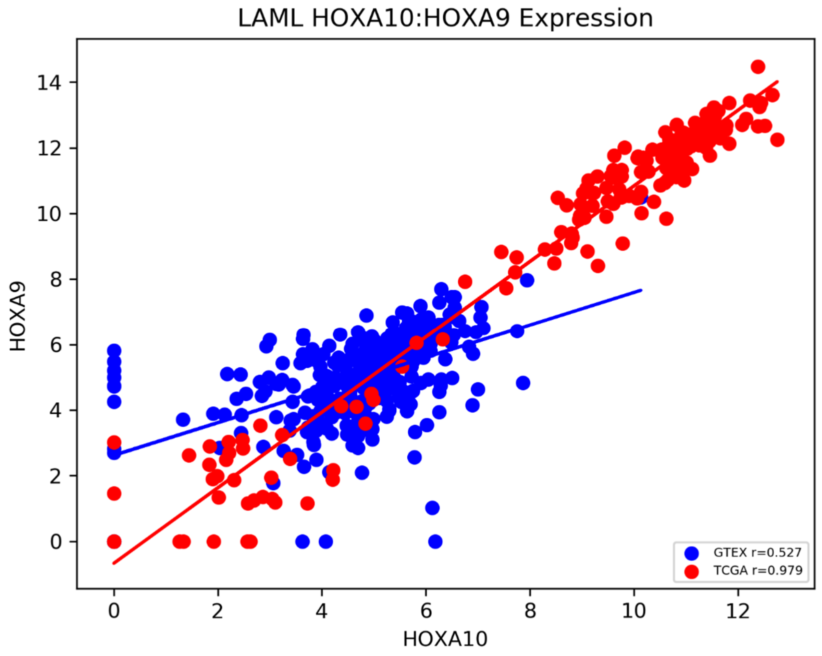

Figure S28. Expression correlation between *HOXA10* and *HOXA9* in samples of healthy blood tissue and acute meloid leukemia (LAML) tumor tissue. The source of expression data of healthy and tumor samples is GTEx and TCGA, respectively. Pierson correlation coefficient (r) indicated in the figure legend.

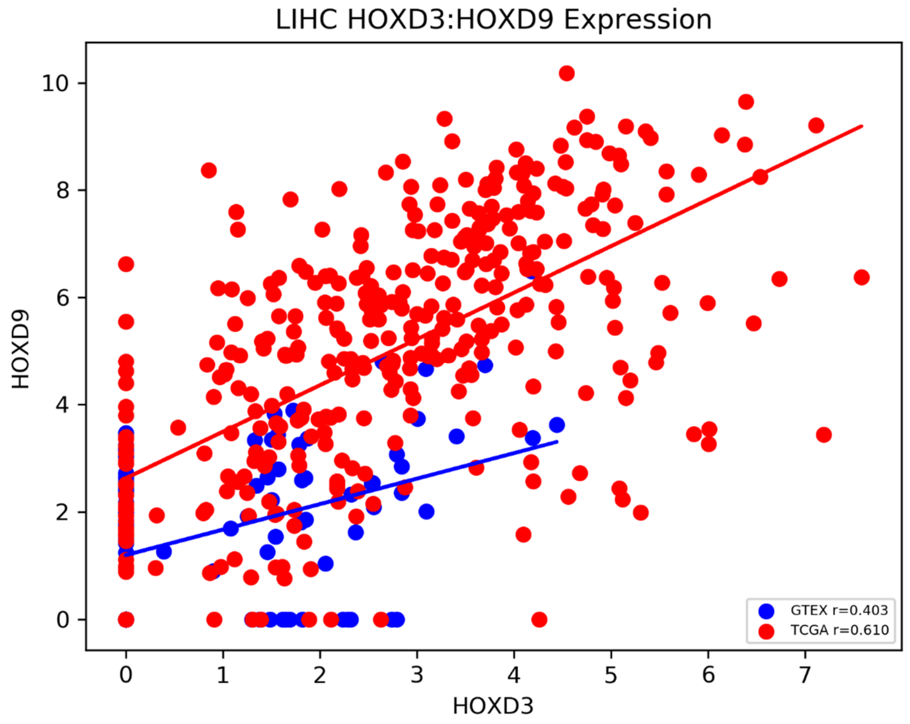

Figure S29. Expression correlation between *HOXD3* and *HOXD9* in samples of healthy liver tissue and liver hepatocellular carcinoma (LIHC) tumor tissue. The source of expression data of healthy and tumor samples is GTEx and TCGA, respectively. Pierson correlation coefficient (r) indicated in the figure legend.

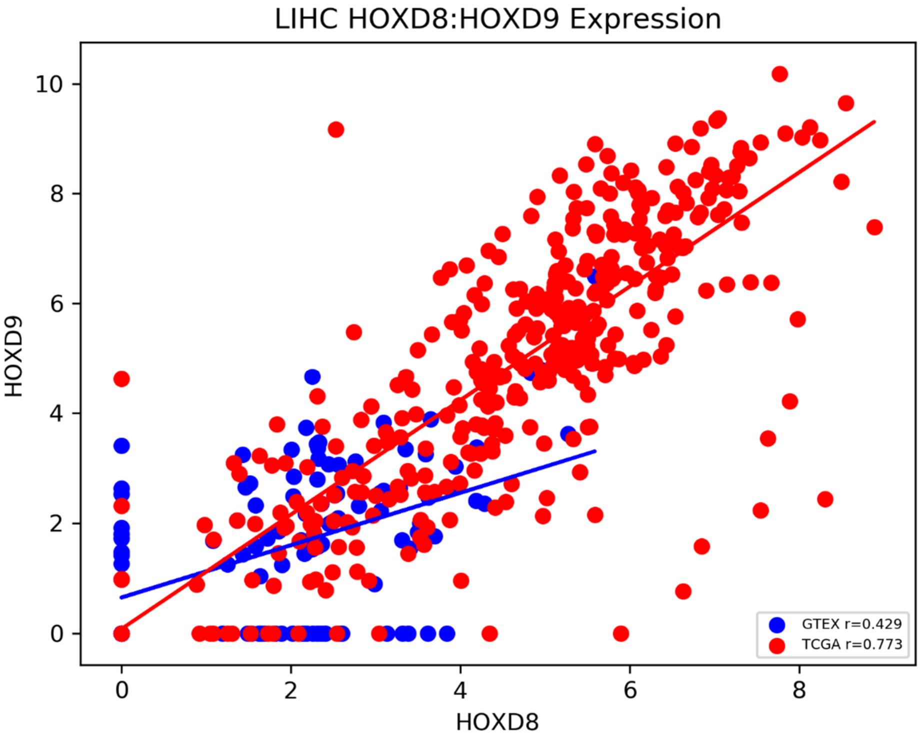

Figure S30. Expression correlation between *HOXD8* and *HOXD9* in samples of healthy liver tissue and liver hepatocellular carcinoma (LIHC) tumor tissue. The source of expression data of healthy and tumor samples is GTEx and TCGA, respectively. Pierson correlation coefficient (r) indicated in the figure legend.

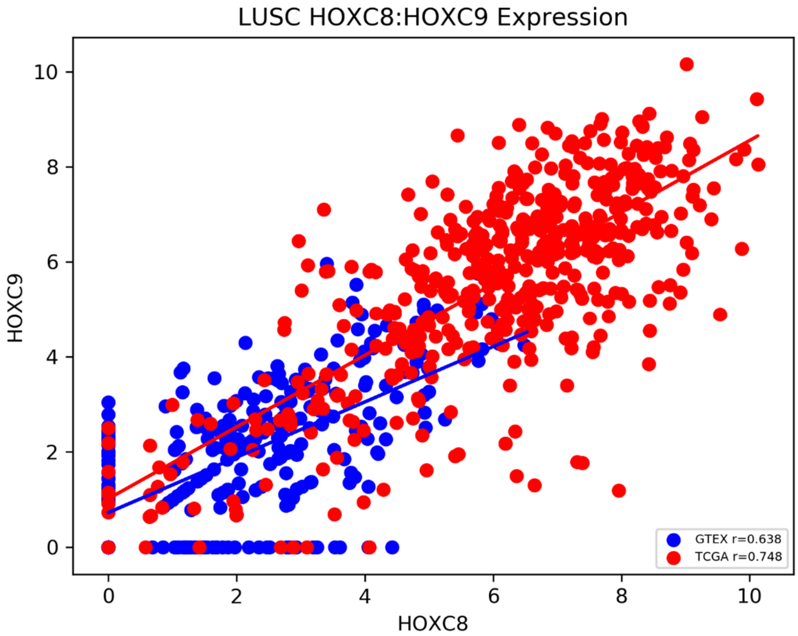

Figure S31. Expression correlation between *HOXC8* and *HOXC9* in samples of healthy lung tissue and lung squamous cell carcinoma (LUSC) tumor tissue. The source of expression data of healthy and tumor samples is GTEx and TCGA, respectively. Pierson correlation coefficient (r) indicated in the figure legend.

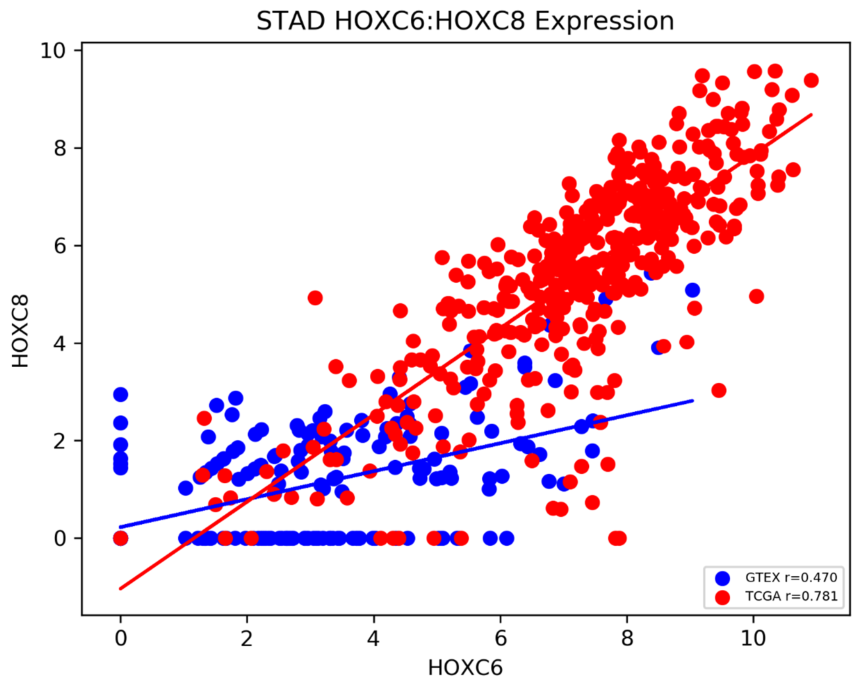

Figure S32. Expression correlation between *HOXC6* and *HOXC8* in samples of healthy stomach tissue and stomach adenocarcinoma (STAD) tumor tissue. The source of expression data of healthy and tumor samples is GTEx and TCGA, respectively. Pierson correlation coefficient (r) indicated in the figure legend.

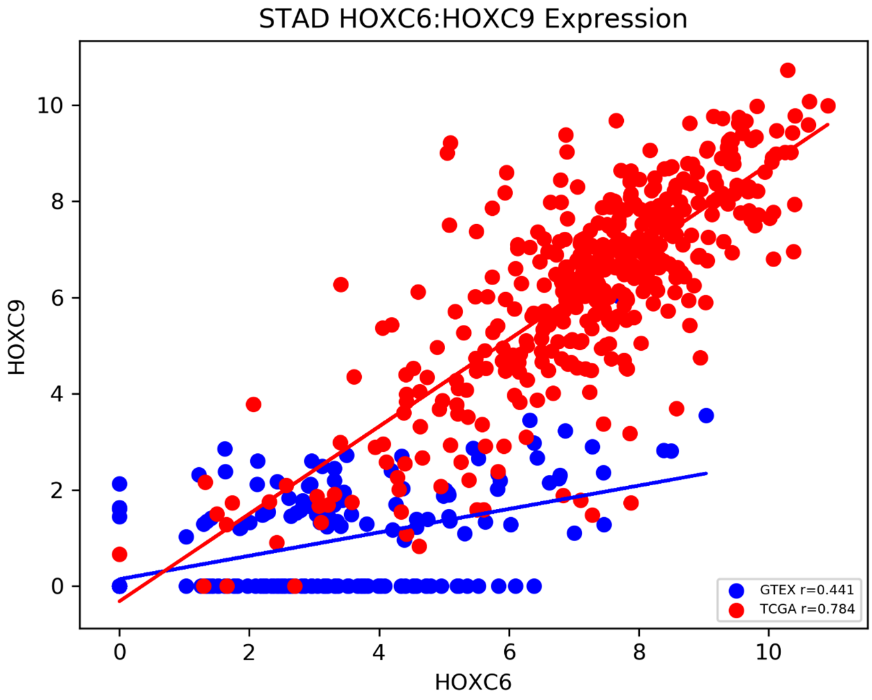

Figure S33. Expression correlation between *HOXC6* and *HOXC9* in samples of healthy stomach tissue and stomach adenocarcinoma (STAD) tumor tissue. The source of expression data of healthy and tumor samples is GTEx and TCGA, respectively. Pierson correlation coefficient (r) indicated in the figure legend.

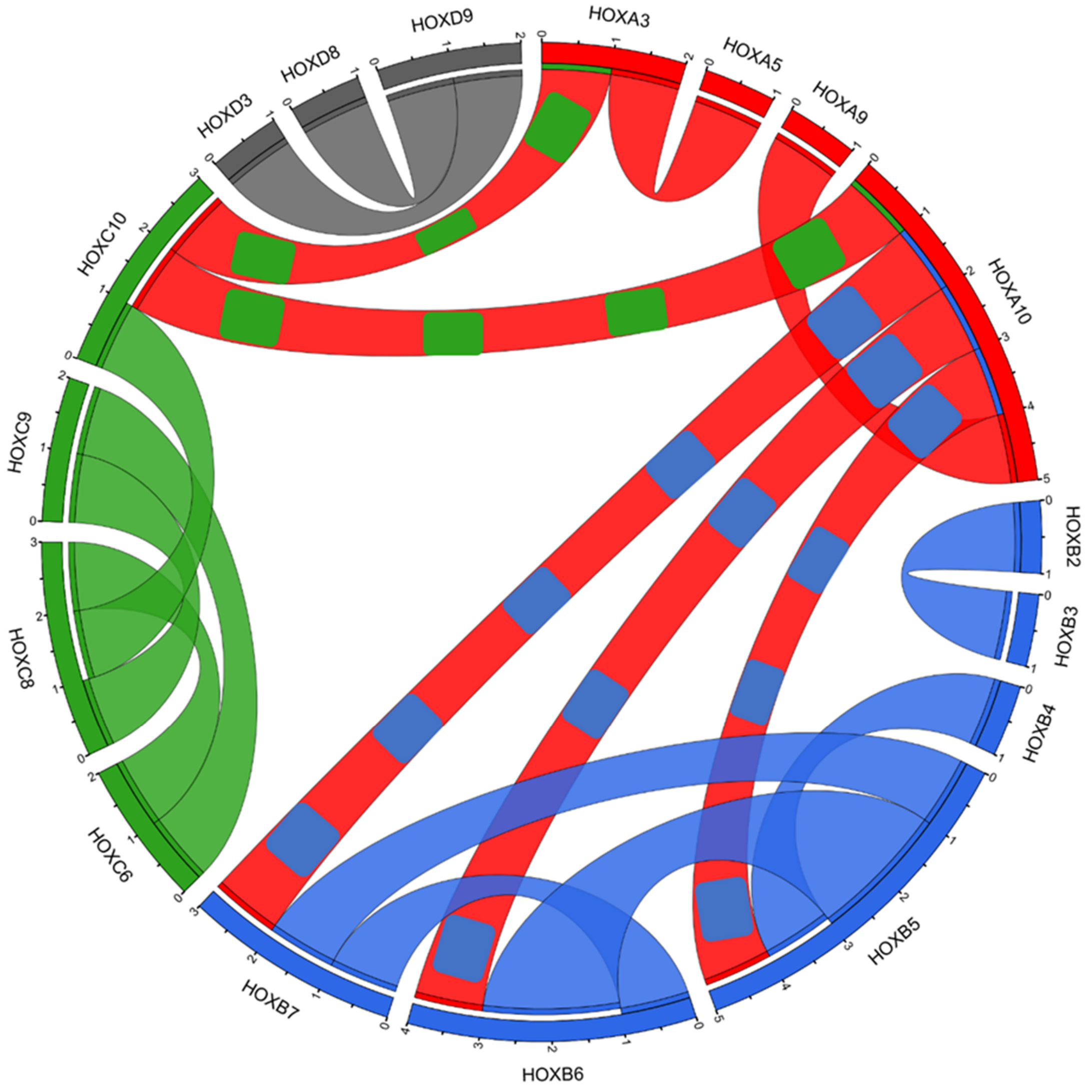

Figure S34. Differentially expressed *HOX* genes pairs that show moderate expression correlation categorized by *HOX* cluster. The chart displays the differentially expressed *HOX* pairs that demonstrate moderate expression correlation (i.e., either r > 0.4 or r < -0.4) in at least one cancer type. The lines color – red, blue, green and grey, represent the different *HOX* clusters A, B, C and D, respectively. The line width represents the number of cancer types in which the specific gene pair demonstrates moderate correlation.

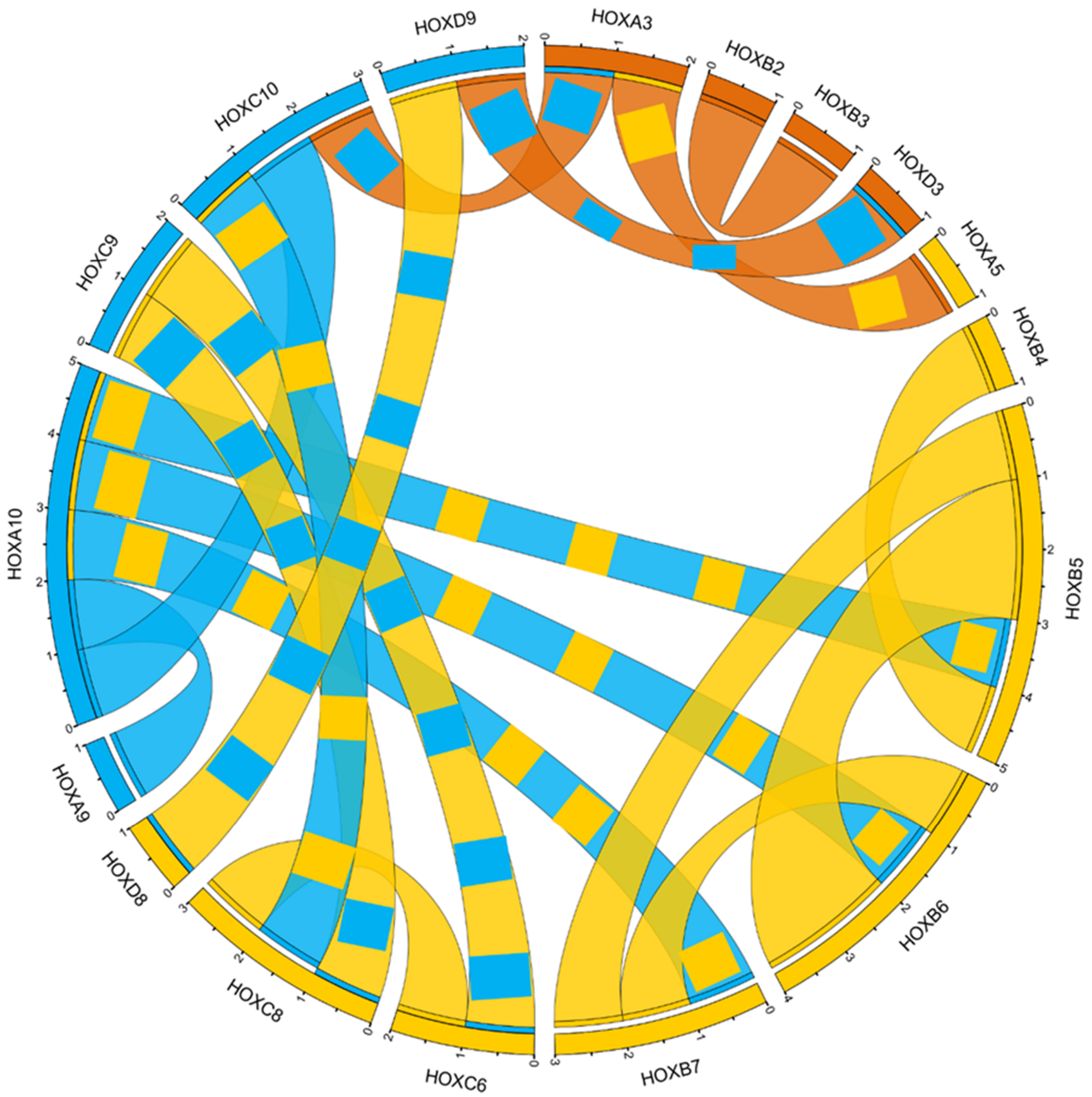

Figure S35. Differentially expressed *HOX* gene pairs that demonstrate moderate expression correlation, categorized by *HOX* group. The chart displays pairs of *HOX* genes that are differentially expressed between healthy and tumor tissue and show moderate (i.e., either r > 0.4 or r < -0.4) expression correlation. The colors: orange, yellow and light blue, represent the different *HOX* groups: anterior, central, posterior, respectively.

Figures S36-S56: Differentially expressed HOX pairs that do not demonstrate expression correlation in healthy tissue (Pearson correlation coefficient < 0.4) and demonstrate high correlation in tumor tissue (Pearson correlation coefficient > 0.7)

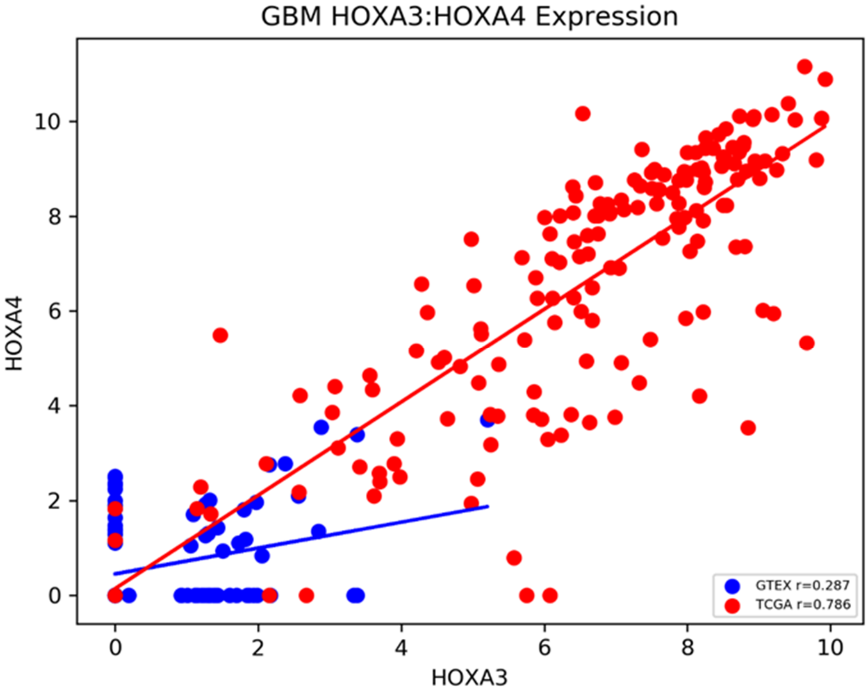

Figure S36. Expression correlation between *HOXA3* and *HOXA4* in samples of in samples of healthy brain tissue and glioblastoma multiforme (GBM) tumor tissue. The source of expression data of healthy and tumor samples is GTEx and TCGA, respectively. Pierson correlation coefficient (r) indicated in the figure legend.

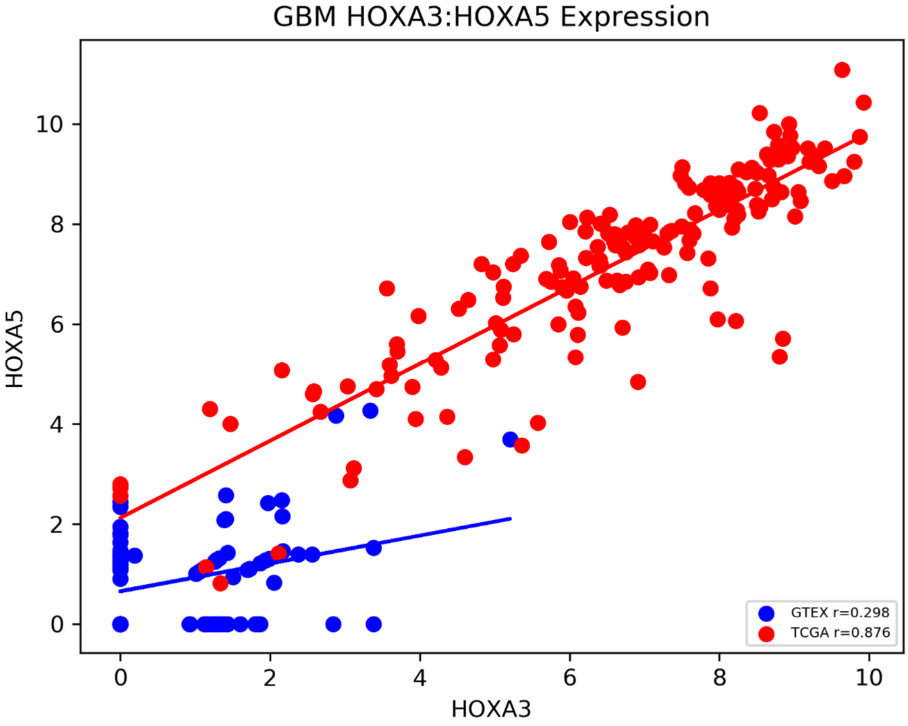

Figure S37. Expression correlation between *HOXA3* and *HOXA5* in samples of in samples of healthy brain tissue and glioblastoma multiforme (GBM) tumor tissue. The source of expression data of healthy and tumor samples is GTEx and TCGA, respectively. Pierson correlation coefficient (r) indicated in the figure legend.

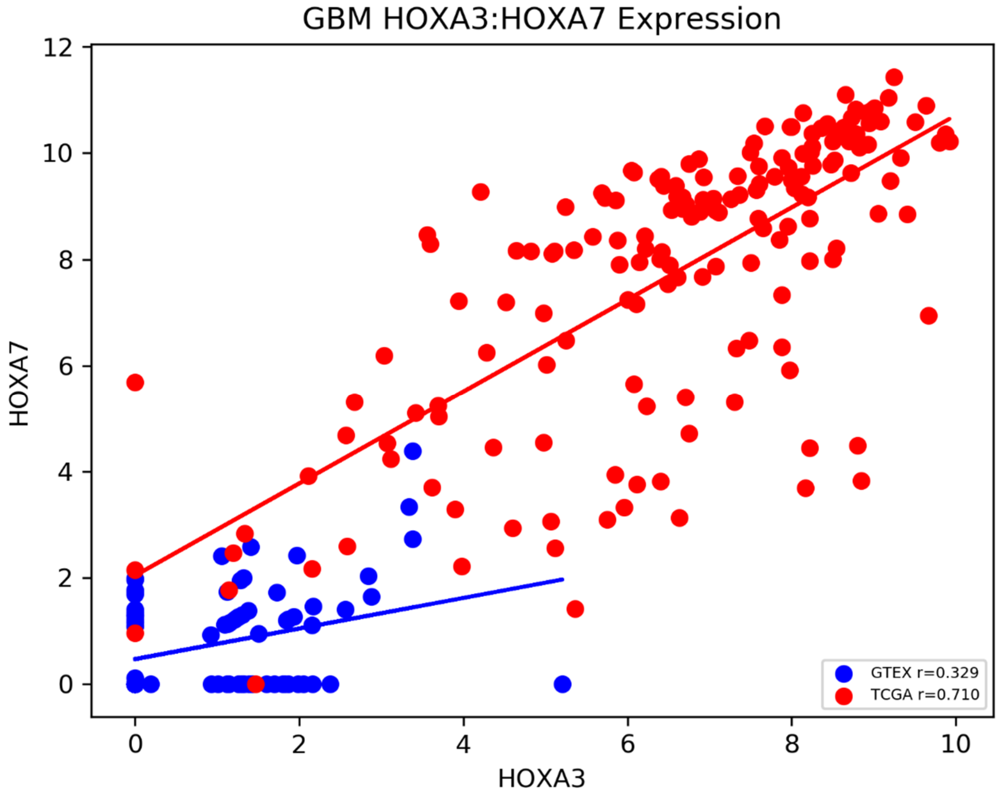

Figure S38. Expression correlation between *HOXA3* and *HOXA7* in samples of in samples of healthy brain tissue and glioblastoma multiforme (GBM) tumor tissue. The source of expression data of healthy and tumor samples is GTEx and TCGA, respectively. Pierson correlation coefficient (r) indicated in the figure legend.

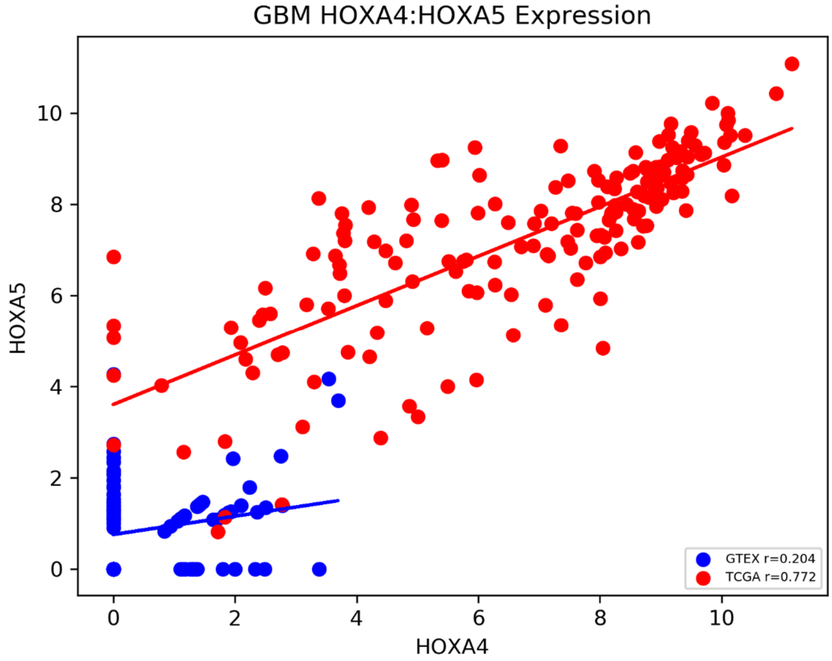

Figure S39. Expression correlation between *HOXA4* and *HOXA5* in samples of in samples of healthy brain tissue and glioblastoma multiforme (GBM) tumor tissue. The source of expression data of healthy and tumor samples is GTEx and TCGA, respectively. Pierson correlation coefficient (r) indicated in the figure legend.

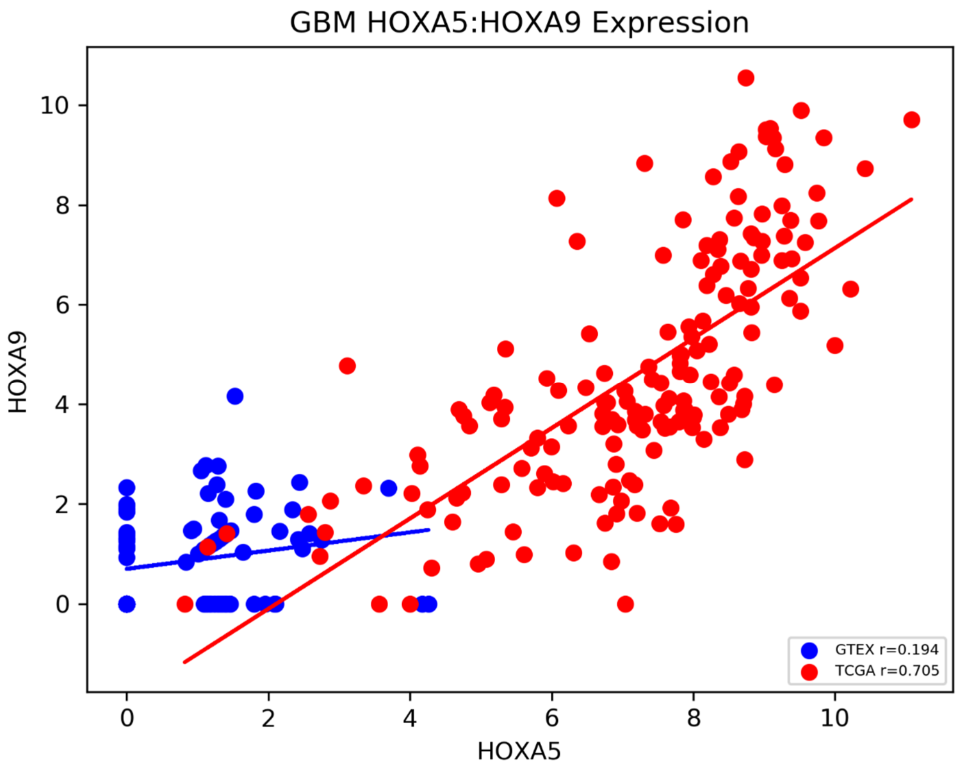

Figure S40. Expression correlation between *HOXA5* and *HOXA9* in samples of in samples of healthy brain tissue and glioblastoma multiforme (GBM) tumor tissue. The source of expression data of healthy and tumor samples is GTEx and TCGA, respectively. Pierson correlation coefficient (r) indicated in the figure legend.

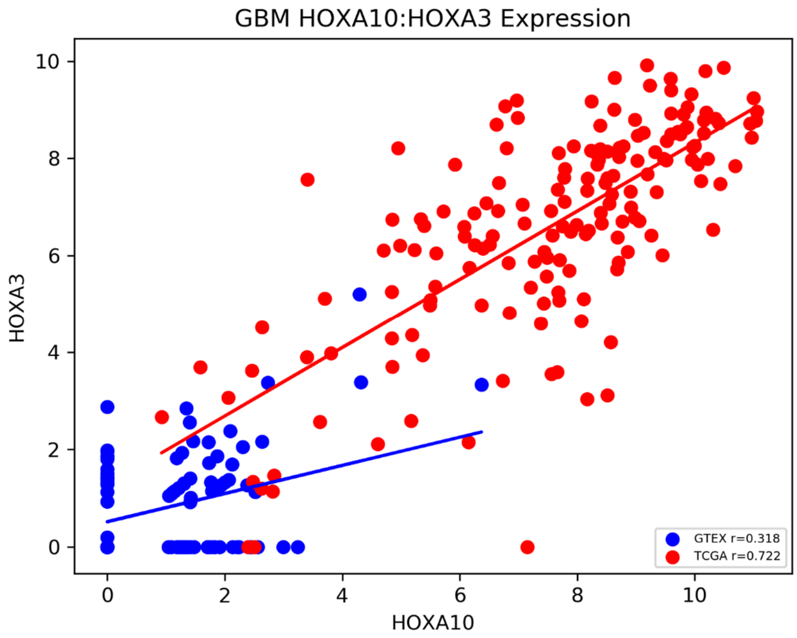

Figure S41. Expression correlation between *HOXA10* and *HOXA3* in samples of in samples of healthy brain tissue and glioblastoma multiforme (GBM) tumor tissue. The source of expression data of healthy and tumor samples is GTEx and TCGA, respectively. Pierson correlation coefficient (r) indicated in the figure legend.

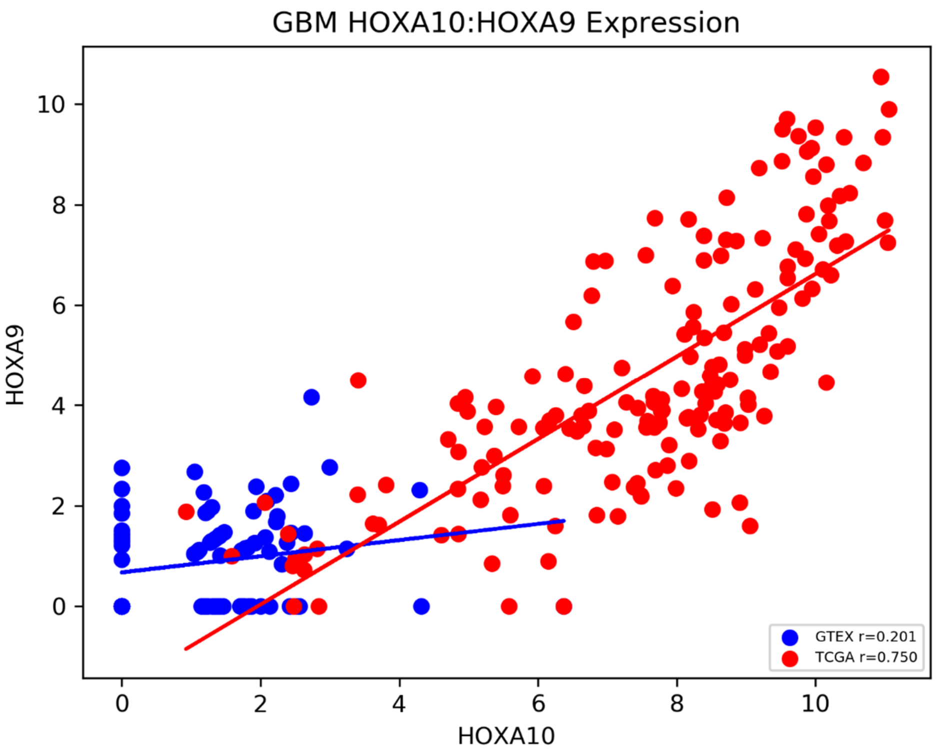

Figure S42. Expression correlation between *HOXA10* and *HOXA9* in samples of in samples of healthy brain tissue and glioblastoma multiforme (GBM) tumor tissue. The source of expression data of healthy and tumor samples is GTEx and TCGA, respectively. Pierson correlation coefficient (r) indicated in the figure legend.

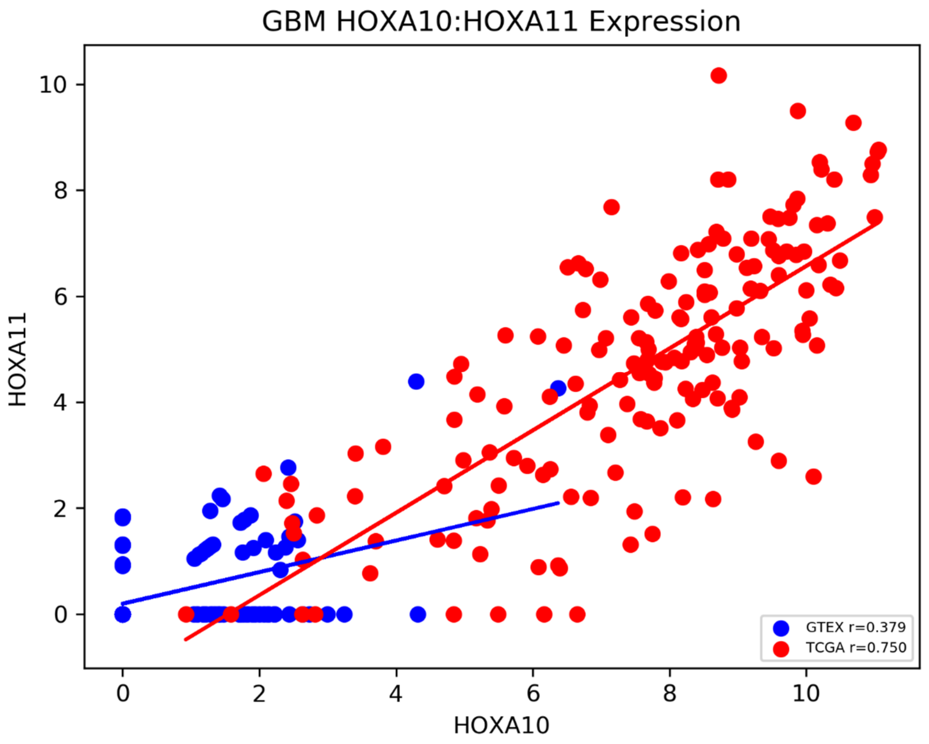

Figure S43. Expression correlation between *HOXA10* and *HOXA11* in samples of in samples of healthy brain tissue and glioblastoma multiforme (GBM) tumor tissue. The source of expression data of healthy and tumor samples is GTEx and TCGA, respectively. Pierson correlation coefficient (r) indicated in the figure legend.

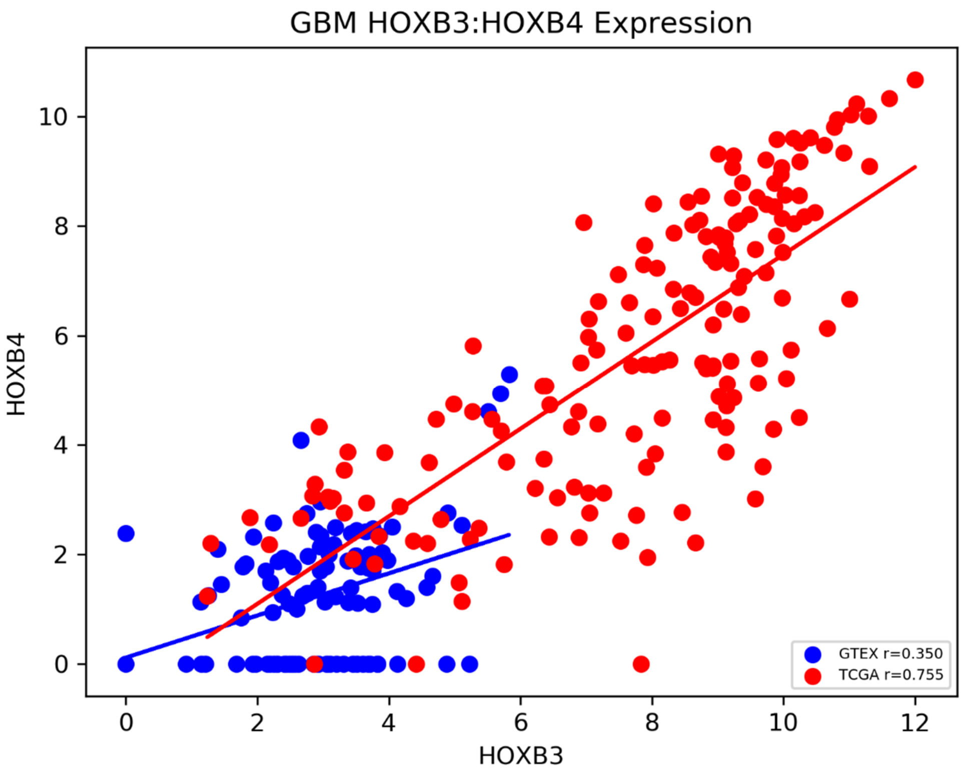

Figure S44. Expression correlation between *HOXB3* and *HOXB4* in samples of in samples of healthy brain tissue and glioblastoma multiforme (GBM) tumor tissue. The source of expression data of healthy and tumor samples is GTEx and TCGA, respectively. Pierson correlation coefficient (r) indicated in the figure legend.

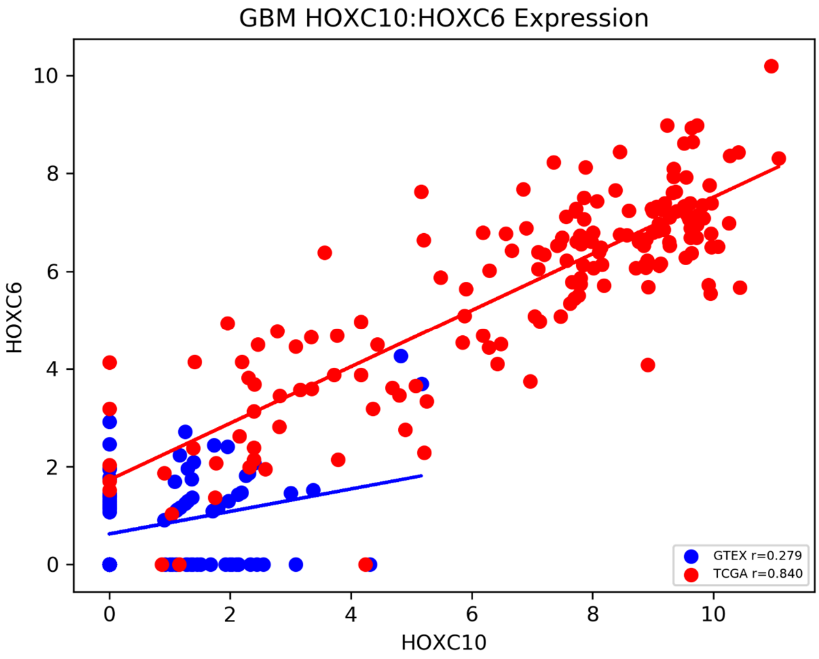

Figure S45. Expression correlation between *HOXC10* and *HOXC6* in samples of in samples of healthy brain tissue and glioblastoma multiforme (GBM) tumor tissue. The source of expression data of healthy and tumor samples is GTEx and TCGA, respectively. Pierson correlation coefficient (r) indicated in the figure legend.

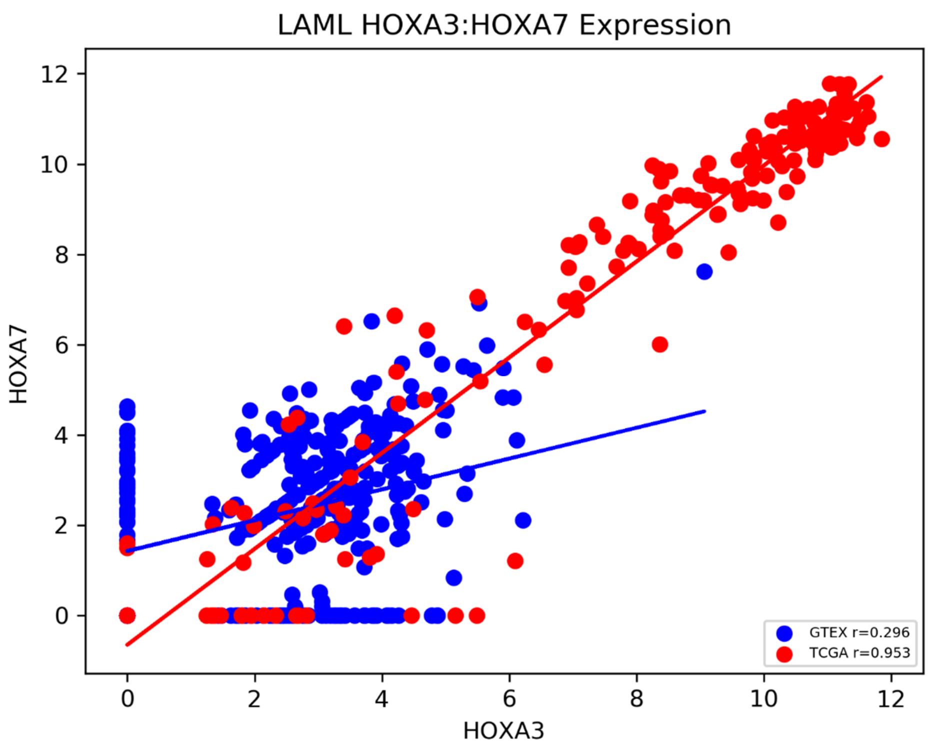

Figure S46. Expression correlation between *HOXA3* and *HOXA7* in samples of healthy blood tissue and acute meloid leukemia (LAML) tumor tissue. The source of expression data of healthy and tumor samples is GTEx and TCGA, respectively. Pierson correlation coefficient (r) indicated in the figure legend.

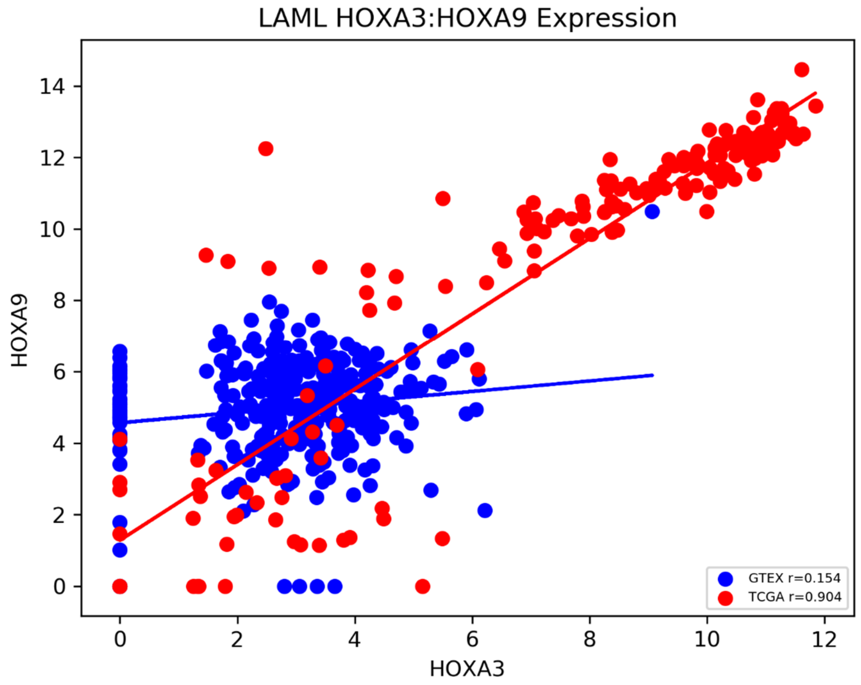

Figure S47. Expression correlation between *HOXA3* and *HOXA9* in samples of healthy blood tissue and acute meloid leukemia (LAML) tumor tissue. The source of expression data of healthy and tumor samples is GTEx and TCGA, respectively. Pierson correlation coefficient (r) indicated in the figure legend.

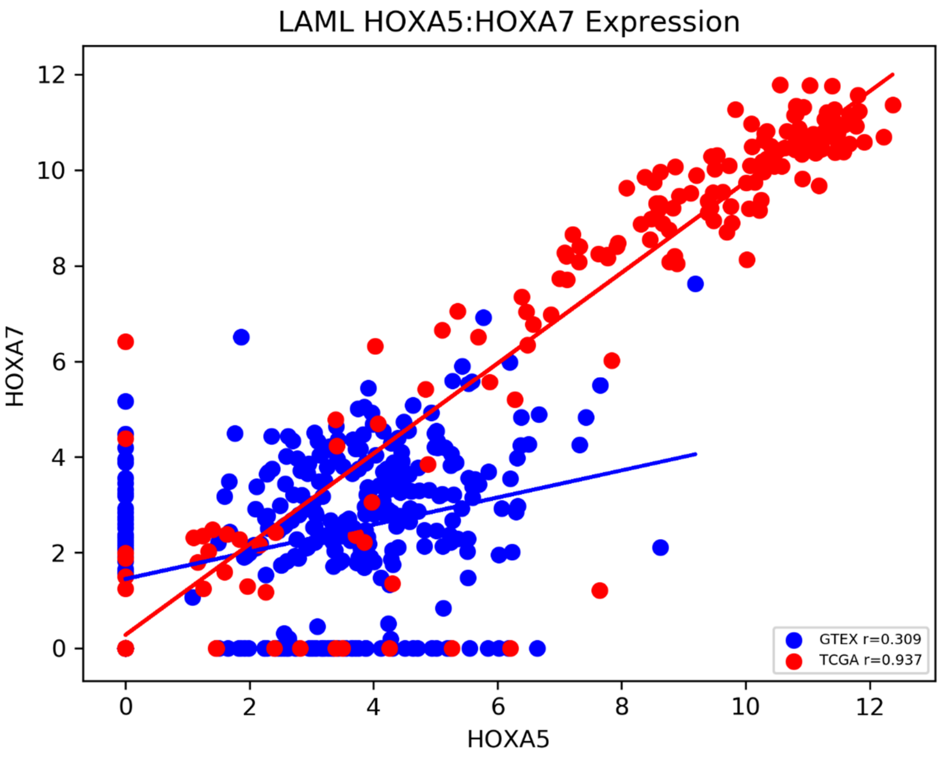

Figure S48. Expression correlation between *HOXA5* and *HOXA7* in samples of healthy blood tissue and acute meloid leukemia (LAML) tumor tissue. The source of expression data of healthy and tumor samples is GTEx and TCGA, respectively. Pierson correlation coefficient (r) indicated in the figure legend.

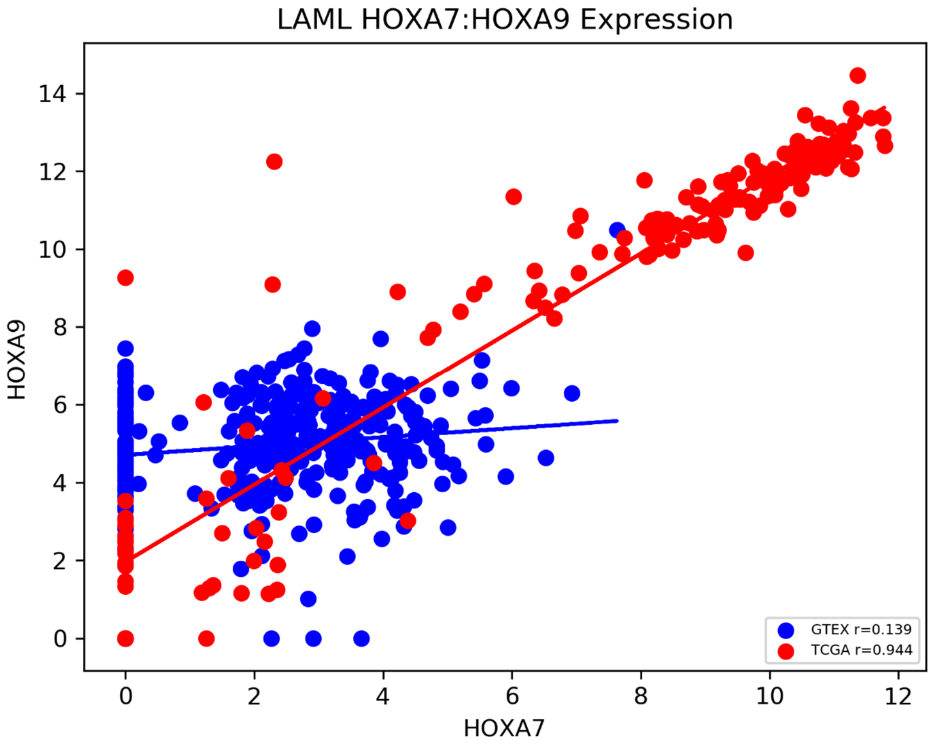

Figure S49. Expression correlation between *HOXA7* and *HOXA9* in samples of healthy blood tissue and acute meloid leukemia (LAML) tumor tissue. The source of expression data of healthy and tumor samples is GTEx and TCGA, respectively. Pierson correlation coefficient (r) indicated in the figure legend.

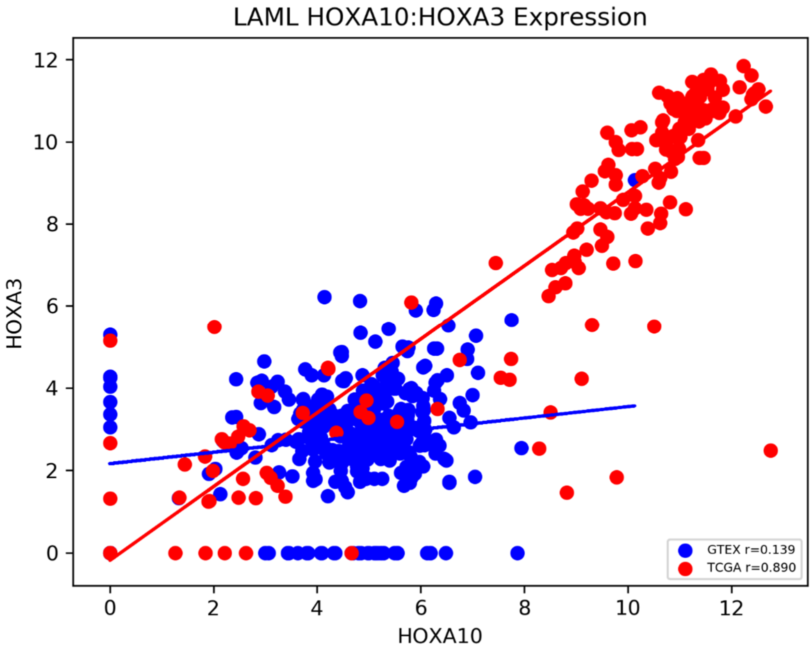

Figure S50. Expression correlation between *HOXA10* and *HOXA3* in samples of healthy blood tissue and acute meloid leukemia (LAML) tumor tissue. The source of expression data of healthy and tumor samples is GTEx and TCGA, respectively. Pierson correlation coefficient (r) indicated in the figure legend.

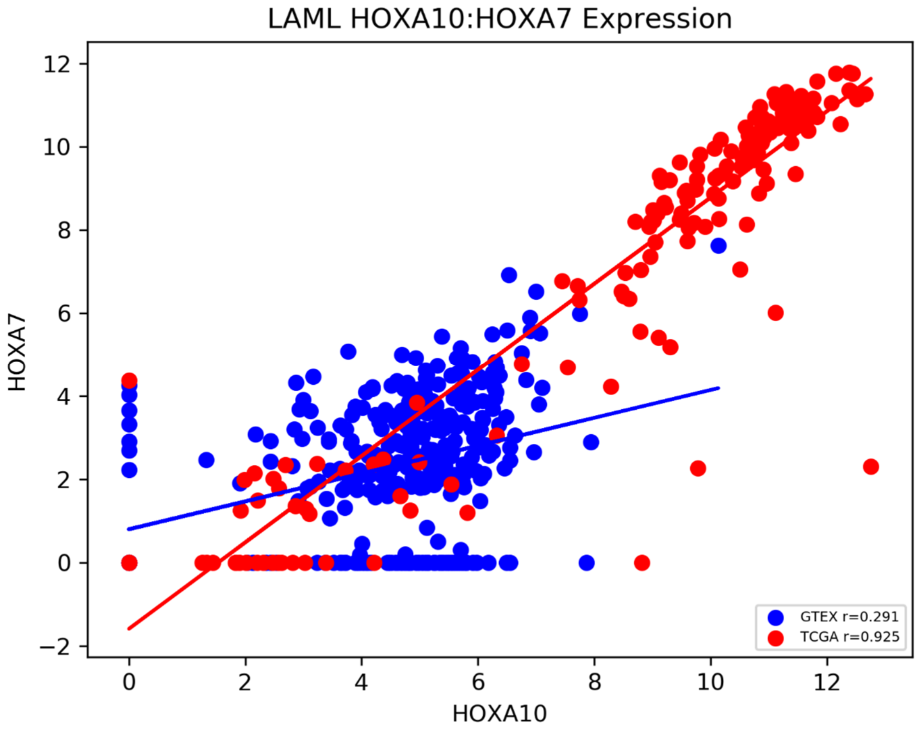

Figure S51. Expression correlation between *HOXA10* and *HOXA7* in samples of healthy blood tissue and acute meloid leukemia (LAML) tumor tissue. The source of expression data of healthy and tumor samples is GTEx and TCGA, respectively. Pierson correlation coefficient (r) indicated in the figure legend.

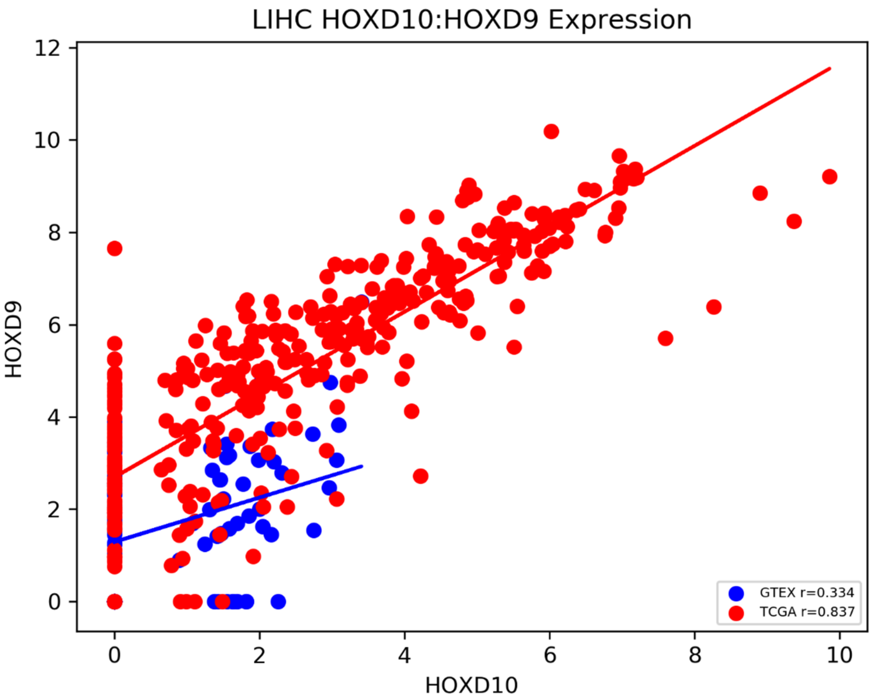

Figure S52. Expression correlation between *HOXD10* and *HOXD9* in samples of healthy liver tissue and liver hepatocellular carcinoma (LIHC) tumor tissue. The source of expression data of healthy and tumor samples is GTEx and TCGA, respectively. Pierson correlation coefficient (r) indicated in the figure legend.

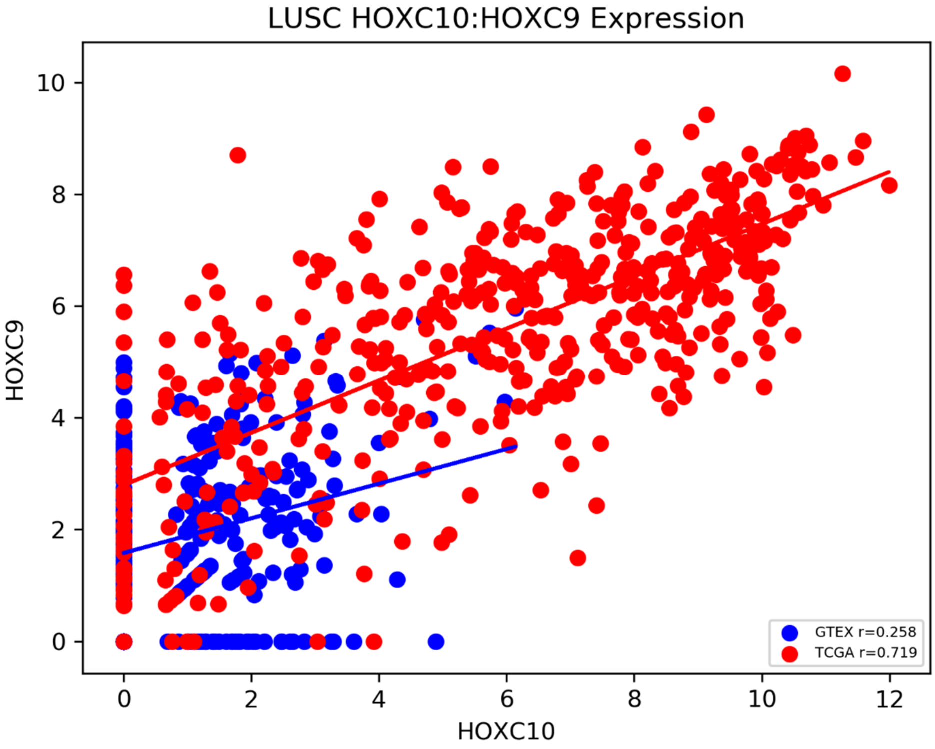

Figure S53. Expression correlation between *HOXC10* and *HOXC9* in samples of healthy lung tissue and lung squamous cell carcinoma (LUSC) tumor tissue. The source of expression data of healthy and tumor samples is GTEx and TCGA, respectively. Pierson correlation coefficient (r) indicated in the figure legend.

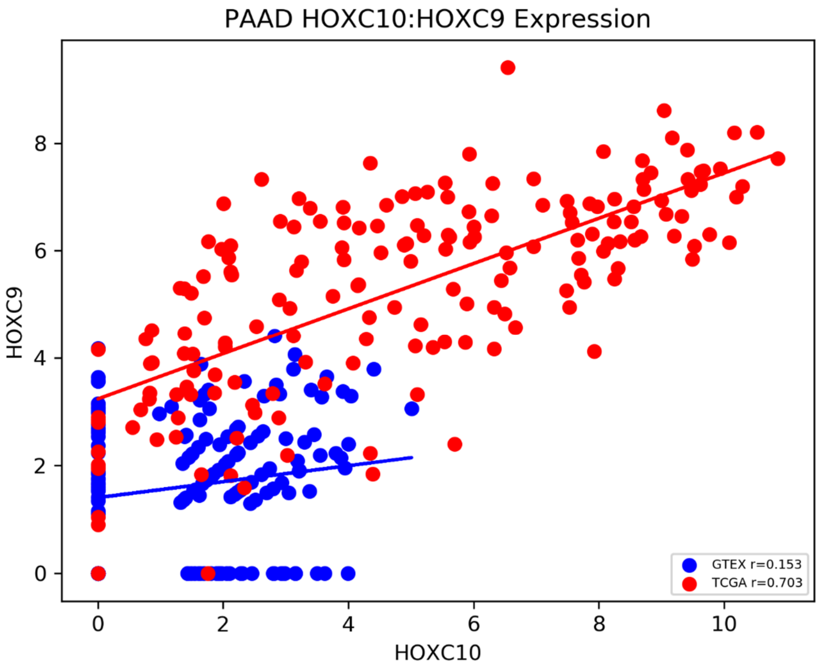

Figure S54. Expression correlation between *HOXC10* and *HOXC9* in samples of healthy pancreas tissue and pancreatic adenocarcinoma (PAAD) tumor tissue. The source of expression data of healthy and tumor samples is GTEx and TCGA, respectively. Pierson correlation coefficient (r) indicated in the figure legend.

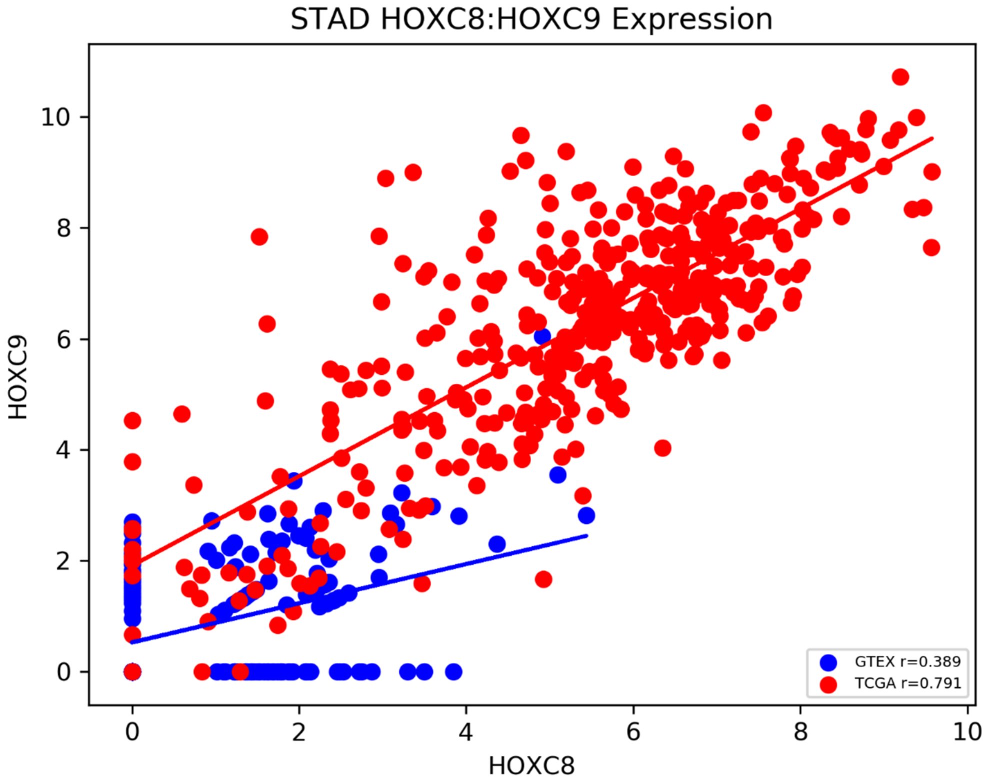

Figure S55. Expression correlation between *HOXC8* and *HOXC9* in samples of healthy stomach tissue and stomach adenocarcinoma (STAD) tumor tissue. The source of expression data of healthy and tumor samples is GTEx and TCGA, respectively. Pierson correlation coefficient (r) indicated in the figure legend.

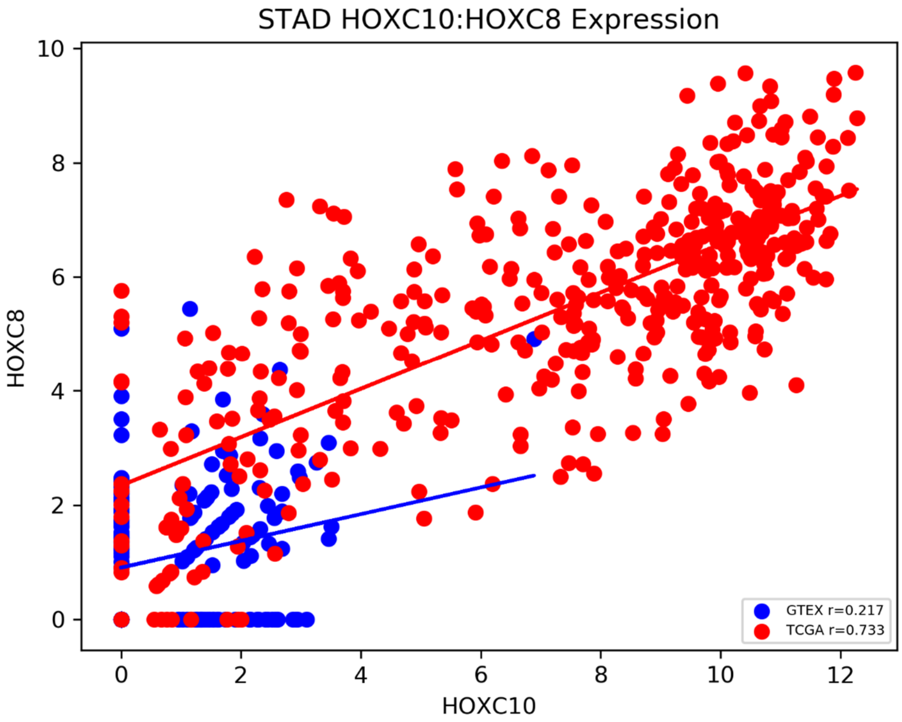

Figure S56. Expression correlation between *HOXC10* and *HOXC8* in samples of healthy stomach tissue and stomach adenocarcinoma (STAD) tumor tissue. The source of expression data of healthy and tumor samples is GTEx and TCGA, respectively. Pierson correlation coefficient (r) indicated in the figure legend.

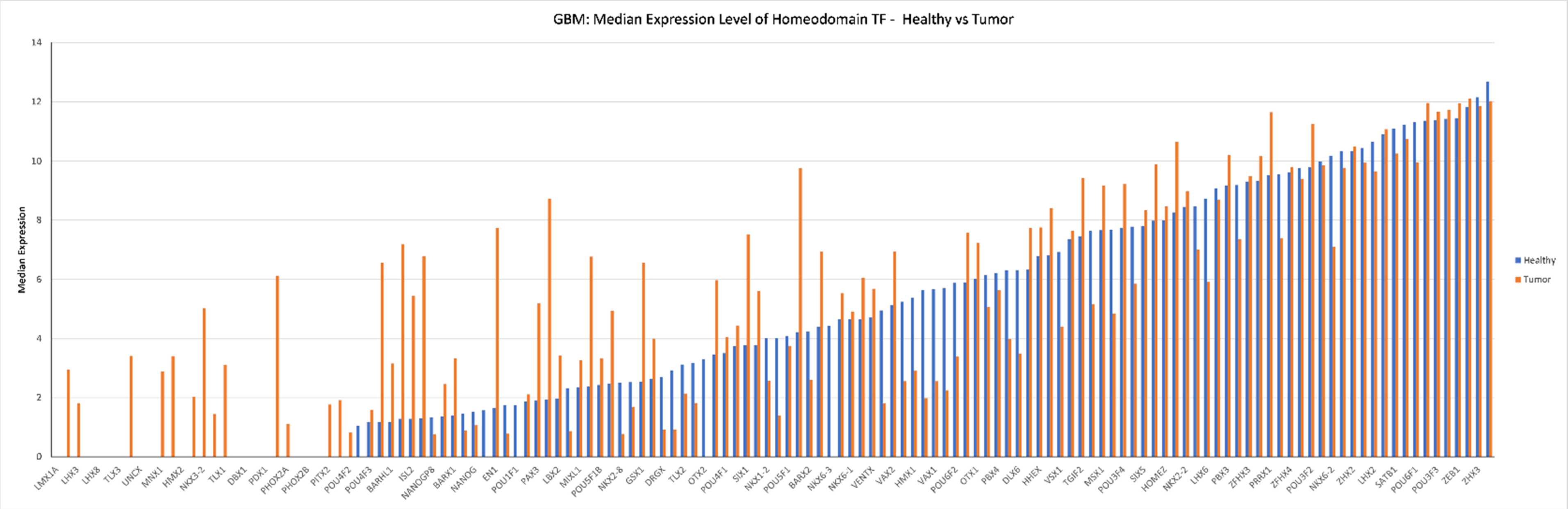

Figure S57. Median expression level of Homeodomain transcription factors (not including *HOX* genes) in healthy brain tissue and GBM tumor tissue.

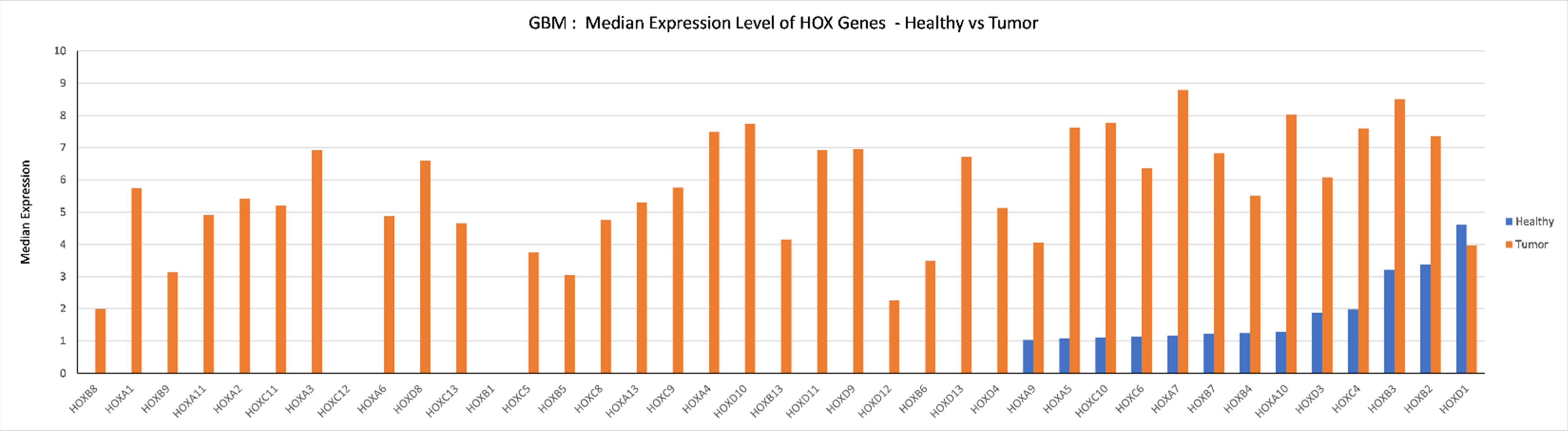

Figure S58. Median expression level of *HOX* genes in healthy brain tissue and GBM tumor tissue.

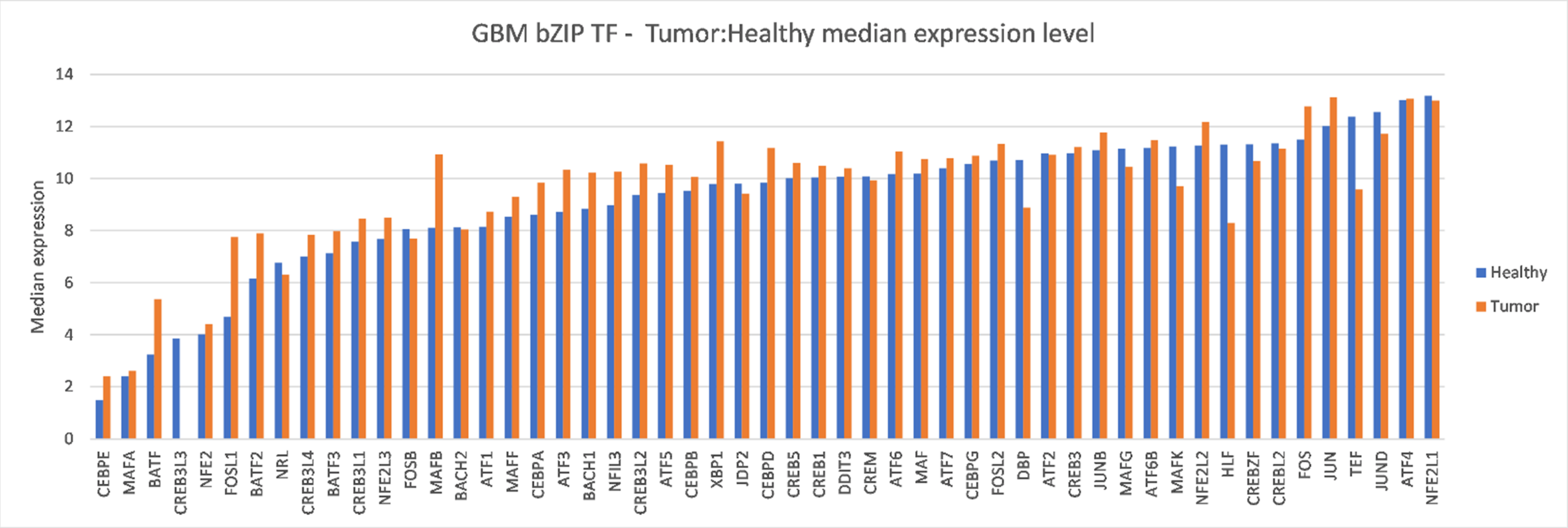

Figure S59. Median expression level of bZIP transcription factors in healthy brain tissue and GBM tumor tissue.

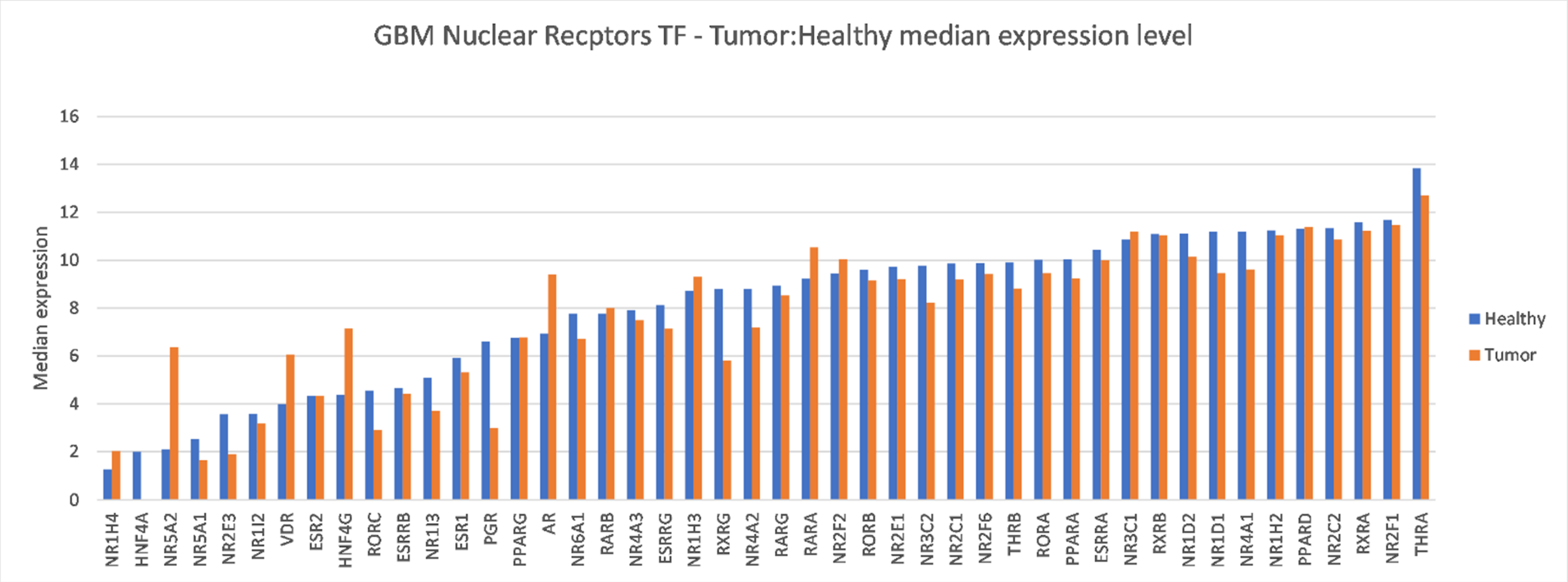

Figure S60. Median expression level of Nuclear Receptors transcription factors in healthy brain tissue and GBM tumor tissue.

Table S3. Lowly expressed *HOX* genes that undergo a significant expression increase in cancer[1].

Gene Name	Cancer Type								
	BRCA	ESCA	GBM	LAML	LGG	LIHC	LUSC	PAAD	STAD
HOXA1			5.7						
HOXA2			5.4						
HOXA3			6.9						
HOXA4			7.5						
HOXA6			4.9	7.5					
HOXA7			8.2						
HOXA11		6.7	4.9				5.2	4.3	7.1
HOXA13		7.6	5.3			6.8	6.1	6.6	9.4
HOXB5			3.7	6.2					
HOXB6			3.8						
HOXB7			7.0		3.7				
HOXB8		4.9							
HOXB9		8.0	**3.3***					7.4	
HOXB13			4.2				5.7	4.3	8.2
HOXC5			3.9					4.5	
HOXC8			4.8						
HOXC9			5.8						6.6
HOXC10			7.8						
HOXC11		7.3	5.2				5.4	5.4	7.4
HOXC12	3.6	3.9							
HOXC13			5.0				7.2		3.4
HOXD4			5.1						
HOXD8			6.6		**5.1***				
HOXD9			7.0						
HOXD10			7.7						
HOXD11			6.9				7.0		
HOXD13		4.2	6.7				7.1		

[1]*HOX* genes that have low expression in healthy tissue, (less than 1 (log2(x+1) transformed RSEM normalized count)), their expression change in tumor tissue is higher than 3-fold and their expression in the tumor tissue is greater than 1(log2(x+1) transformed RSEM normalized count). For each *HOX* gene that meets the above criteria, its median expression level in the related tumor tissue is provided. The cells marked in *bold highlight the genes whose KM graph showed significant correlation between gene expression level and patients' survival (Supplemental Table S4).

Table S4. Differentially expressed *HOX* genes whose KM graph show significant correlation between gene expression and patient's survival

Cancer	Gene	KM
Brain LGG	*HOXA1*	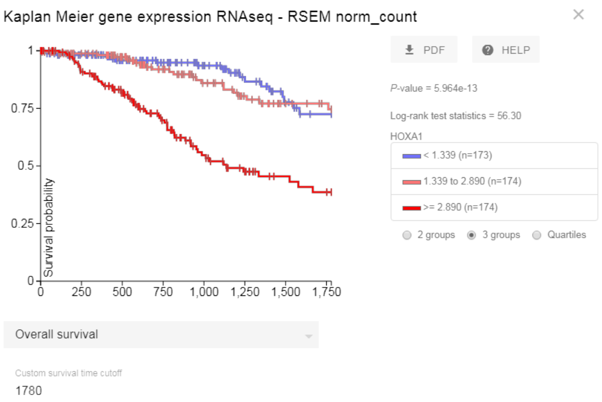

Brain LGG *HOXA4*

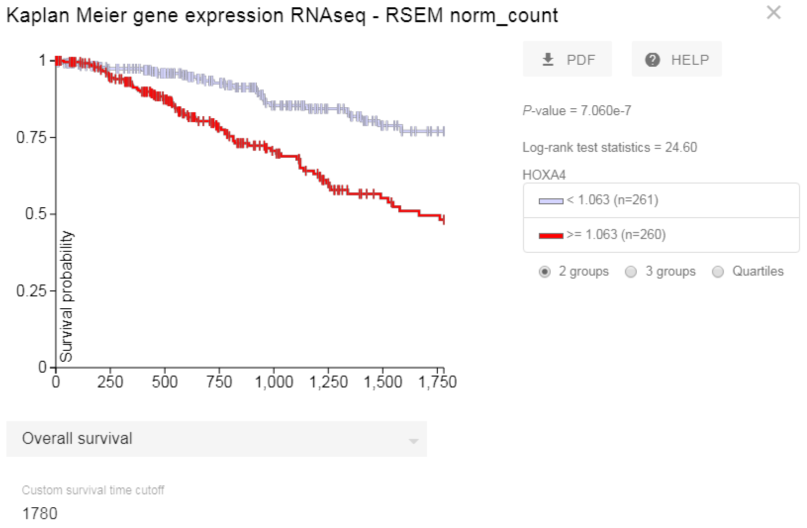

Brain LGG *HOXA7*

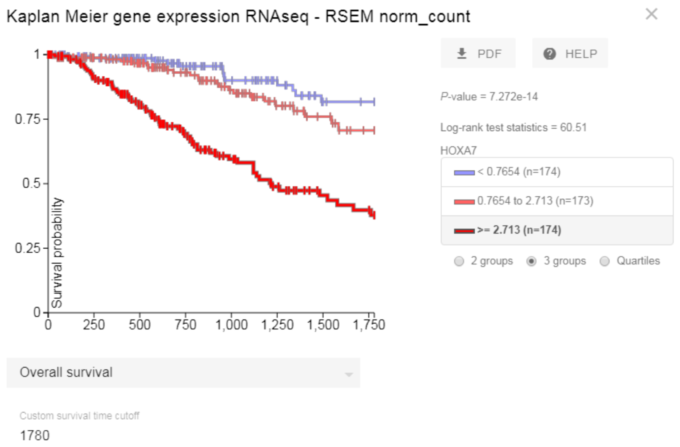

Brain LGG *HOXA11*

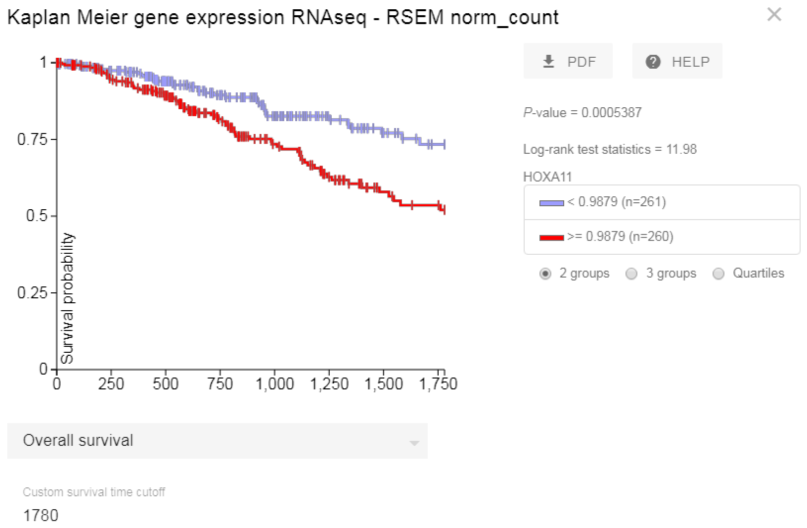

Brain LGG *HOXB13*

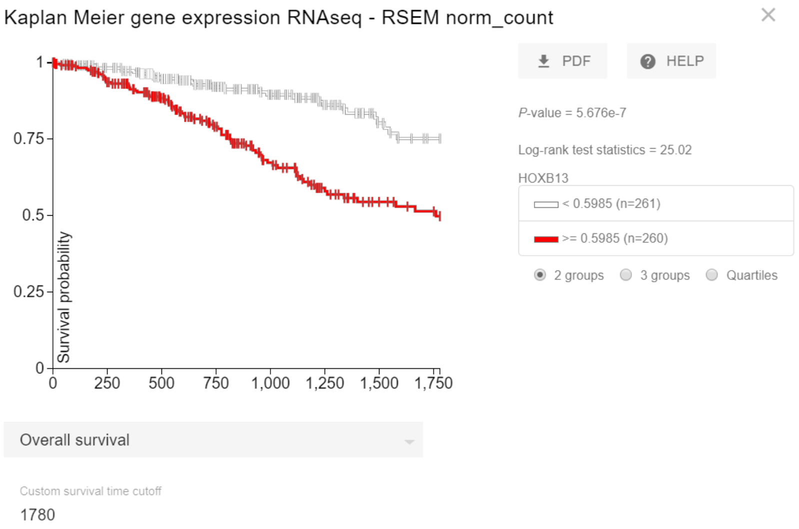

Brain LGG *HOXC4*

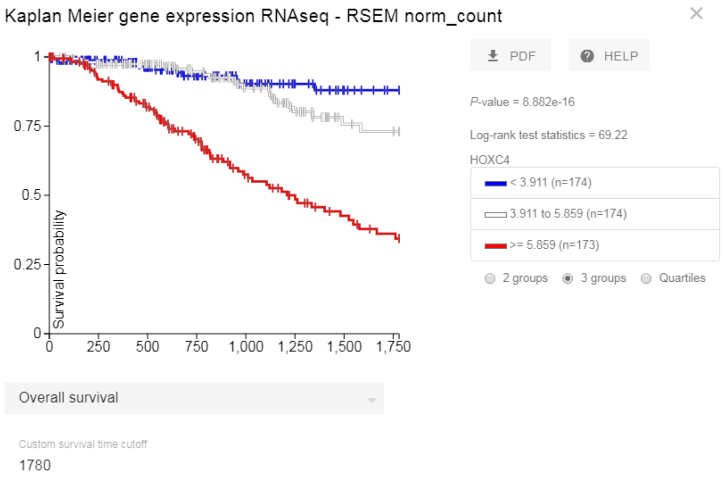

Brain LGG *HOXD3*

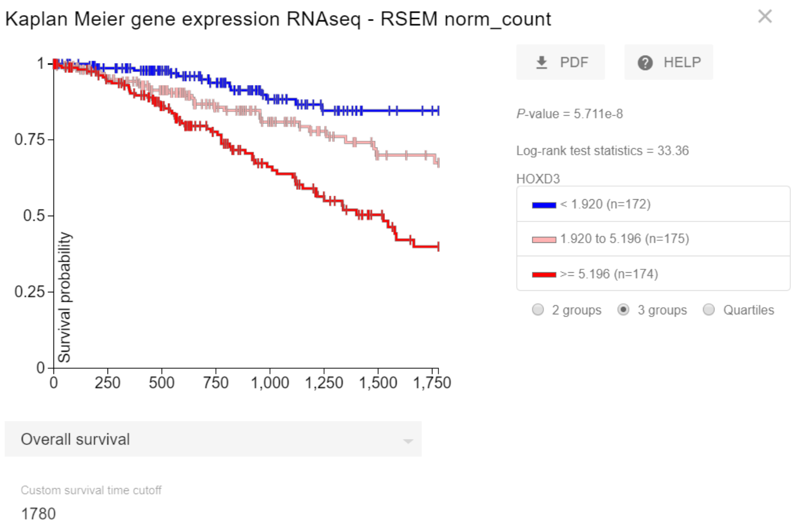

Brain LGG *HOXD4*

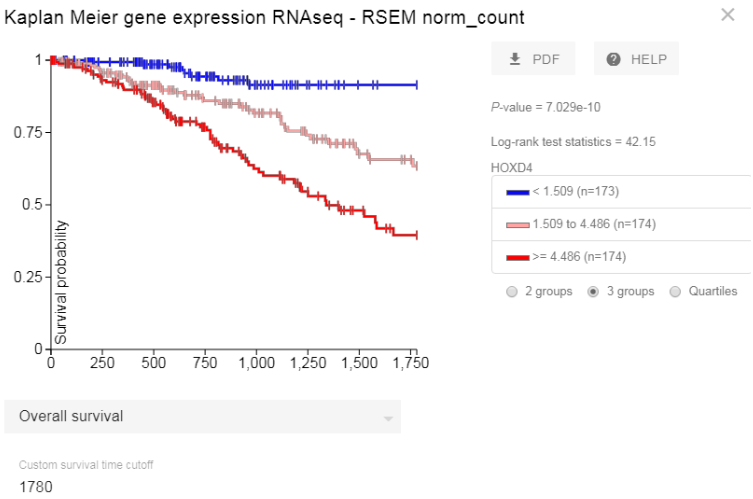

Brain LGG *HOXD8*

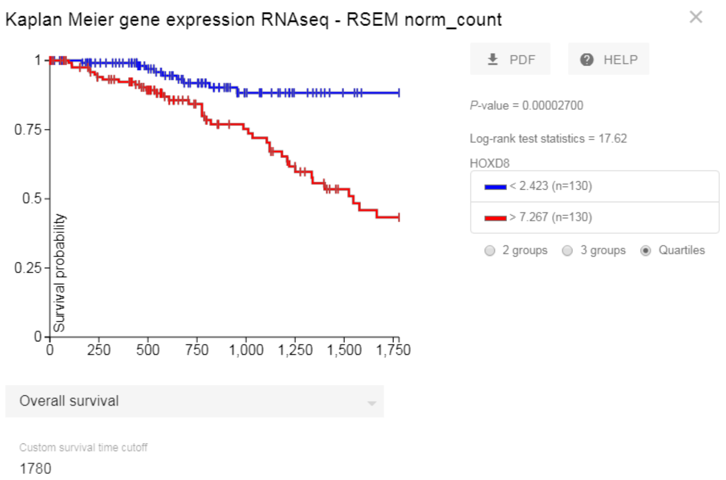

Brain LGG *HOXD9*

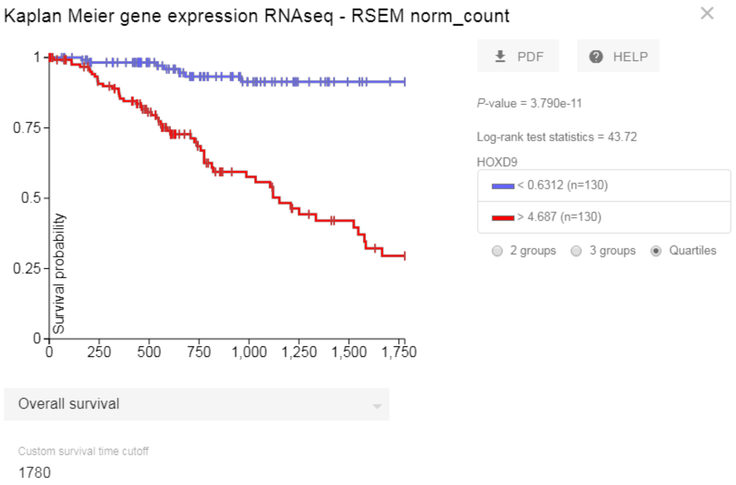

Brain LGG *HOXD10*

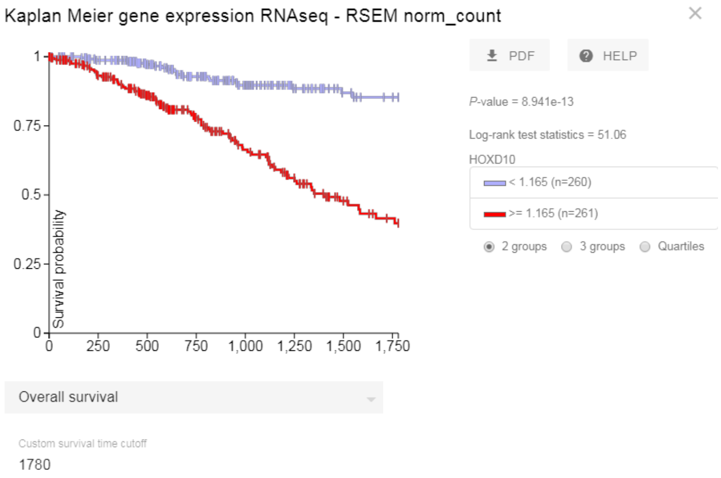

Brain GBM *HOXB2*

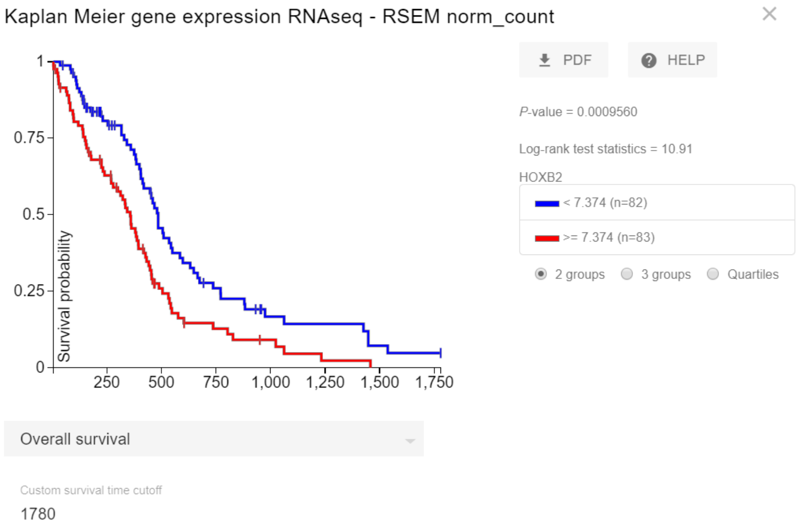

Brain GBM *HOXB9*

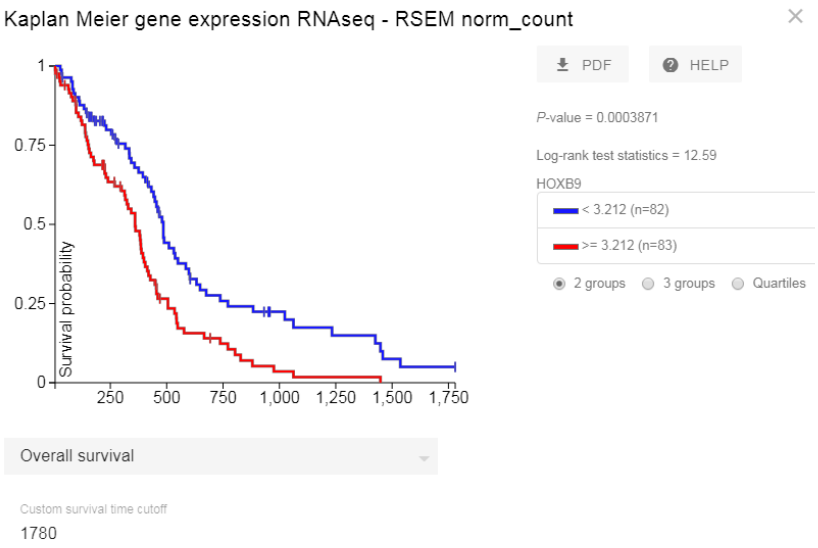

Leukemia LAML *HOXA7*

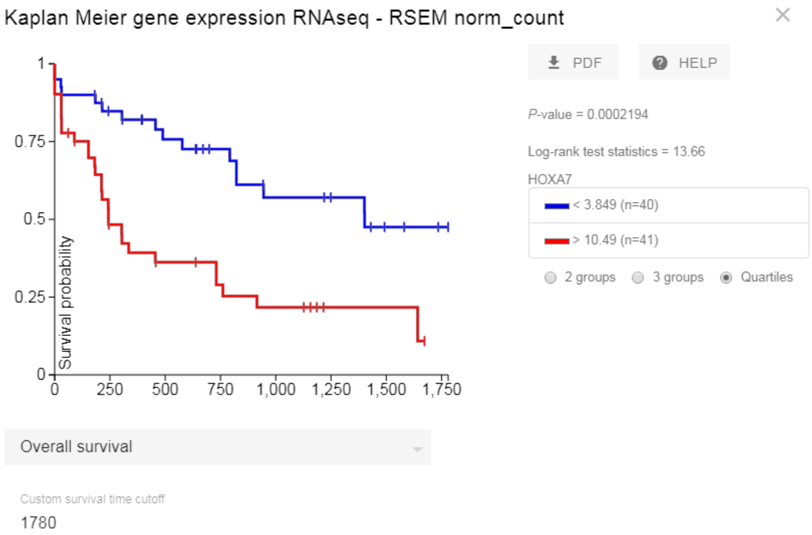

Table S8. Differential expression status* of prognostic markers previously proposed in literature.

Cancer Type	Marker Gene name	Reference	Is differentially expressed	Is KM significant
ESCA	*EGFR*	[1]	No	No
ESCA	*ERBB2*	[2]	No	No
GBM	*ABCC3*	[3]	Yes	Yes
GBM	*CD44*	[3]	No	No
GBM	*EGFR*	[3,4]	No	No
GBM	*PDGFRA*	[4]	No	No
GBM	*TNFRSF1A*	[3]	No	Yes
LAML	*BAALC*	[5]	No	No
LAML	*CDX2*	[5]	Yes	No
LAML	*CD33*	[5]	No	No
LAML	*ERG*	[5]	No	No
LAML	*KIT*	[5]	Yes	Yes
LAML	*MN1*	[5]	No	No
LAML	*PRAME*	[5]	No	No
LAML	*WT1*	[5]	Yes	Yes
LGG	*CD44*	[6]	No	Yes
LIHC	*BIRC5*	[7]	No	Yes
LIHC	*CDH1*	[8]	No	Yes
LIHC	*CEACAM5*	[9]	Yes	No
LIHC	*ECM2*	[8]	No	Yes
LIHC	*MMP9*	[8]	No	Yes
LIHC	*MUC16*	[9]	No	No
LIHC	*NAT10*	[7]	No	Yes
LIHC	*VIM*	[8]	No	No
LUSC	*BIRC5*	[10]	No	No
PAAD	*SLC29A1*	[11]	No	No
PAAD	*MIR155*	[11]	No	No
PAAD	*MIR196A1*	[11]	No	No
PAAD	*MIR196A2*	[11]	No	No
PAAD	*MIR21*	[11]	No	No
PAAD	*MIR210*	[11]	No	No
PAAD	*MIR217*	[11]	No	No
STAD	*CEACAM5*	[12]	Yes	No

*2-fold or more in our study.

Table S9. Kaplan-Meier (KM) survival graphs of genes used as prognostic markers.

Cancer Type	Marker Gene Name	KM graph
GBM	*ABCC3*	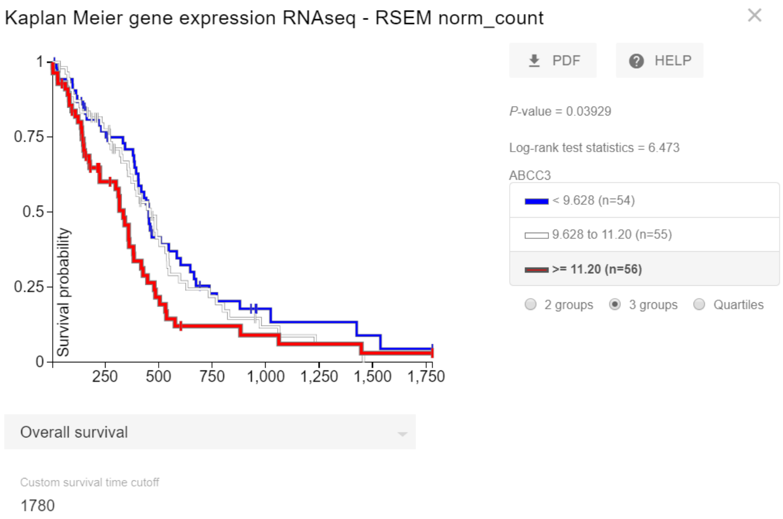

GBM *TNFRSF1A*

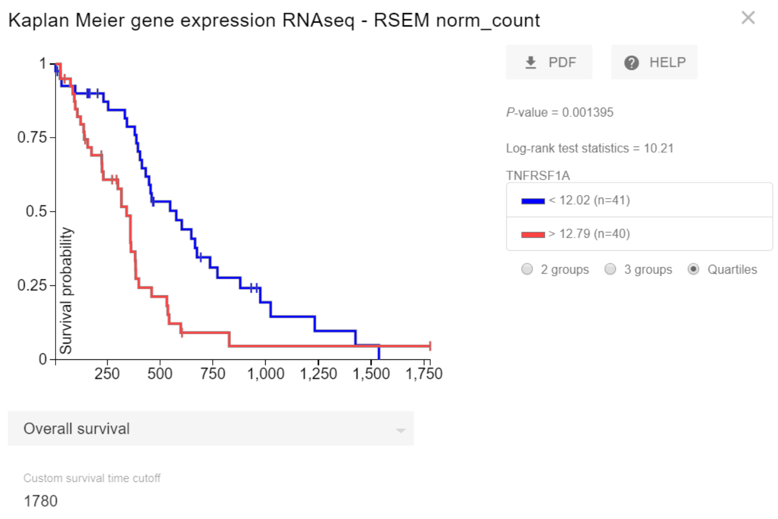

LAML *KIT*

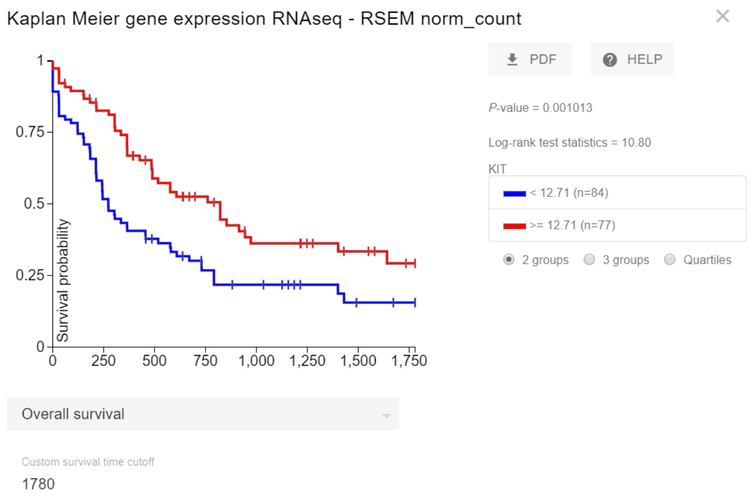

LAML *WT1*

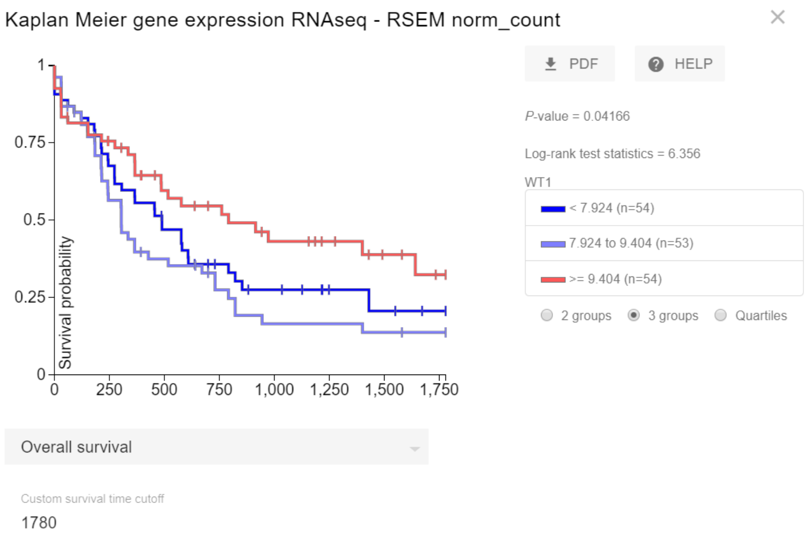

LGG *CD44*

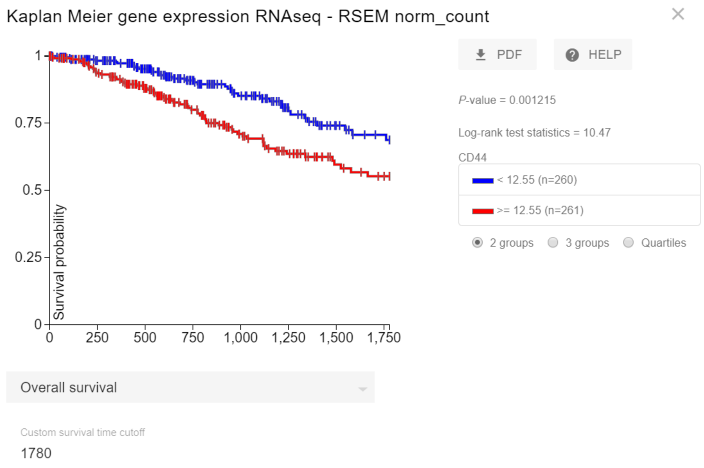

LIHC	*BIRC5*	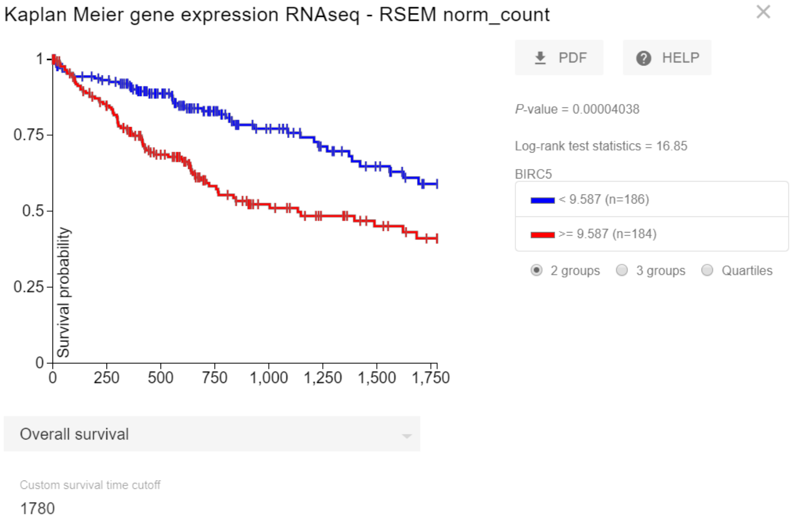
LIHC	*CDH1*	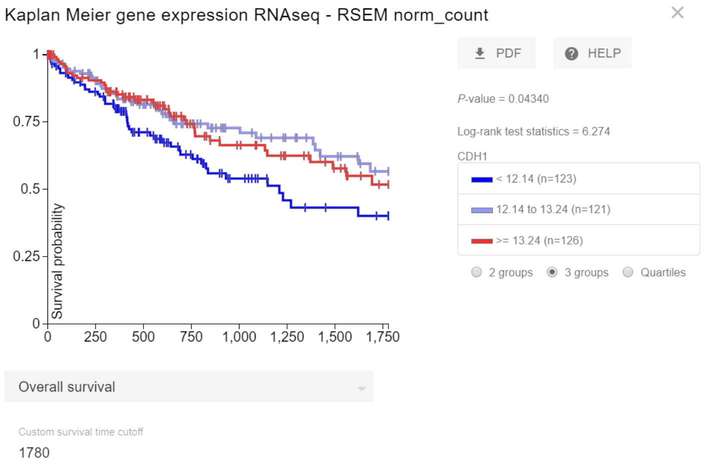

LIHC	*ECM2*	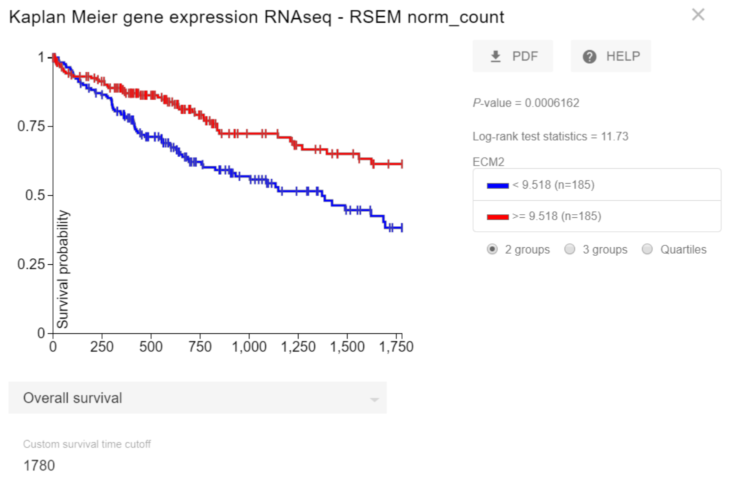
LIHC	*MMP9*	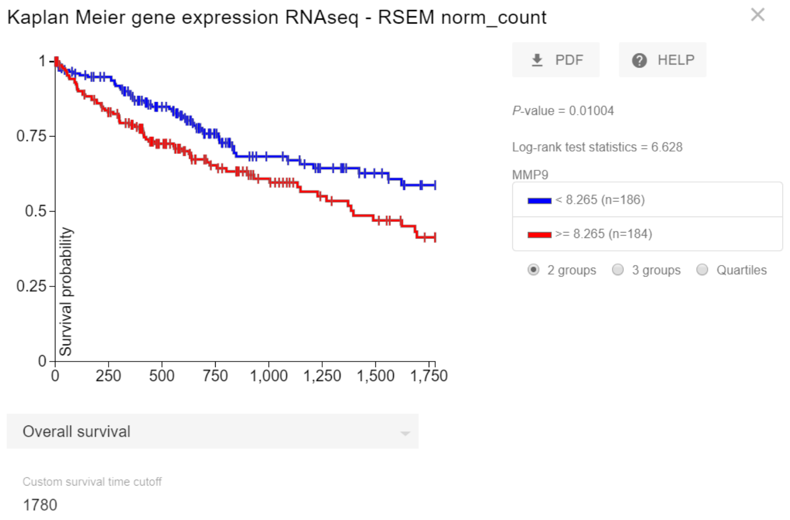

LIHC *NAT10*

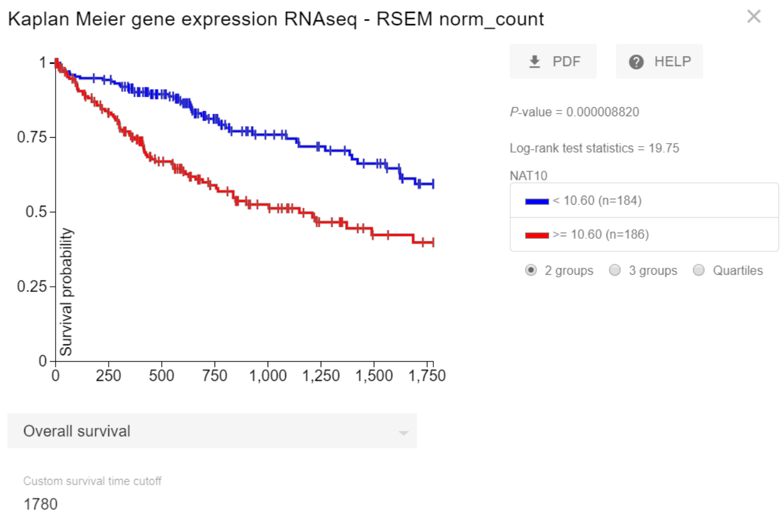
Kaplan Meier gene expression RNAseq - RSEM norm_count
PDF
HELP
P-value = 0.000008820
Log-rank test statistics = 19.75
NAT10
< 10.60 (n=184)
>= 10.60 (n=186)
2 groups
3 groups
Quartiles
Survival probability
1
0.75
0.5
0.25
0
0
250
500
750
1,000
1,250
1,500
1,750
Overall survival
Custom survival time cutoff
1780

Table S12. HOX genes previously marked as potential cancer biomarkers[1].

Cancer type	HOX genes	References
**BRCA**	*HOXB13*	[13]
**COAD**	*HOXC11*	[14]
**ESCA**	*HOXB5*, *HOXB6*, *HOXB7*	[15]
**LGG + GBM**	*HOXA1*, *HOXA2*, *HOXA4*, *HOXA6*, *HOXA7*, *HOXA10*, *HOXA11*, *HOXA13*<br>*HOXB7*, *HOXB8*, *HOXB9*, *HOXB13*<br>*HOXC4*, *HOXC5*, *HOXC8*, *HOXC9*, *HOXC11*, *HOXC13*<br>*HOXD4*, *HOXD8*, *HOXD9*, *HOXD10*, *HOXD11*, *HOXD12*, *HOXD13*	[16–22]
**LAML**	*HOXA6*, *HOXA7*, *HOXA9*<br>*HOXB5*, *HOXB6*	[23–28]
**LIHC**	*HOXA13*, *HOXD9*, *HOXD10*	[29–32]
**LUAD**	*HOXA13*,<br>*HOXB9*, *HOXB13*	[33–36]
**LUSC**	*HOXC10*	[37]
**PAAD**	*HOXA11*, *HOXA13*<br>*HOXC11*	[38,39]
**STAD**	*HOXB13*, *HOXC9*	[40,41]

[1]The *HOX* genes that were known as potential cancer biomarker. The 'References' column lists for each cancer type selected references in which the listed *HOX* genes were suggested as biomarker.

References

1. Huang, F.L.; Yu, S.J. Esophageal cancer: Risk factors, genetic association, and treatment. *Asian J. Surg.* **2018**, *41*, 210–215, doi:10.1016/j.asjsur.2016.10.005.
2. Battaglin, F.; Naseem, M.; Puccini, A.; Lenz, H.J. Molecular biomarkers in gastro-esophageal cancer: recent developments, current trends and future directions. *Cancer Cell Int.* **2018**, *18*, 99, doi:10.1186/s12935-018-0594-z.
3. Wang, F.; Zheng, Z.; Guan, J.; Qi, D.; Zhou, S.; Shen, X.; Wang, F.; Wenkert, D.; Kirmani, B.; Solouki, T., et al. Identification of a panel of genes as a prognostic biomarker for glioblastoma. *EBioMedicine* **2018**, *37*, 68–77, doi:10.1016/j.ebiom.2018.10.024.
4. Sasmita, A.O.; Wong, Y.P.; Ling, A.P.K. Biomarkers and therapeutic advances in glioblastoma multiforme. *Asia Pac. J. Clin. Oncol.* **2018**, *14*, 40-51, doi:10.1111/ajco.12756.
5. Handschuh, L. Not Only Mutations Matter: Molecular Picture of Acute Myeloid Leukemia Emerging from Transcriptome Studies. *J. Oncol.* **2019**, *2019*, 7239206, doi:10.1155/2019/7239206.
6. Dong, Q.; Li, Q.; Wang, M.; Hu, J.; Dai, J.; Niu, L.; Yuan, G.; Pan, Y. Elevated CD44 expression predicts poor prognosis in patients with low-grade glioma. *Oncol Lett* **2019**, *18*, 3698–3704, doi:10.3892/ol.2019.10728.
7. Menyhart, O.; Nagy, A.; Gyorffy, B. Determining consistent prognostic biomarkers of overall survival and vascular invasion in hepatocellular carcinoma. *Royal Society open science* **2018**, *5*, 181006, doi:10.1098/rsos.181006.
8. Nwosu, Z.C.; Megger, D.A.; Hammad, S.; Sitek, B.; Roessler, S.; Ebert, M.P.; Meyer, C.; Dooley, S. Identification of the Consistently Altered Metabolic Targets in Human Hepatocellular Carcinoma. *Cellular and molecular gastroenterology and hepatology* **2017**, *4*, 303–323, doi:10.1016/j.jcmgh.2017.05.004.
9. Assmar, M.; Yeganeh, S.; Mansourghanaei, F.; Amirmozafari, N. Combined Evaluation of AFP, CA15-3, CA125, CA19-9, and CEA Tumor Markers in Patients with Hepatitis B and C. *Iranian journal of public health* **2016**, *45*, 1645–1651.
10. Man, J.; Zhang, X.; Dong, H.; Li, S.; Yu, X.; Meng, L.; Gu, X.; Yan, H.; Cui, J.; Lai, Y. Screening and identification of key biomarkers in lung squamous cell carcinoma by bioinformatics analysis. *Oncol Lett* **2019**, *18*, 5185–5196, doi:10.3892/ol.2019.10873.
11. Loosen, S.H.; Neumann, U.P.; Trautwein, C.; Roderburg, C.; Luedde, T. Current and future biomarkers for pancreatic adenocarcinoma. *Tumour biology : the journal of the International Society for Oncodevelopmental Biology and Medicine* **2017**, *39*, 1010428317692231, doi:10.1177/1010428317692231.
12. Virgilio, E.; Proietti, A.; D'Urso, R.; Cardelli, P.; Giarnieri, E.; Montagnini, M.; Giovagnoli, M.R.; Mercantini, P.; Balducci, G.; Cavallini, M. Measuring Intragastric Tumor Markers in Gastric Cancer Patients: a

Systematic Literature Review on Significance and Reliability. *Anticancer research* **2017**, *37*, 2817–2821, doi:10.21873/anticanres.11632.
13. Shah, N.; Kideok, J.; Cruz, L.-A.; Park, S.; Sadik, H.; Cho, S.; Goswami, C.P.; Nakshatri, H.; Gupta, R.; Chang, H.Y.; et al. Molecular and Cellular Pathobiology HOXB13 Mediates Tamoxifen Resistance and Invasiveness in Human Breast Cancer by Suppressing ERa and Inducing IL-6 Expression. *Cancer Res.* **2013**, doi:10.1158/0008-5472.CAN-13-1178.
14. Cui, Y.; Zhang, C.; Wang, Y.; Ma, S.; Cao, W.; Guan, F. HOXC11 functions as a novel oncogene in human colon adenocarcinoma and kidney renal clear cell carcinoma. *Life Sci.* **2020**, *243*, doi:10.1016/j.lfs.2019.117230.
15. Di Pietro, M.; Alzoubaidi, D.; Fitzgerald, R.C. Barrett's esophagus and cancer risk: How research advances can impact clinical practice. *Gut Liver* **2014**, *8*, 356–370.
16. Eoh, K.J.; Kim, H.J.; Lee, J.-Y.; Nam, E.J.; Kim, S.; Kim, S.W.; Kim, Y.T. Upregulation of homeobox gene is correlated with poor survival outcomes in cervical cancer. *Oncotarget* **2017**, *8*, 84396–84402.
17. Li, B.; McCrudden, C.M.; Yuen, H.F.; Xi, X.; Lyu, P.; Chan, K.W.; Zhang, S.D.; Kwok, H.F. CD133 in brain tumor: the prognostic factor. *Oncotarget* **2017**, *8*, 11144–11159.
18. Dong, C.-Y.; Cui, J.; Li, D.-H.; Li, Q.; Hong, X.-Y. HOXA10-AS: A novel oncogenic long non-coding RNA in glioma. *Oncol. Rep.* **2018**, *40*, 2573.
19. Cai, Y.-D.; Zhang, S.; Zhang, Y.-H.; Pan, X.; Feng, K.; Chen, L.; Huang, T.; Kong, X. Identification of the Gene Expression Rules That Define the Subtypes in Glioma. *J. Clin. Med.* **2018**, *7*.
20. Duan, R.; Han, L.; Wang, Q.; Wei, J.; Chen, L.; Zhang, J.; Kang, C.; Wang, L. HOXA13 is a potential GBM diagnostic marker and promotes glioma invasion by activating the Wnt and TGF-β pathways. *Oncotarget* **2015**, *6*, 27778.
21. Liang, T.; Wang, X.; Li, P.; Cao, Y.; Feng, E.; You, G. HOXC8: a predictive glioma biomarker that induces epithelia-mesenchymal transition. *Chinese Neurosurg. J.* **2018**, *4*, 24.
22. Huo, X.-Y.; Zhang, X.-Y.; Yuan, F.; Zhao, X.-Y.; You, B.-A. HOXB7 promotes proliferation and metastasis of glioma by regulating the Wnt/β-catenin pathway. *Eur. Rev. Med. Pharmacol. Sci.* **2019**, *23*, 2476–2485.
23. Starkova, J.; Zamostna, B.; Mejstrikova, E.; Krejci, R.; Drabkin, H.A.; Trka, J. HOX gene expression in phenotypic and genotypic subgroups and low HOXA gene expression as an adverse prognostic factor in pediatric ALL. *Pediatr. Blood Cancer* **2010**, *55*, 1072–1082.
24. Giampaolo, A.; Felli, N.; Diverio, D.; Morsilli, O.; Samoggia, P.; Breccia, M.; Lo Coco, F.; Peschle, C.; Testa, U. Expression pattern of HOXB6 homeobox gene in myelomonocytic differentiation and acute myeloid leukemia. *Leukemia* **2002**, *16*, 1293–1301.
25. Fischbach, N.A.; Rozenfeld, S.; Shen, W.; Fong, S.; Chrobak, D.; Ginzinger, D.; Kogan, S.C.; Radhakrishnan, A.; Le Beau, M.M.; Largman, C.; et al. HOXB6 overexpression in murine bone marrow immortalizes a myelomonocytic precursor in vitro and causes hematopoietic stem cell expansion and acute myeloid leukemia in vivo. *Blood* **2005**, *105*, 1456–1466.
26. Abdel-Fattah, R.; Xiao, A.; Bomgardner, D.; Pease, C.-S.; Lopes, M.-B.; Hussaini, I. Differential expression of HOX genes in neoplastic and non-neoplastic human astrocytes. *J. Pathol.* **2006**, *209*, 15–24.
27. Drabkin, H.; Parsy, C.; Ferguson, K.; Guilhot, F.; Lacotte, L.; Roy, L.; Zeng, C.; Baron, A.; Hunger, S.; Varella-Garcia, M.; et al. Quantitative HOX expression in chromosomally defined subsets of acute myelogenous leukemia. *Leukemia* **2002**, *16*, 186–195.
28. Handschuh, L. Not Only Mutations Matter: Molecular Picture of Acute Myeloid Leukemia Emerging from Transcriptome Studies. *J. Oncol.* **2019**, *2019*.
29. Quagliata, L.; Matter, M.S.; Piscuoglio, S.; Arabi, L.; Ruiz, C.; Procino, A.; Kovac, M.; Moretti, F.; Makowska, Z.; Boldanova, T.; et al. Long noncoding RNA HOTTIP/HOXA13 expression is associated with disease progression and predicts outcome in hepatocellular carcinoma patients. *Hepatology* **2014**, *59*, 911–923.
30. Long, J.; Zhang, L.; Wan, X.; Lin, J.; Bai, Y.; Xu, W.; Xiong, J.; Zhao, H. A four-gene-based prognostic model predicts overall survival in patients with hepatocellular carcinoma. *J. Cell. Mol. Med.* **2018**, *22*, 5928–5938.
31. Guo, Y.; Peng, Y.; Gao, D.; Zhang, M.; Yang, W.; Linghu, E.; Herman, J.G.; Fuks, F.; Dong, G.; Guo, M. Silencing HOXD10 by promoter region hypermethylation activates ERK signaling in hepatocellular carcinoma. *Clin. Epigenetics* **2017**, *9*, 116.
32. Lv, X.; Li, L.; Lv, L.; Qu, X.; Jin, S.; Li, K.; Deng, X.; Cheng, L.; He, H.; Dong, L. HOXD9 promotes epithelial-mesenchymal transition and cancer metastasis by ZEB1 regulation in hepatocellular carcinoma. *J. Exp. Clin. Cancer Res.* **2015**, *34*.

33. Zhan, J.; Wang, P.; Niu, M.; Wang, Y.; Zhu, X.; Guo, Y.; Zhang, H. High expression of transcriptional factor HoxB9 predicts poor prognosis in patients with lung adenocarcinoma. *Histopathology* **2015**, *66*, 955–965.
34. Deng, Y.; He, R.; Zhang, R.; Gan, B.; Zhang, Y.; Chen, G.; Hu, X. The expression of HOXA13 in lung adenocarcinoma and its clinical significance: A study based on The Cancer Genome Atlas, Oncomine and reverse transcription-quantitative polymerase chain reaction. *Oncol. Lett.* **2018**, *15*, 8556–8572.
35. KANG, J.U. Characterization of amplification patterns and target genes on the short arm of chromosome 7 in early-stage lung adenocarcinoma. *Mol. Med. Rep.* **2013**, *8*, 1373–1378.
36. Zhan, J.; Wang, P.; Li, S.; Song, J.; He, H.; Wang, Y.; Liu, Z.; Wang, F.; Bai, H.; Fang, W.; et al. HOXB13 networking with ABCG1/EZH2/Slug mediates metastasis and confers resistance to cisplatin in lung adenocarcinoma patients. *Theranostics* **2019**, *9*, 2084–2099.
37. Tang, X.-L.; Ding, B.-X.; Hua, Y.; Chen, H.; Wu, T.; Chen, Z.-Q.; Yuan, C.-H. HOXC10 Promotes the Metastasis of Human Lung Adenocarcinoma and Indicates Poor Survival Outcome. *Front. Physiol.* **2017**, *8*.
38. Cheng, Y.; Jutooru, I.; Chadalapaka, G.; Corton, C.J.; Safe, S. The long non-coding RNA HOTTIP enhances pancreatic cancer cell proliferation, survival and migration. *Oncotarget* **2015**, *6*, 10840–52.
39. Li, Z.; Zhao, X.; Zhou, Y.; Liu, Y.; Zhou, Q.; Ye, H.; Wang, Y.; Zeng, J.; Song, Y.; Gao, W.; et al. The long non-coding RNA HOTTIP promotes progression and gemcitabine resistance by regulating HOXA13 in pancreatic cancer. *J. Transl. Med.* **2015**, *13*, 84.
40. Sui, B.Q.; Zhang, C.D.; Liu, J.C.; Wang, L.; Dai, D.Q. HOXB13 expression and promoter methylation as a candidate biomarker in gastric cancer. *Oncol. Lett.* **2018**, *15*, 8833–8840.
41. Moon, S.; Hong, C.; Lee, S.; Zhao, X.-F.; Lee, H.; Kim, S.; Han, S.-U.; Chung, I.-J.; Park, Y.-K. Abstract 1032: Impact of HOXC9 recapitulation on cancer biology and clinical outcome in gastric cancer.; American Association for Cancer Research (AACR), 2010; pp. 1032–1032.

© 2020 by the authors. Submitted for possible open access publication under the terms and conditions of the Creative Commons Attribution (CC BY) license (http://creativecommons.org/licenses/by/4.0/).
