# Supplementary material for: Quantitative Analysis of Differential Expression of HOX Genes in Multiple Cancers
Source: Cancers (Basel). 2020 Jun 14;12(6):1572. doi: 10.3390/cancers12061572 (PMC7352544; doi:10.3390/cancers12061572)
Supplement: Supplementary file 1 [file cancers-12-01572-s001.zip › Supplementary File S1.pdf]

## Supplementary File S1

KM plots of *HOX* gene pairs (a) versus separate KM plots for each *HOX* gene (b and c) in GBM and LGG.

For every gene in a pair, the samples were divided to two groups: the group of samples in which the gene expression is higher than the median, and a group in which the gene expression is lower than the median. The KM analysis of each gene pair compared 2 groups of samples:

- The group created by intersection of the 2 groups of samples with expression higher than the median expression of every gene (marked in red in the plots and the risk tables below), N1 denotes the size of the group
- The group created by intersection between the samples with expression lower than the median expression of every gene (marked in blue in the plots and the risk tables below), N2 denotes the size of this group

In addition, a KM survival analysis was performed for each gene separately. For each single gene KM analysis, we used the N1 samples with the highest expression and the N2 samples with the lowest expression. The survival analysis of the pair of genes (a) was then compared with the survival analysis of every one of the genes separately (b and c). Depicted are gene pairs with KM-based p-value < 0.05 (Bonferroni-corrected for multiple gene pairs) and a p-value smaller than the p-value of the survival analysis of each of the two genes separately. The pink and the light blue background of the lines mark the confidence interval.

| HOX pair | N | N1 | N2 |
|---|---|---|---|
| GBM - *HOXB2*:*HOXB9* | 173 | 49 | 50 |
| GBM - *HOXB2*:*HOXC13* | 173 | 51 | 52 |
| GBM - *HOXB9*:*HOXC8* | 173 | 48 | 49 |
| GBM - *HOXB9*:*HOXC10* | 173 | 50 | 51 |
| LGG - *HOXA1*:*HOXA4* | 527 | 183 | 182 |
| LGG - *HOXA1*:*HOXA7* | 527 | 173 | 172 |
| LGG - *HOXA1*:*HOXD4* | 527 | 153 | 152 |
| LGG - *HOXA4*:*HOXA7* | 527 | 173 | 172 |
| LGG - *HOXA4*:*HOXD3* | 527 | 164 | 163 |
| LGG - *HOXA4*:*HOXD4* | 527 | 159 | 158 |
| LGG - *HOXA4*:*HOXD10* | 527 | 160 | 159 |
| LGG - *HOXA7*:*HOXB13* | 527 | 157 | 157 |
| LGG - *HOXA7*: *HOXC4* | 527 | 180 | 180 |
| LGG - *HOXA7*:*HOXD4* | 527 | 168 | 167 |
| LGG - *HOXA7*:*HOXD10* | 527 | 168 | 167 |
| LGG - *HOXA11*:*HOXD4* | 527 | 164 | 163 |
| LGG - *HOXA13*:*HOXB7* | 527 | 177 | 176 |
| LGG - *HOXA13*:*HOXD3* | 527 | 167 | 166 |
| LGG - *HOXA13*:*HOXD4* | 527 | 161 | 160 |
| LGG - *HOXA13*:*HOXD8* | 527 | 176 | 175 |
| LGG - *HOXA13*:*HOXD10* | 527 | 160 | 159 |
| LGG - *HOXC4*:*HOXD10* | 527 | 171 | 171 |
| LGG - *HOXD10*:*HOXD4* | 527 | 219 | 218 |

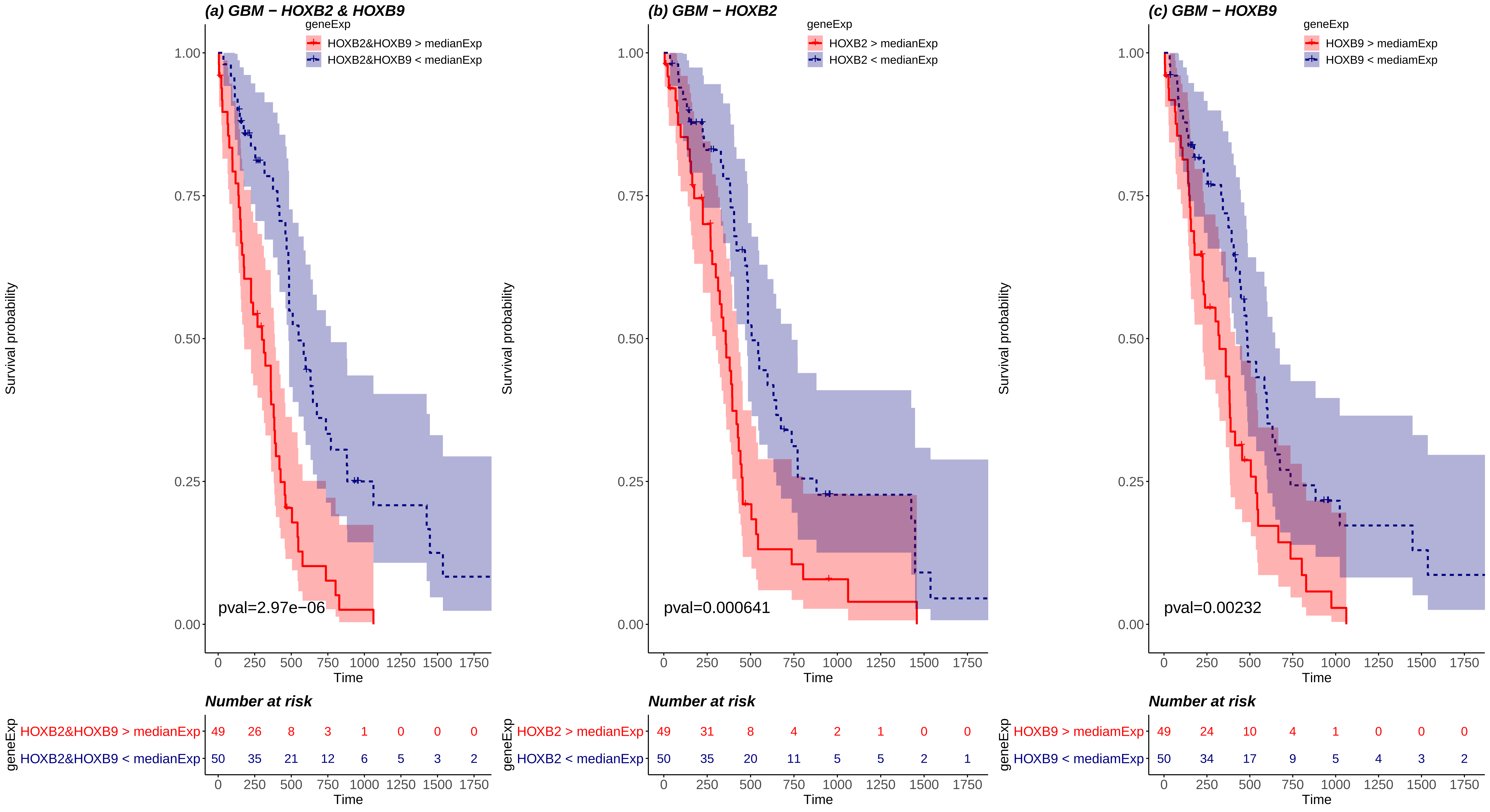
(a) GBM − HOXB2 & HOXB9
geneExp
HOXB2&HOXB9 > medianExp
HOXB2&HOXB9 < medianExp
Survival probability
1.00
0.75
0.50
0.25
0.00
pval=2.97e−06
0 250 500 750 1000 1250 1500 1750
Time
Number at risk
geneExp
HOXB2&HOXB9 > medianExp 49 26 8 3 1 0 0 0
HOXB2&HOXB9 < medianExp 50 35 21 12 6 5 3 2
(b) GBM − HOXB2
HOXB2 > medianExp
HOXB2 < medianExp
pval=0.000641
HOXB2 > medianExp 49 31 8 4 2 1 0 0
HOXB2 < medianExp 50 35 20 11 5 5 2 1
(c) GBM − HOXB9
HOXB9 > mediamExp
HOXB9 < mediamExp
pval=0.00232
HOXB9 > mediamExp 49 24 10 4 1 0 0 0
HOXB9 < mediamExp 50 34 17 9 5 4 3 2

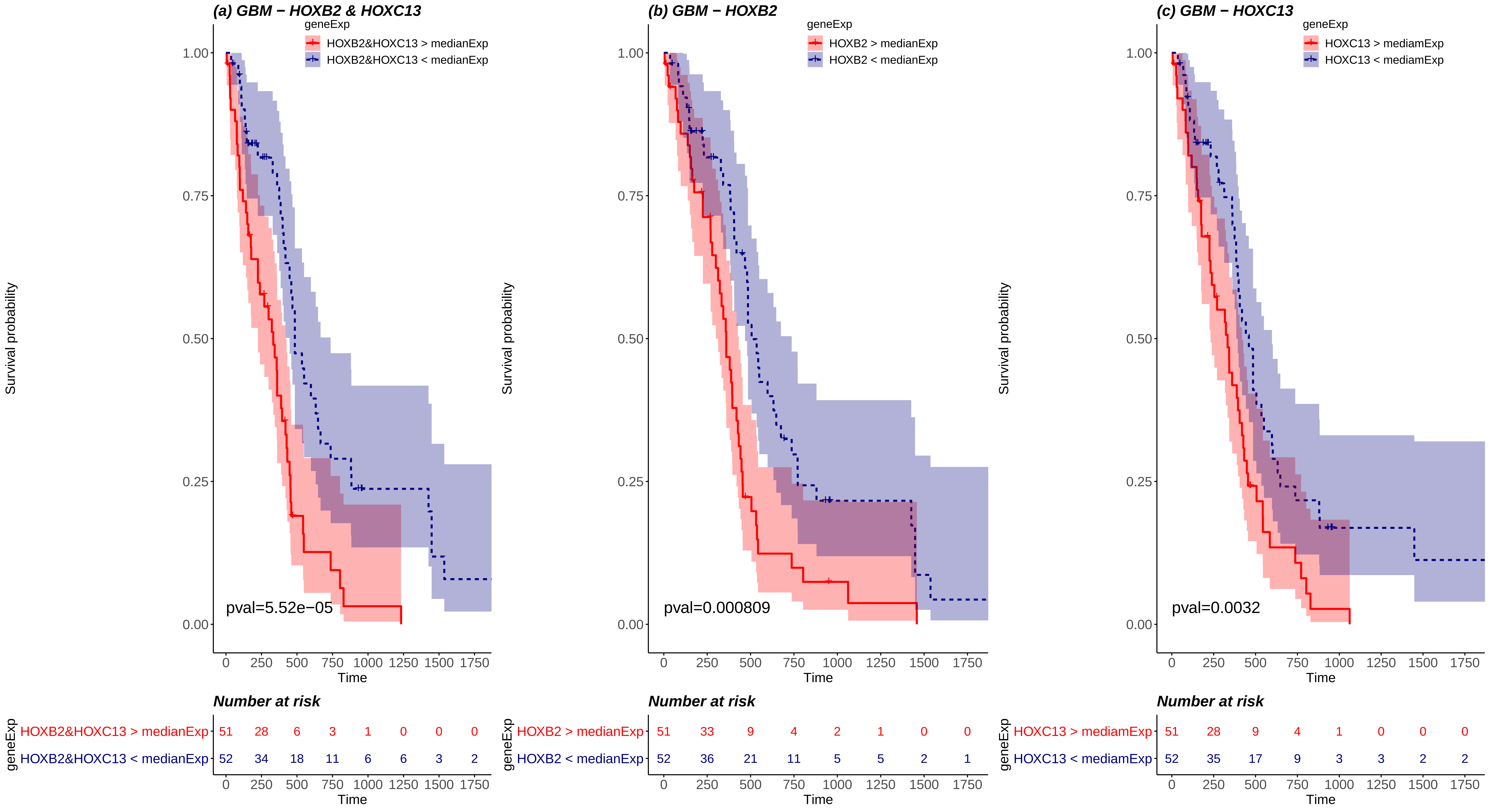

(a) GBM − HOXB2 & HOXC13
geneExp
HOXB2&HOXC13 > medianExp
HOXB2&HOXC13 < medianExp
Survival probability
1.00
0.75
0.50
0.25
0.00
pval=5.52e−05
0 250 500 750 1000 1250 1500 1750
Time
Number at risk
geneExp
HOXB2&HOXC13 > medianExp 51 28 6 3 1 0 0 0
HOXB2&HOXC13 < medianExp 52 34 18 11 6 6 3 2
0 250 500 750 1000 1250 1500 1750
Time
(b) GBM − HOXB2
geneExp
HOXB2 > medianExp
HOXB2 < medianExp
Survival probability
1.00
0.75
0.50
0.25
0.00
pval=0.000809
0 250 500 750 1000 1250 1500 1750
Time
Number at risk
geneExp
HOXB2 > medianExp 51 33 9 4 2 1 0 0
HOXB2 < medianExp 52 36 21 11 5 5 2 1
0 250 500 750 1000 1250 1500 1750
Time
(c) GBM − HOXC13
geneExp
HOXC13 > mediamExp
HOXC13 < mediamExp
Survival probability
1.00
0.75
0.50
0.25
0.00
pval=0.0032
0 250 500 750 1000 1250 1500 1750
Time
Number at risk
geneExp
HOXC13 > mediamExp 51 28 9 4 1 0 0 0
HOXC13 < mediamExp 52 35 17 9 3 3 2 2
0 250 500 750 1000 1250 1500 1750
Time

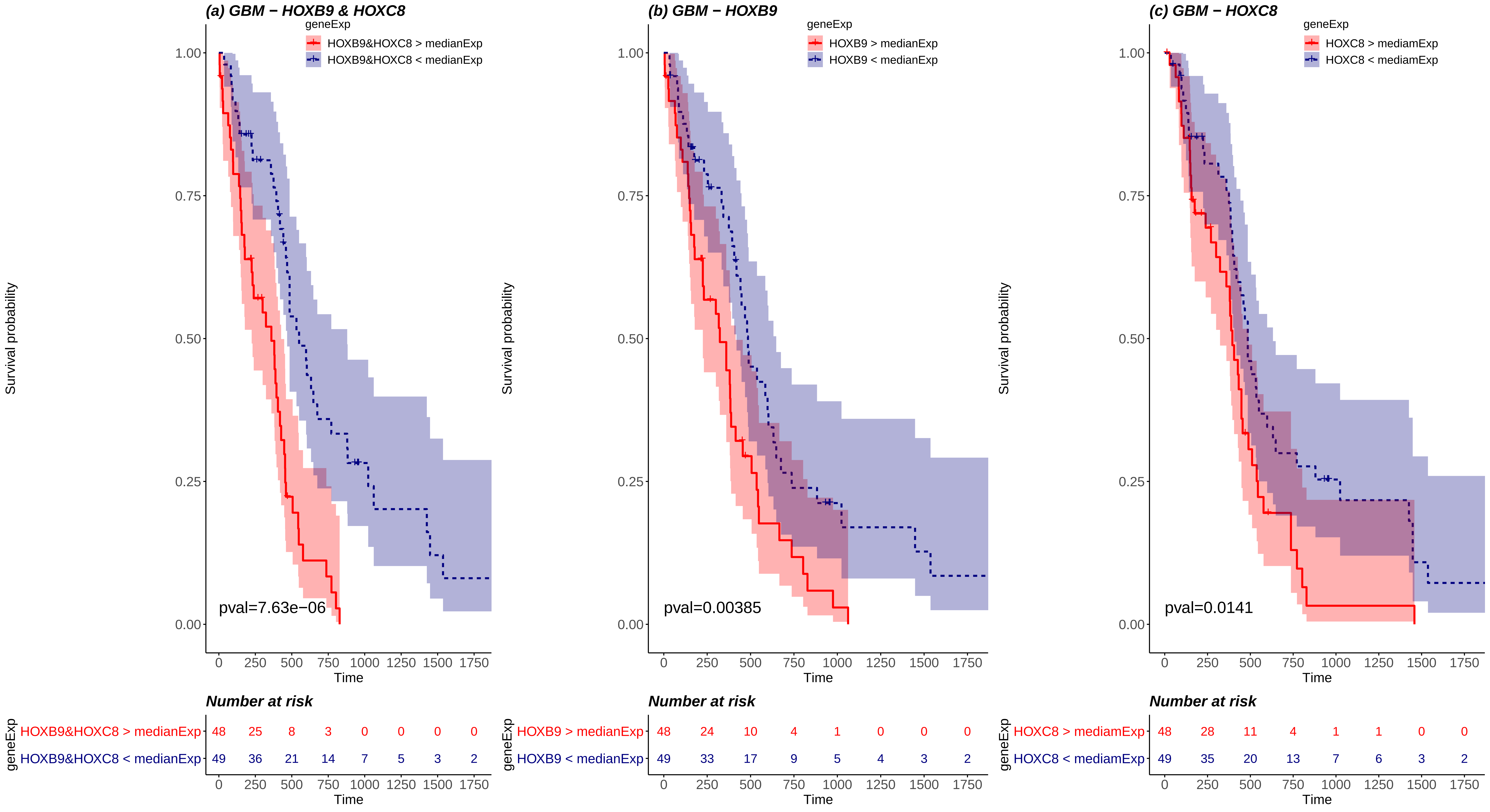
(a) GBM − HOXB9 & HOXC8
geneExp
HOXB9&HOXC8 > medianExp
HOXB9&HOXC8 < medianExp
Survival probability
1.00
0.75
0.50
0.25
0.00
pval=7.63e−06
0 250 500 750 1000 1250 1500 1750
Time
Number at risk
geneExp
HOXB9&HOXC8 > medianExp 48 25 8 3 0 0 0 0
HOXB9&HOXC8 < medianExp 49 36 21 14 7 5 3 2
0 250 500 750 1000 1250 1500 1750
Time
(b) GBM − HOXB9
geneExp
HOXB9 > medianExp
HOXB9 < medianExp
Survival probability
1.00
0.75
0.50
0.25
0.00
pval=0.00385
0 250 500 750 1000 1250 1500 1750
Time
Number at risk
geneExp
HOXB9 > medianExp 48 24 10 4 1 0 0 0
HOXB9 < medianExp 49 33 17 9 5 4 3 2
0 250 500 750 1000 1250 1500 1750
Time
(c) GBM − HOXC8
geneExp
HOXC8 > mediamExp
HOXC8 < mediamExp
Survival probability
1.00
0.75
0.50
0.25
0.00
pval=0.0141
0 250 500 750 1000 1250 1500 1750
Time
Number at risk
geneExp
HOXC8 > mediamExp 48 28 11 4 1 1 0 0
HOXC8 < mediamExp 49 35 20 13 7 6 3 2
0 250 500 750 1000 1250 1500 1750
Time

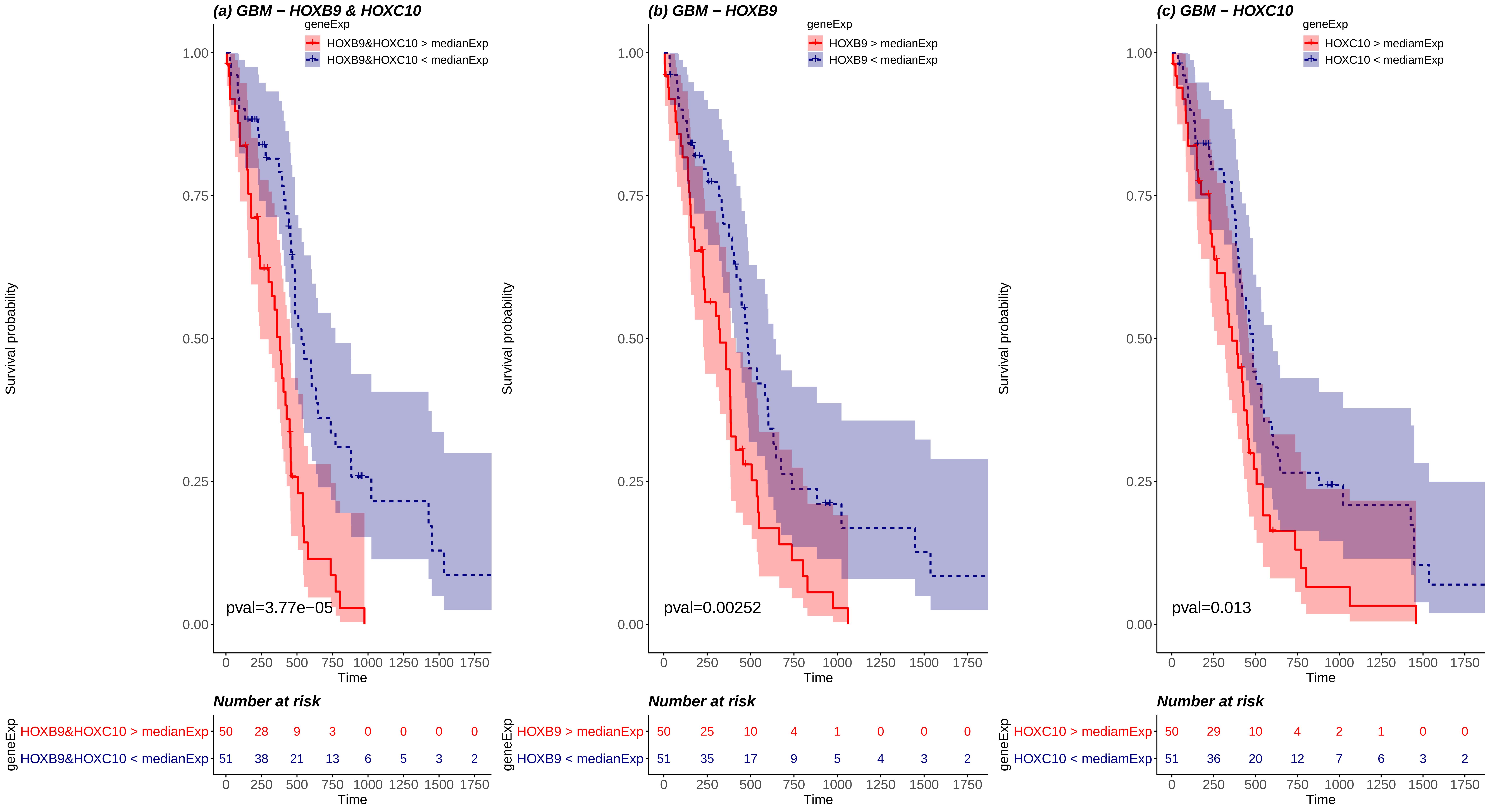

(a) GBM − HOXB9 & HOXC10
geneExp
HOXB9&HOXC10 > medianExp
HOXB9&HOXC10 < medianExp
pval=3.77e−05
Survival probability
Time
Number at risk
HOXB9&HOXC10 > medianExp 50 28 9 3 0 0 0 0
HOXB9&HOXC10 < medianExp 51 38 21 13 6 5 3 2
(b) GBM − HOXB9
HOXB9 > medianExp
HOXB9 < medianExp
pval=0.00252
HOXB9 > medianExp 50 25 10 4 1 0 0 0
HOXB9 < medianExp 51 35 17 9 5 4 3 2
(c) GBM − HOXC10
HOXC10 > mediamExp
HOXC10 < mediamExp
pval=0.013
HOXC10 > mediamExp 50 29 10 4 2 1 0 0
HOXC10 < mediamExp 51 36 20 12 7 6 3 2

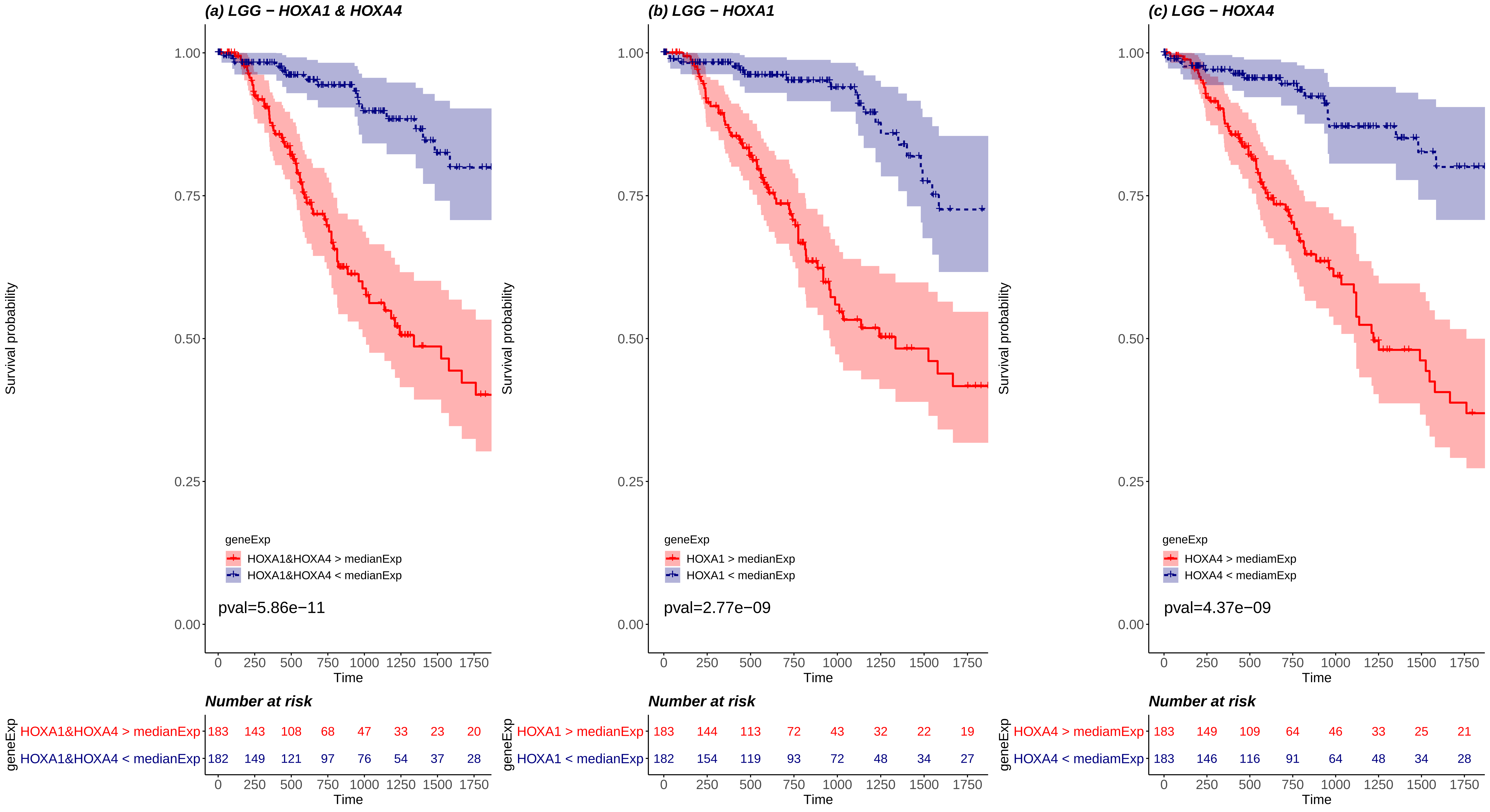

(a) LGG − HOXA1 & HOXA4
(b) LGG − HOXA1
(c) LGG − HOXA4
Survival probability
1.00
0.75
0.50
0.25
0.00
Time
0 250 500 750 1000 1250 1500 1750
geneExp
HOXA1&HOXA4 > medianExp
HOXA1&HOXA4 < medianExp
pval=5.86e−11
HOXA1 > medianExp
HOXA1 < medianExp
pval=2.77e−09
HOXA4 > mediamExp
HOXA4 < mediamExp
pval=4.37e−09
Number at risk
HOXA1&HOXA4 > medianExp 183 143 108 68 47 33 23 20
HOXA1&HOXA4 < medianExp 182 149 121 97 76 54 37 28
HOXA1 > medianExp 183 144 113 72 43 32 22 19
HOXA1 < medianExp 182 154 119 93 72 48 34 27
HOXA4 > mediamExp 183 149 109 64 46 33 25 21
HOXA4 < mediamExp 183 146 116 91 64 48 34 28

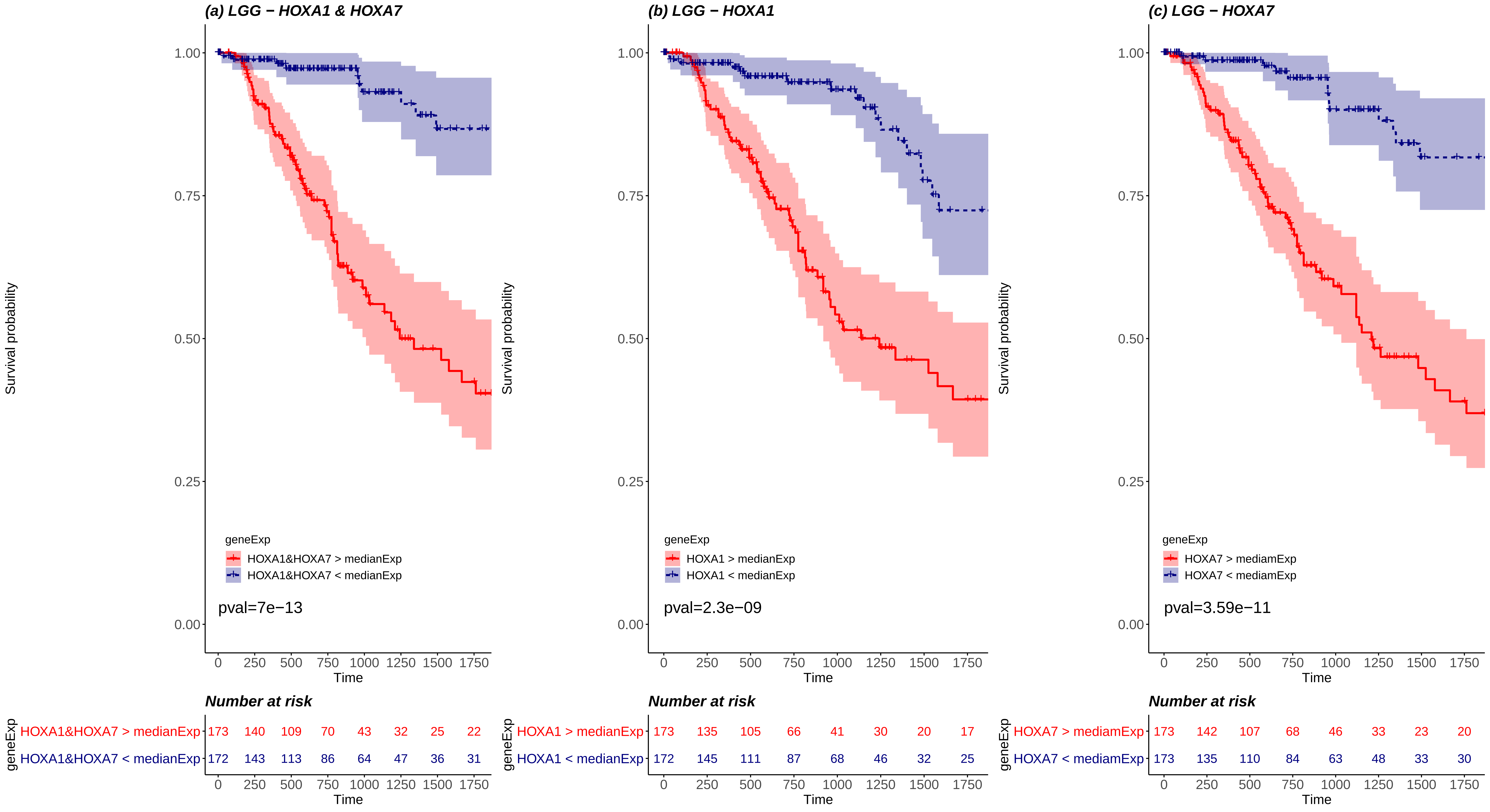
(a) LGG − HOXA1 & HOXA7
(b) LGG − HOXA1
(c) LGG − HOXA7
Survival probability
1.00
0.75
0.50
0.25
0.00
Time
0 250 500 750 1000 1250 1500 1750
geneExp
HOXA1&HOXA7 > medianExp
HOXA1&HOXA7 < medianExp
pval=7e−13
HOXA1 > medianExp
HOXA1 < medianExp
pval=2.3e−09
HOXA7 > mediamExp
HOXA7 < mediamExp
pval=3.59e−11
Number at risk
HOXA1&HOXA7 > medianExp 173 140 109 70 43 32 25 22
HOXA1&HOXA7 < medianExp 172 143 113 86 64 47 36 31
HOXA1 > medianExp 173 135 105 66 41 30 20 17
HOXA1 < medianExp 172 145 111 87 68 46 32 25
HOXA7 > mediamExp 173 142 107 68 46 33 23 20
HOXA7 < mediamExp 173 135 110 84 63 48 33 30

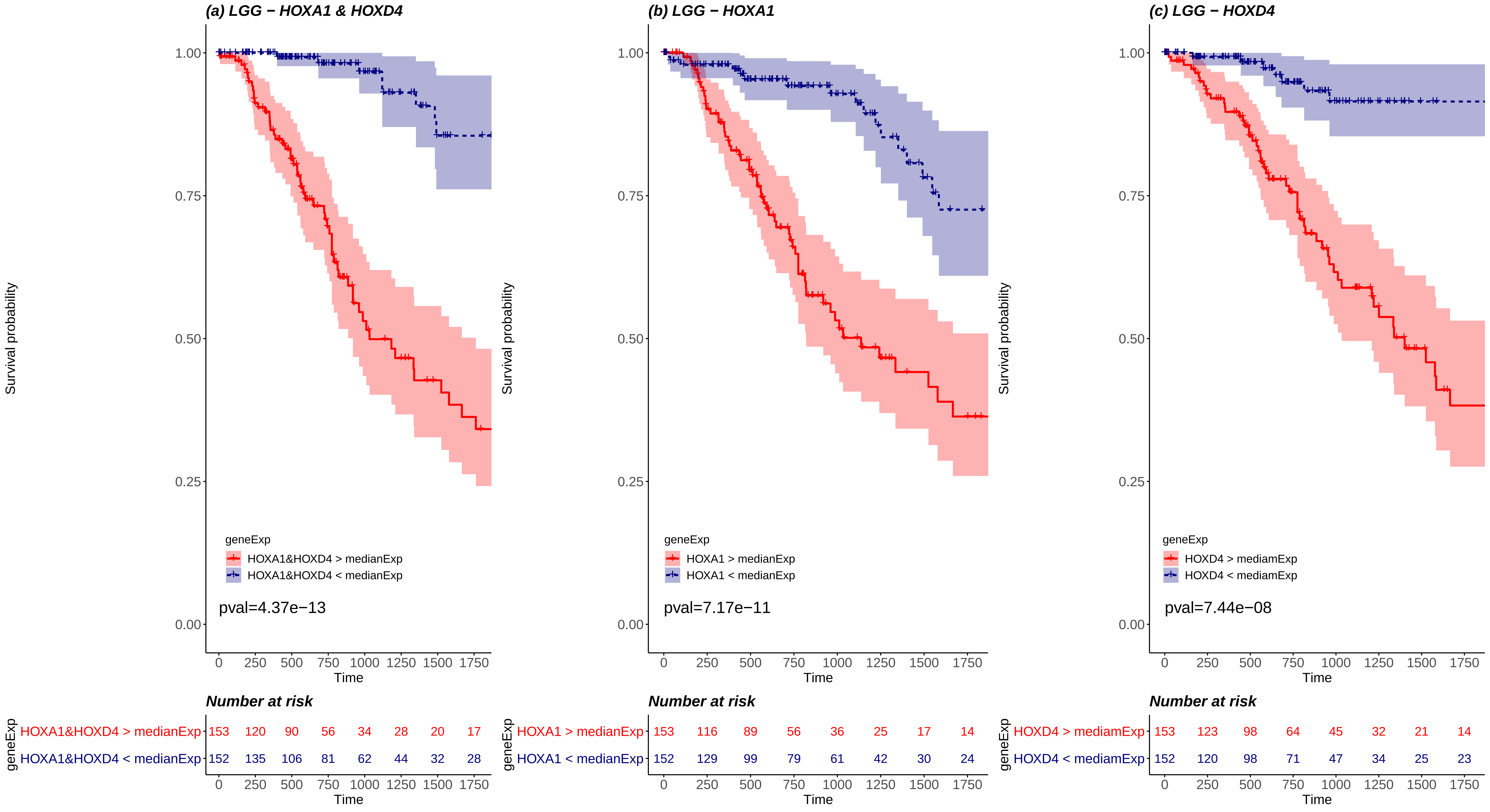

(a) LGG − HOXA1 & HOXD4
(b) LGG − HOXA1
(c) LGG − HOXD4
Survival probability
1.00
0.75
0.50
0.25
0.00
geneExp
HOXA1&HOXD4 > medianExp
HOXA1&HOXD4 < medianExp
pval=4.37e−13
HOXA1 > medianExp
HOXA1 < medianExp
pval=7.17e−11
HOXD4 > mediamExp
HOXD4 < mediamExp
pval=7.44e−08
0 250 500 750 1000 1250 1500 1750
Time
Number at risk
HOXA1&HOXD4 > medianExp 153 120 90 56 34 28 20 17
HOXA1&HOXD4 < medianExp 152 135 106 81 62 44 32 28
HOXA1 > medianExp 153 116 89 56 36 25 17 14
HOXA1 < medianExp 152 129 99 79 61 42 30 24
HOXD4 > mediamExp 153 123 98 64 45 32 21 14
HOXD4 < mediamExp 152 120 98 71 47 34 25 23

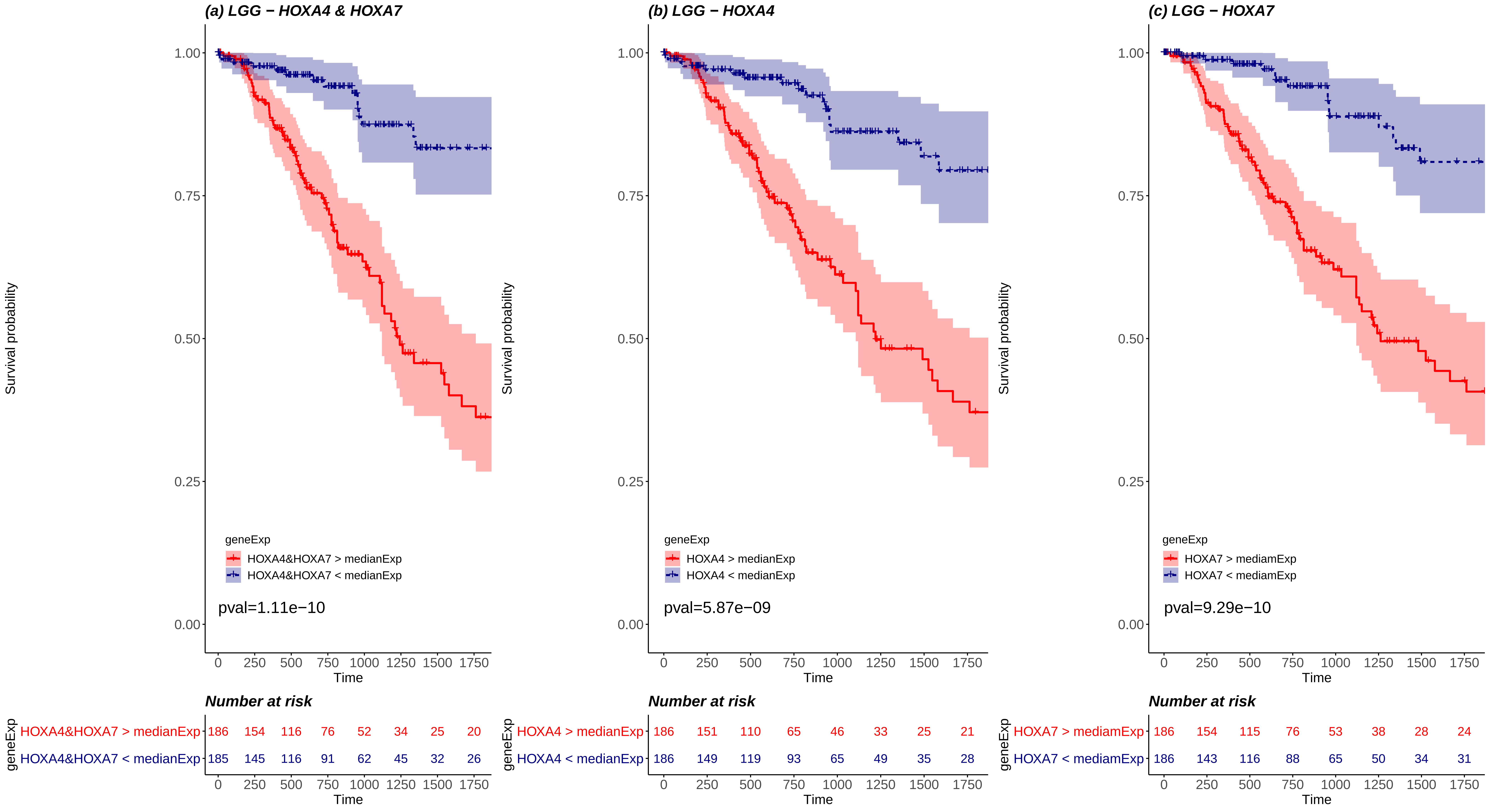

(a) LGG − HOXA4 & HOXA7
(b) LGG − HOXA4
(c) LGG − HOXA7
Survival probability
1.00
0.75
0.50
0.25
0.00
Time
0
250
500
750
1000
1250
1500
1750
geneExp
HOXA4&HOXA7 > medianExp
HOXA4&HOXA7 < medianExp
pval=1.11e−10
HOXA4 > medianExp
HOXA4 < medianExp
pval=5.87e−09
HOXA7 > mediamExp
HOXA7 < mediamExp
pval=9.29e−10
Number at risk
HOXA4&HOXA7 > medianExp 186 154 116 76 52 34 25 20
HOXA4&HOXA7 < medianExp 185 145 116 91 62 45 32 26
HOXA4 > medianExp 186 151 110 65 46 33 25 21
HOXA4 < medianExp 186 149 119 93 65 49 35 28
HOXA7 > mediamExp 186 154 115 76 53 38 28 24
HOXA7 < mediamExp 186 143 116 88 65 50 34 31

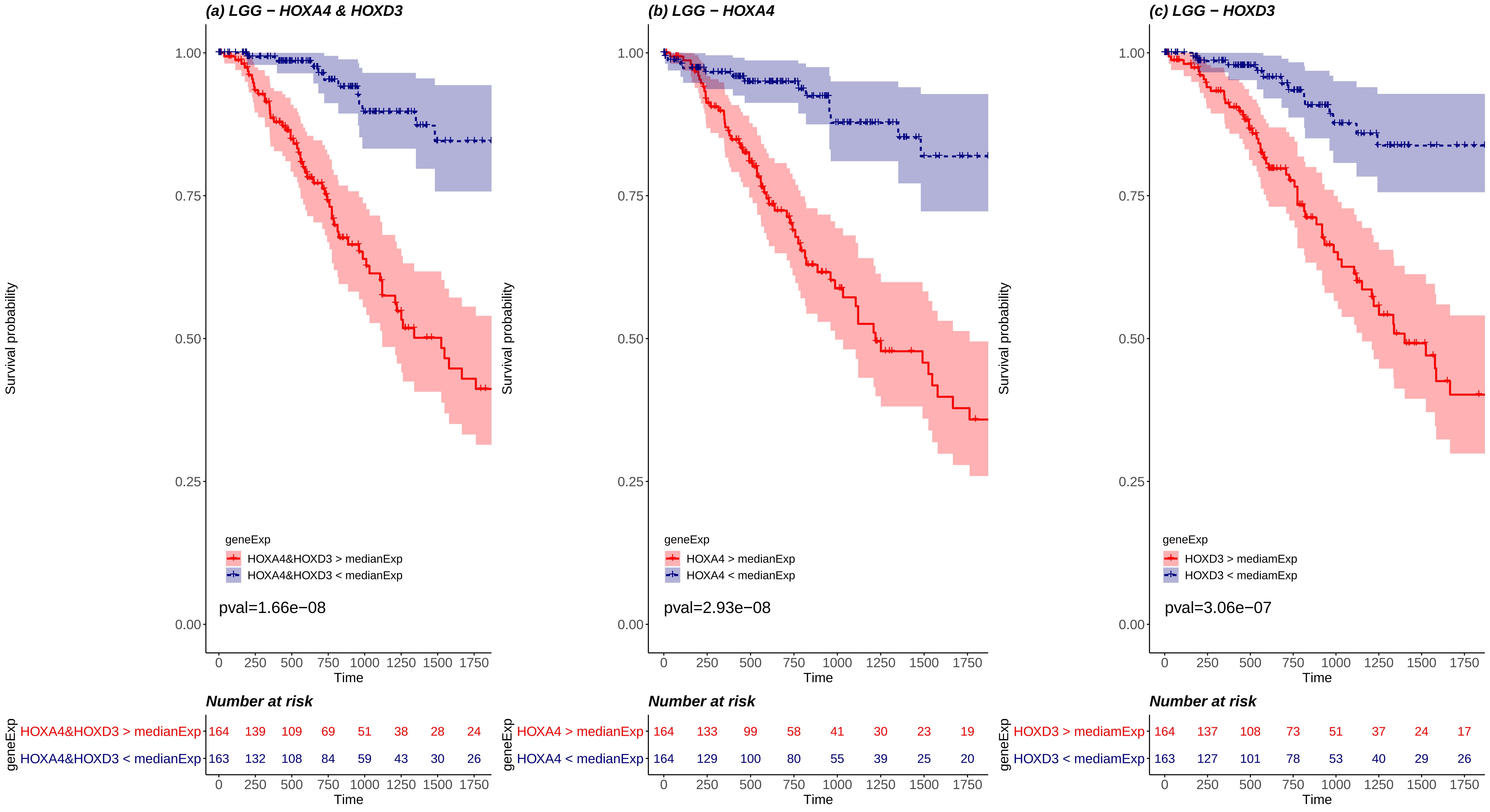
(a) LGG − HOXA4 & HOXD3
(b) LGG − HOXA4
(c) LGG − HOXD3
Survival probability
1.00
0.75
0.50
0.25
0.00
Time
0 250 500 750 1000 1250 1500 1750
geneExp
HOXA4&HOXD3 > medianExp
HOXA4&HOXD3 < medianExp
pval=1.66e−08
HOXA4 > medianExp
HOXA4 < medianExp
pval=2.93e−08
HOXD3 > mediamExp
HOXD3 < mediamExp
pval=3.06e−07
Number at risk
HOXA4&HOXD3 > medianExp 164 139 109 69 51 38 28 24
HOXA4&HOXD3 < medianExp 163 132 108 84 59 43 30 26
HOXA4 > medianExp 164 133 99 58 41 30 23 19
HOXA4 < medianExp 164 129 100 80 55 39 25 20
HOXD3 > mediamExp 164 137 108 73 51 37 24 17
HOXD3 < mediamExp 163 127 101 78 53 40 29 26

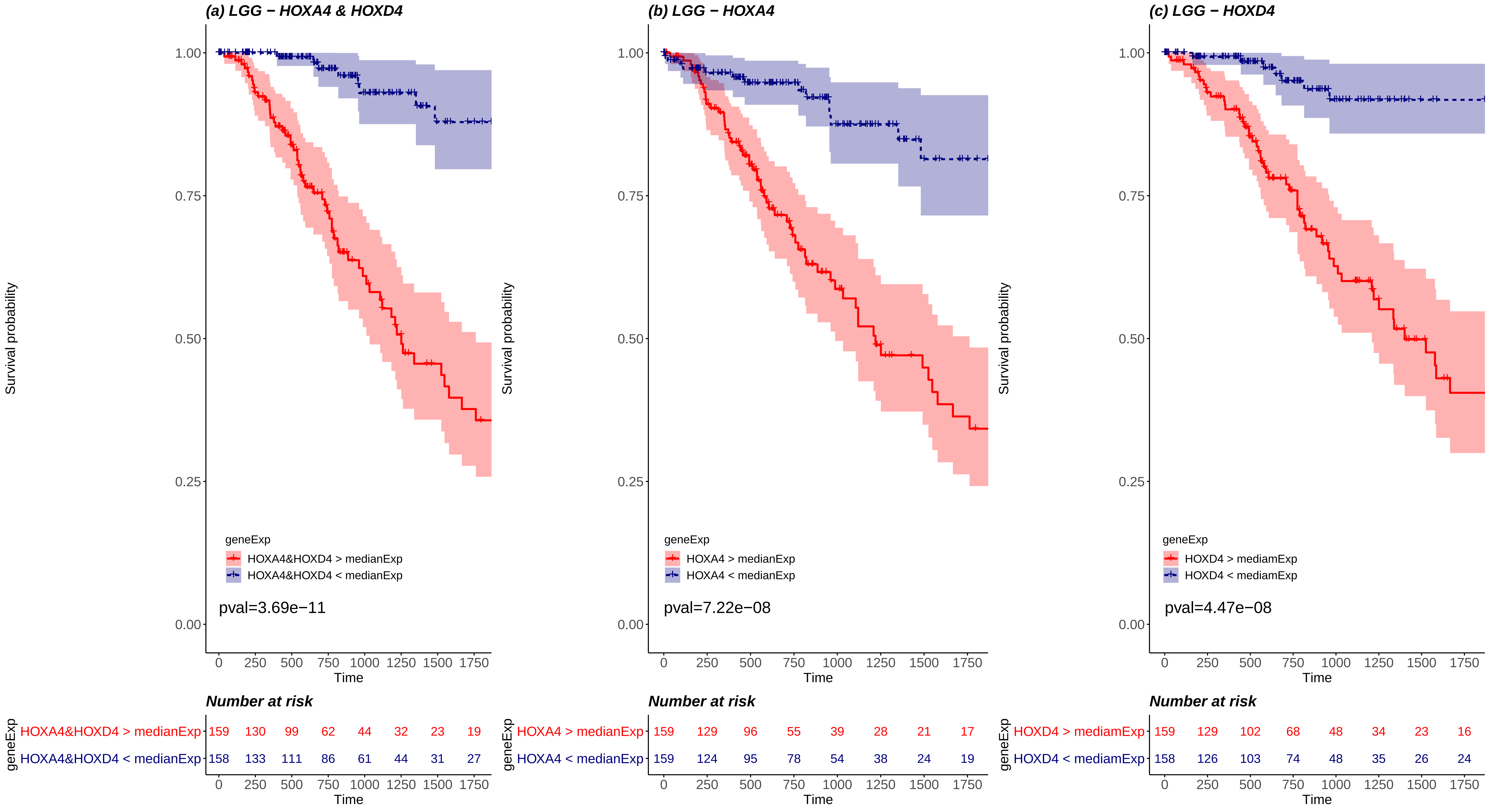

(a) LGG − HOXA4 & HOXD4
(b) LGG − HOXA4
(c) LGG − HOXD4
Survival probability
1.00
0.75
0.50
0.25
0.00
Time
0 250 500 750 1000 1250 1500 1750
geneExp
HOXA4&HOXD4 > medianExp
HOXA4&HOXD4 < medianExp
pval=3.69e−11
HOXA4 > medianExp
HOXA4 < medianExp
pval=7.22e−08
HOXD4 > mediamExp
HOXD4 < mediamExp
pval=4.47e−08
Number at risk
HOXA4&HOXD4 > medianExp 159 130 99 62 44 32 23 19
HOXA4&HOXD4 < medianExp 158 133 111 86 61 44 31 27
HOXA4 > medianExp 159 129 96 55 39 28 21 17
HOXA4 < medianExp 159 124 95 78 54 38 24 19
HOXD4 > mediamExp 159 129 102 68 48 34 23 16
HOXD4 < mediamExp 158 126 103 74 48 35 26 24

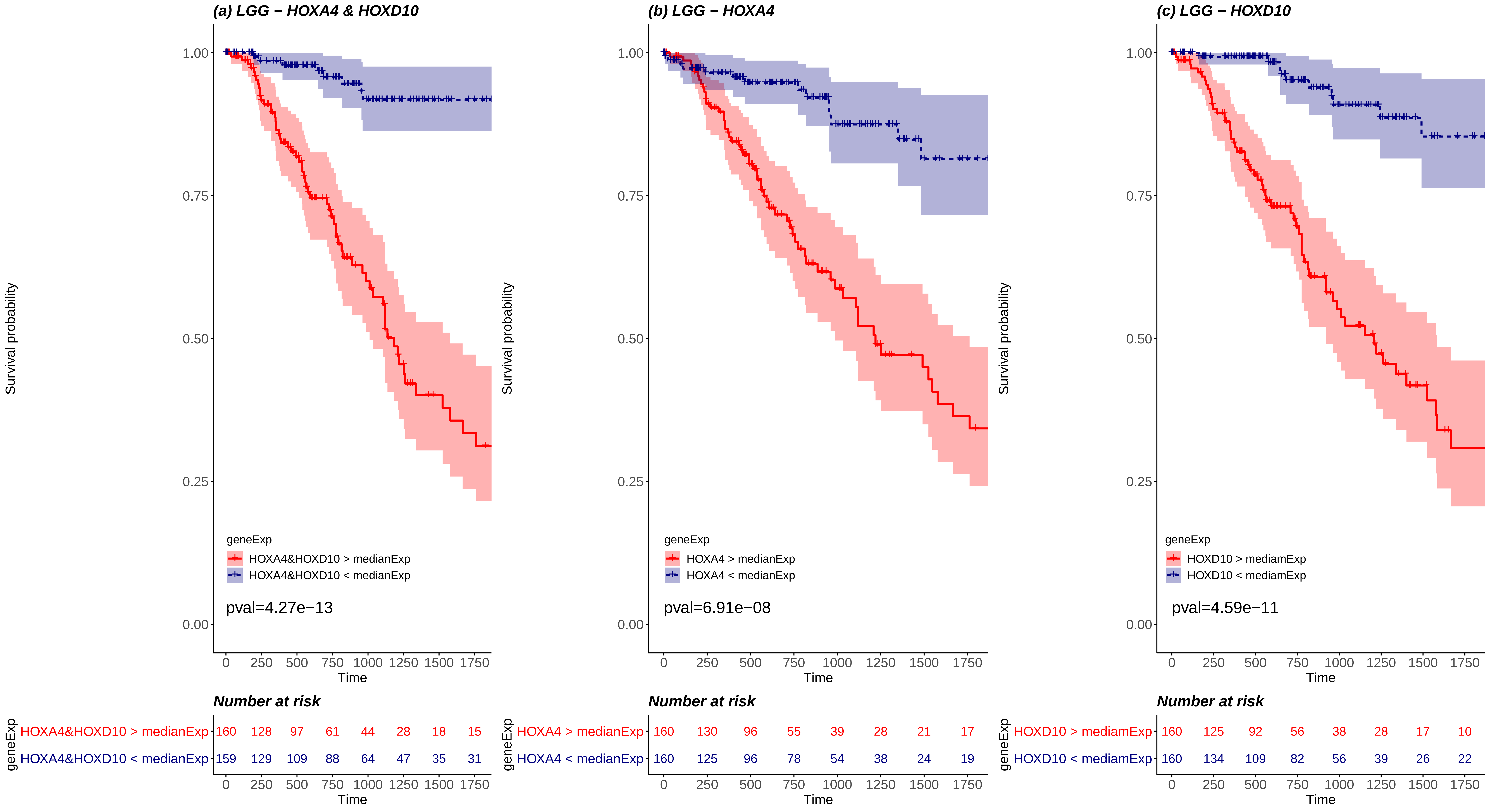
(a) LGG − HOXA4 & HOXD10
(b) LGG − HOXA4
(c) LGG − HOXD10
Survival probability
Time
geneExp
HOXA4&HOXD10 > medianExp
HOXA4&HOXD10 < medianExp
pval=4.27e−13
HOXA4 > medianExp
HOXA4 < medianExp
pval=6.91e−08
HOXD10 > mediamExp
HOXD10 < mediamExp
pval=4.59e−11
Number at risk
HOXA4&HOXD10 > medianExp 160 128 97 61 44 28 18 15
HOXA4&HOXD10 < medianExp 159 129 109 88 64 47 35 31
HOXA4 > medianExp 160 130 96 55 39 28 21 17
HOXA4 < medianExp 160 125 96 78 54 38 24 19
HOXD10 > mediamExp 160 125 92 56 38 28 17 10
HOXD10 < mediamExp 160 134 109 82 56 39 26 22

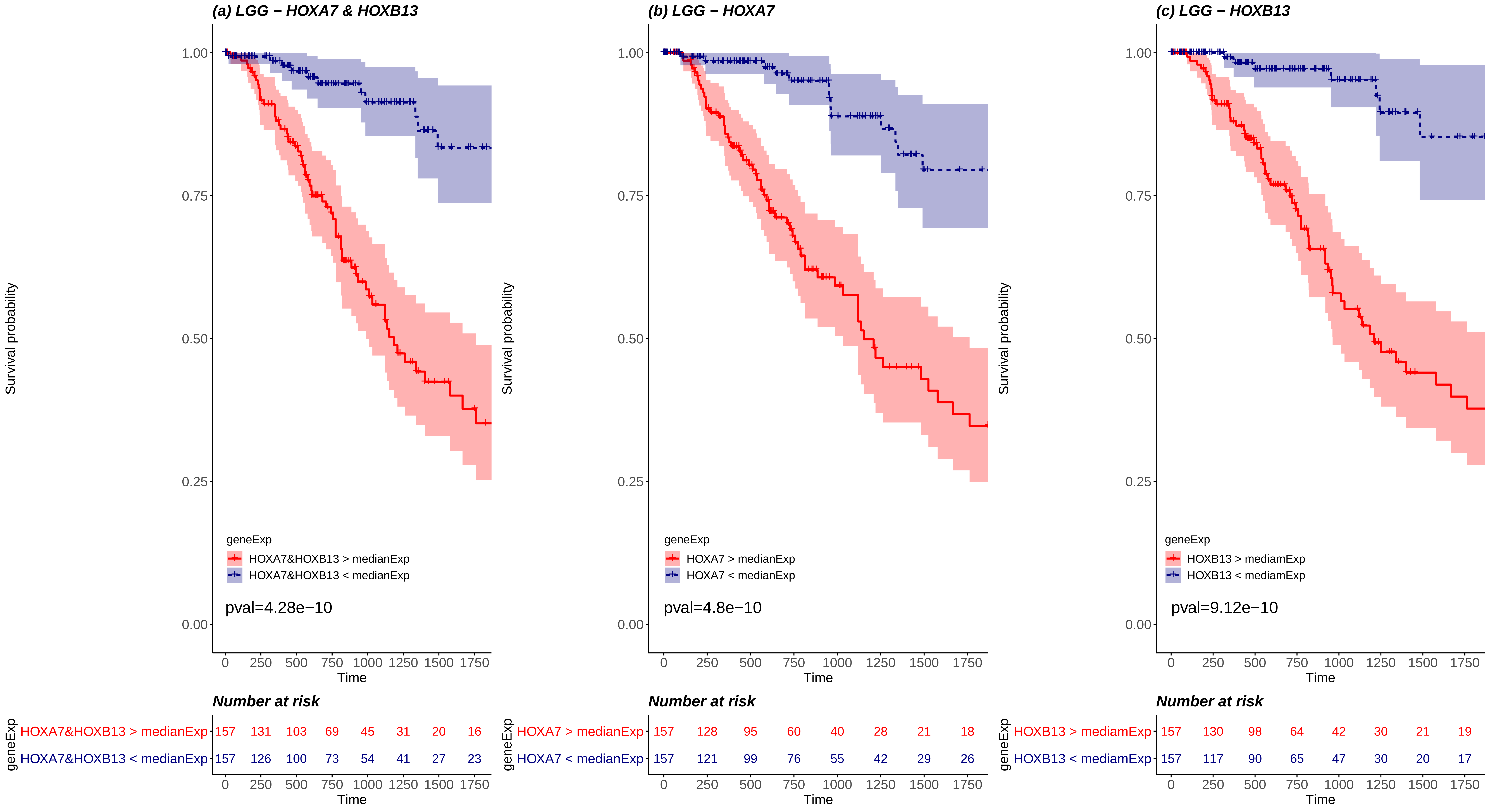

(a) LGG − HOXA7 & HOXB13
(b) LGG − HOXA7
(c) LGG − HOXB13
Survival probability
1.00
0.75
0.50
0.25
0.00
Time
0
250
500
750
1000
1250
1500
1750
geneExp
HOXA7&HOXB13 > medianExp
HOXA7&HOXB13 < medianExp
pval=4.28e−10
HOXA7 > medianExp
HOXA7 < medianExp
pval=4.8e−10
HOXB13 > mediamExp
HOXB13 < mediamExp
pval=9.12e−10
Number at risk
HOXA7&HOXB13 > medianExp 157 131 103 69 45 31 20 16
HOXA7&HOXB13 < medianExp 157 126 100 73 54 41 27 23
HOXA7 > medianExp 157 128 95 60 40 28 21 18
HOXA7 < medianExp 157 121 99 76 55 42 29 26
HOXB13 > mediamExp 157 130 98 64 42 30 21 19
HOXB13 < mediamExp 157 117 90 65 47 30 20 17

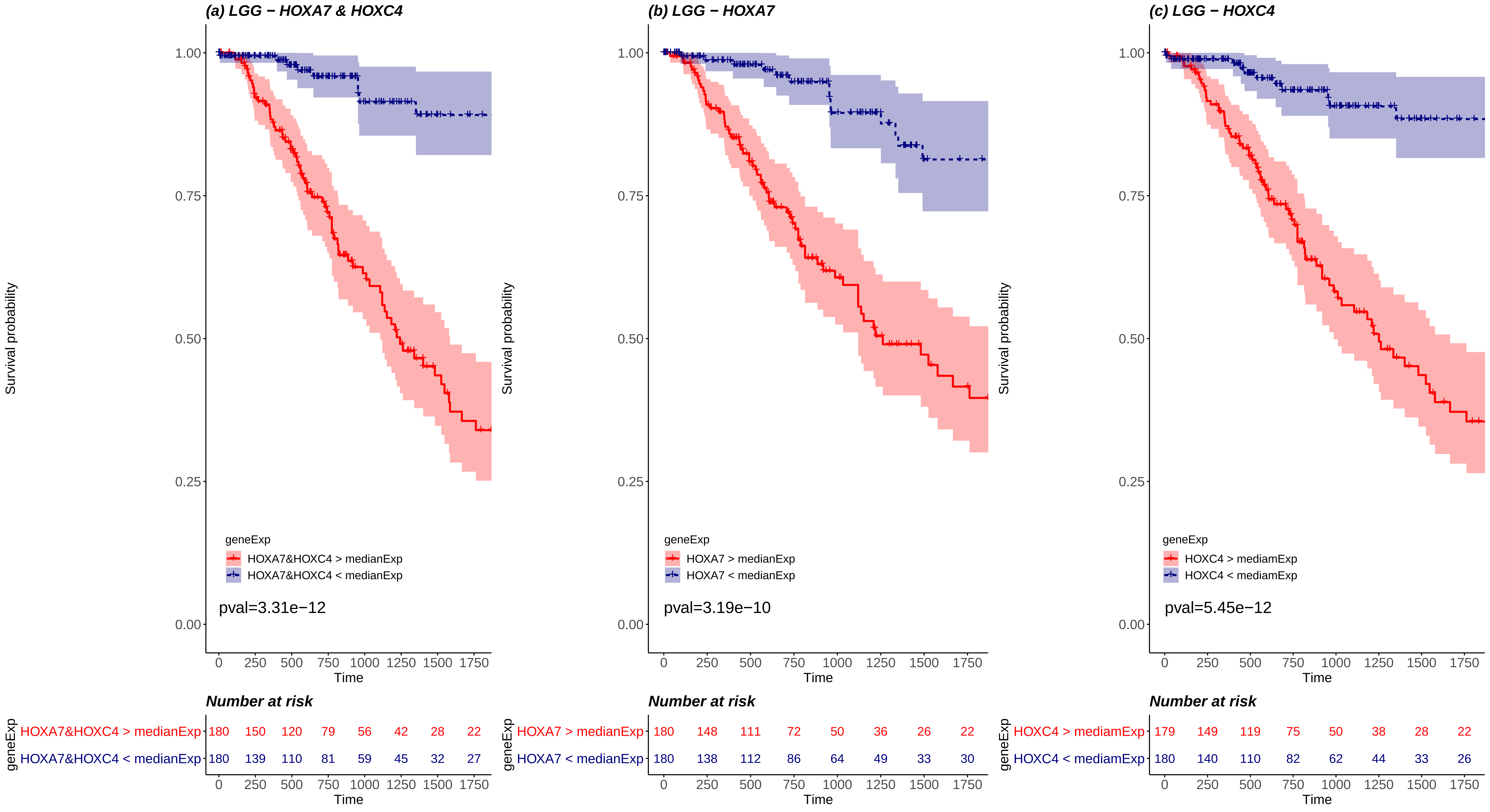

(a) LGG − HOXA7 & HOXC4
(b) LGG − HOXA7
(c) LGG − HOXC4
Survival probability
1.00
0.75
0.50
0.25
0.00
geneExp
HOXA7&HOXC4 > medianExp
HOXA7&HOXC4 < medianExp
pval=3.31e−12
HOXA7 > medianExp
HOXA7 < medianExp
pval=3.19e−10
HOXC4 > mediamExp
HOXC4 < mediamExp
pval=5.45e−12
0 250 500 750 1000 1250 1500 1750
Time
Number at risk
HOXA7&HOXC4 > medianExp 180 150 120 79 56 42 28 22
HOXA7&HOXC4 < medianExp 180 139 110 81 59 45 32 27
HOXA7 > medianExp 180 148 111 72 50 36 26 22
HOXA7 < medianExp 180 138 112 86 64 49 33 30
HOXC4 > mediamExp 179 149 119 75 50 38 28 22
HOXC4 < mediamExp 180 140 110 82 62 44 33 26

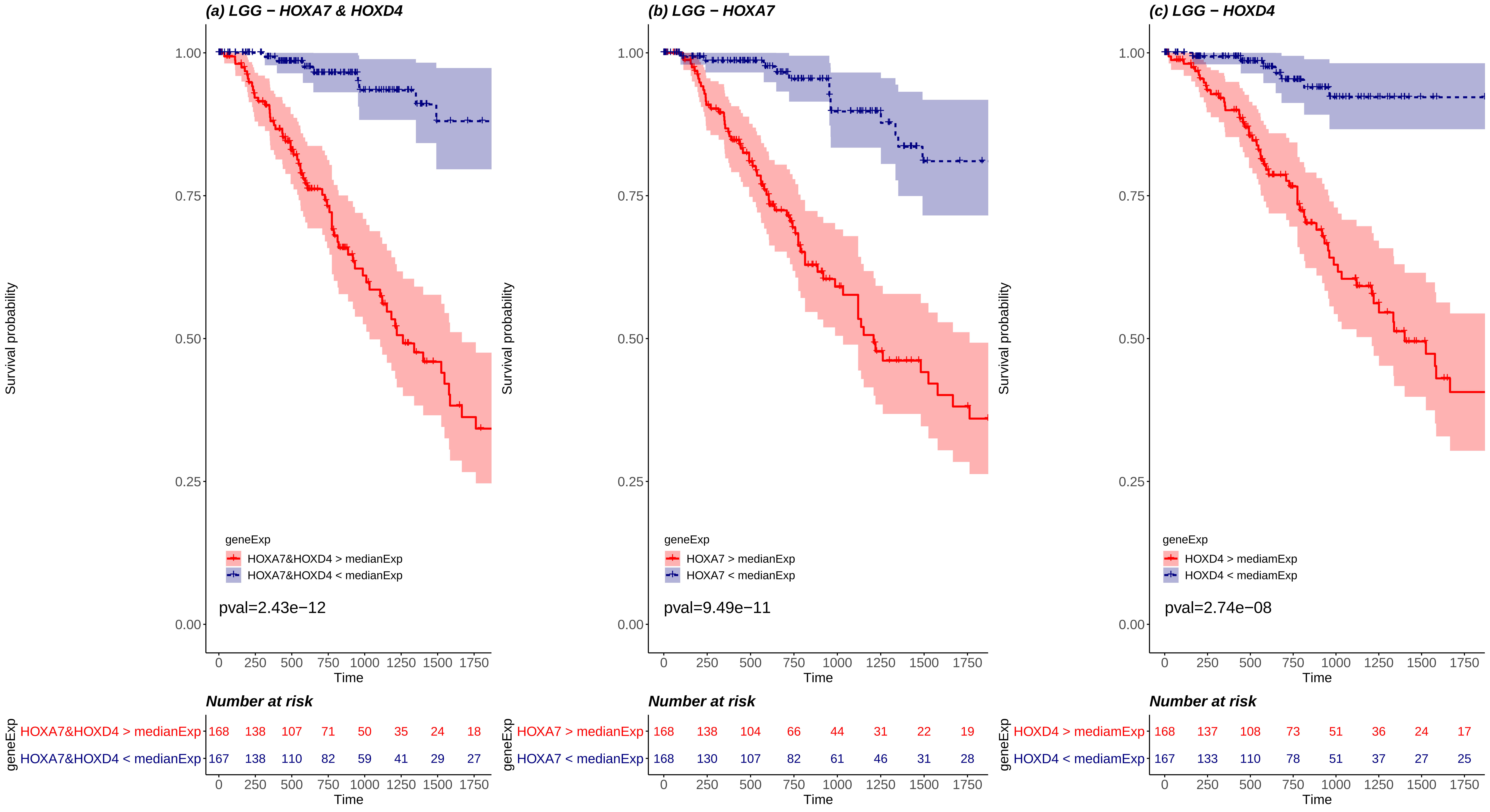
(a) LGG − HOXA7 & HOXD4
(b) LGG − HOXA7
(c) LGG − HOXD4
Survival probability
1.00
0.75
0.50
0.25
0.00
Time
0 250 500 750 1000 1250 1500 1750
geneExp
HOXA7&HOXD4 > medianExp
HOXA7&HOXD4 < medianExp
pval=2.43e−12
HOXA7 > medianExp
HOXA7 < medianExp
pval=9.49e−11
HOXD4 > mediamExp
HOXD4 < mediamExp
pval=2.74e−08
Number at risk
HOXA7&HOXD4 > medianExp 168 138 107 71 50 35 24 18
HOXA7&HOXD4 < medianExp 167 138 110 82 59 41 29 27
HOXA7 > medianExp 168 138 104 66 44 31 22 19
HOXA7 < medianExp 168 130 107 82 61 46 31 28
HOXD4 > mediamExp 168 137 108 73 51 36 24 17
HOXD4 < mediamExp 167 133 110 78 51 37 27 25

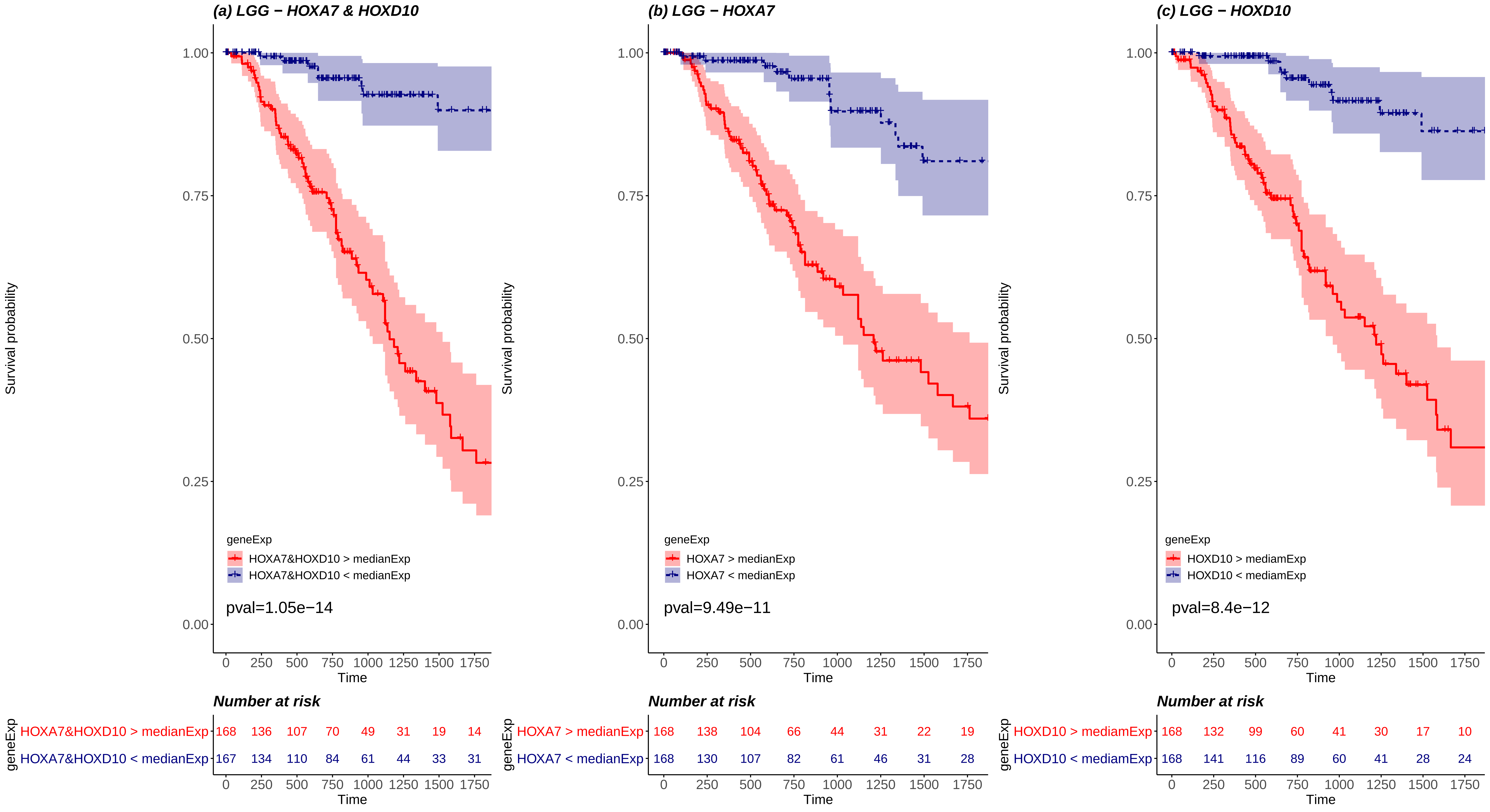
(a) LGG − HOXA7 & HOXD10
(b) LGG − HOXA7
(c) LGG − HOXD10
Survival probability
Time
geneExp
HOXA7&HOXD10 > medianExp
HOXA7&HOXD10 < medianExp
pval=1.05e−14
HOXA7 > medianExp
HOXA7 < medianExp
pval=9.49e−11
HOXD10 > mediamExp
HOXD10 < mediamExp
pval=8.4e−12
Number at risk
HOXA7&HOXD10 > medianExp 168 136 107 70 49 31 19 14
HOXA7&HOXD10 < medianExp 167 134 110 84 61 44 33 31
HOXA7 > medianExp 168 138 104 66 44 31 22 19
HOXA7 < medianExp 168 130 107 82 61 46 31 28
HOXD10 > mediamExp 168 132 99 60 41 30 17 10
HOXD10 < mediamExp 168 141 116 89 60 41 28 24

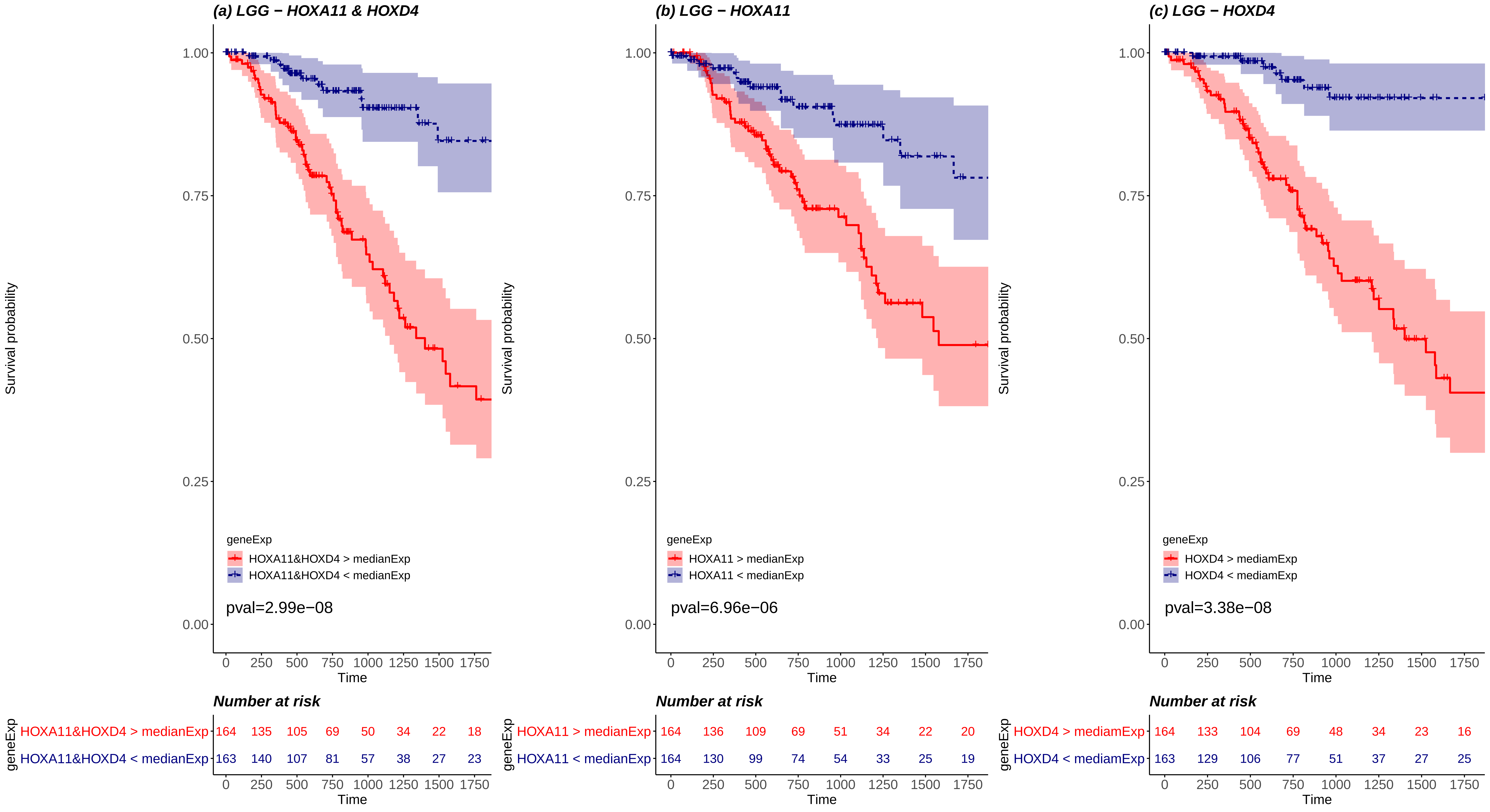
(a) LGG − HOXA11 & HOXD4
(b) LGG − HOXA11
(c) LGG − HOXD4
Survival probability
1.00
0.75
0.50
0.25
0.00
geneExp
HOXA11&HOXD4 > medianExp
HOXA11&HOXD4 < medianExp
pval=2.99e−08
HOXA11 > medianExp
HOXA11 < medianExp
pval=6.96e−06
HOXD4 > mediamExp
HOXD4 < mediamExp
pval=3.38e−08
0 250 500 750 1000 1250 1500 1750
Time
Number at risk
HOXA11&HOXD4 > medianExp 164 135 105 69 50 34 22 18
HOXA11&HOXD4 < medianExp 163 140 107 81 57 38 27 23
HOXA11 > medianExp 164 136 109 69 51 34 22 20
HOXA11 < medianExp 164 130 99 74 54 33 25 19
HOXD4 > mediamExp 164 133 104 69 48 34 23 16
HOXD4 < mediamExp 163 129 106 77 51 37 27 25

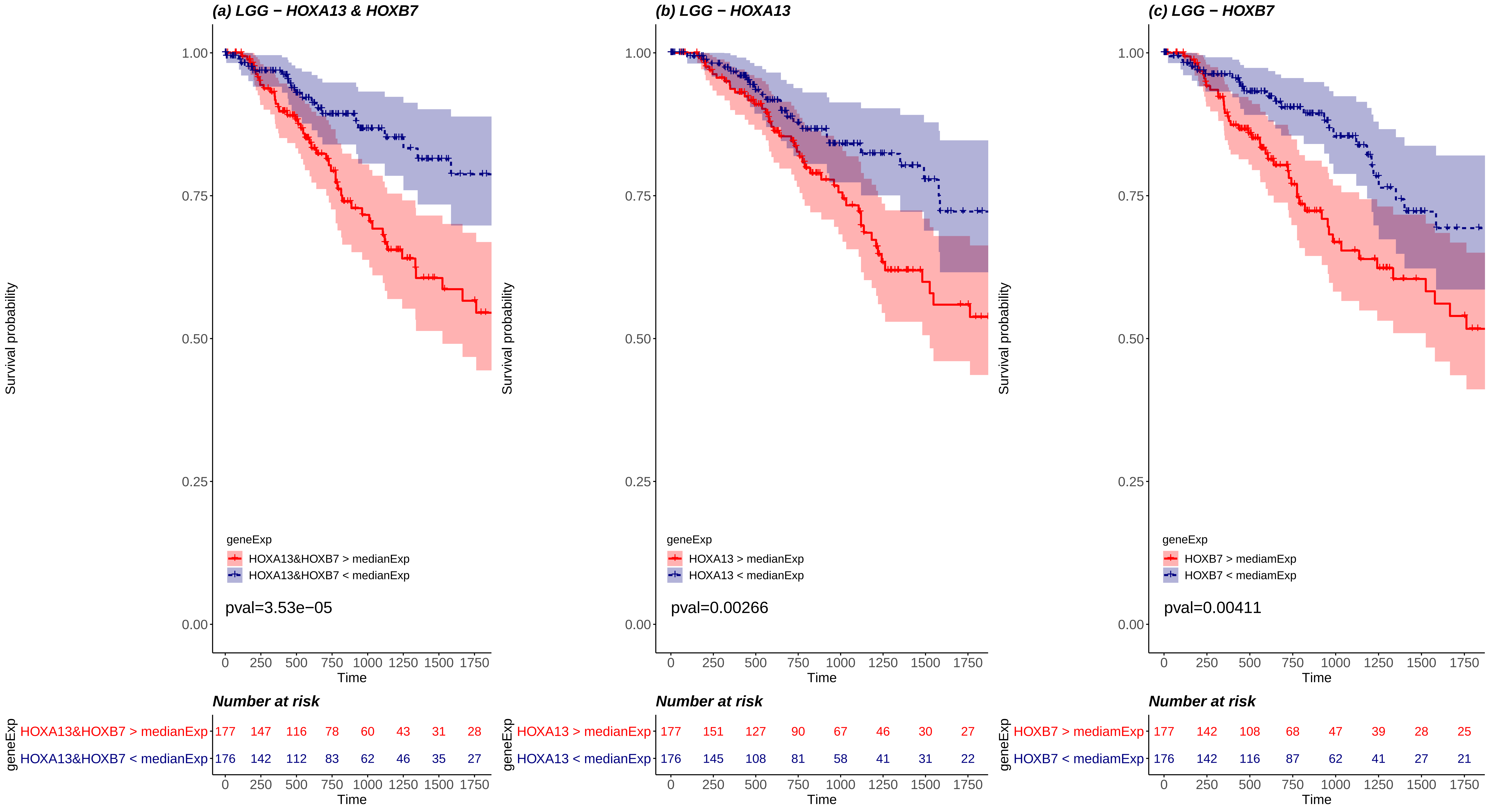
(a) LGG − HOXA13 & HOXB7
(b) LGG − HOXA13
(c) LGG − HOXB7
Survival probability
1.00
0.75
0.50
0.25
0.00
Time
0
250
500
750
1000
1250
1500
1750
geneExp
HOXA13&HOXB7 > medianExp
HOXA13&HOXB7 < medianExp
pval=3.53e−05
HOXA13 > medianExp
HOXA13 < medianExp
pval=0.00266
HOXB7 > mediamExp
HOXB7 < mediamExp
pval=0.00411
Number at risk
HOXA13&HOXB7 > medianExp 177 147 116 78 60 43 31 28
HOXA13&HOXB7 < medianExp 176 142 112 83 62 46 35 27
HOXA13 > medianExp 177 151 127 90 67 46 30 27
HOXA13 < medianExp 176 145 108 81 58 41 31 22
HOXB7 > mediamExp 177 142 108 68 47 39 28 25
HOXB7 < mediamExp 176 142 116 87 62 41 27 21

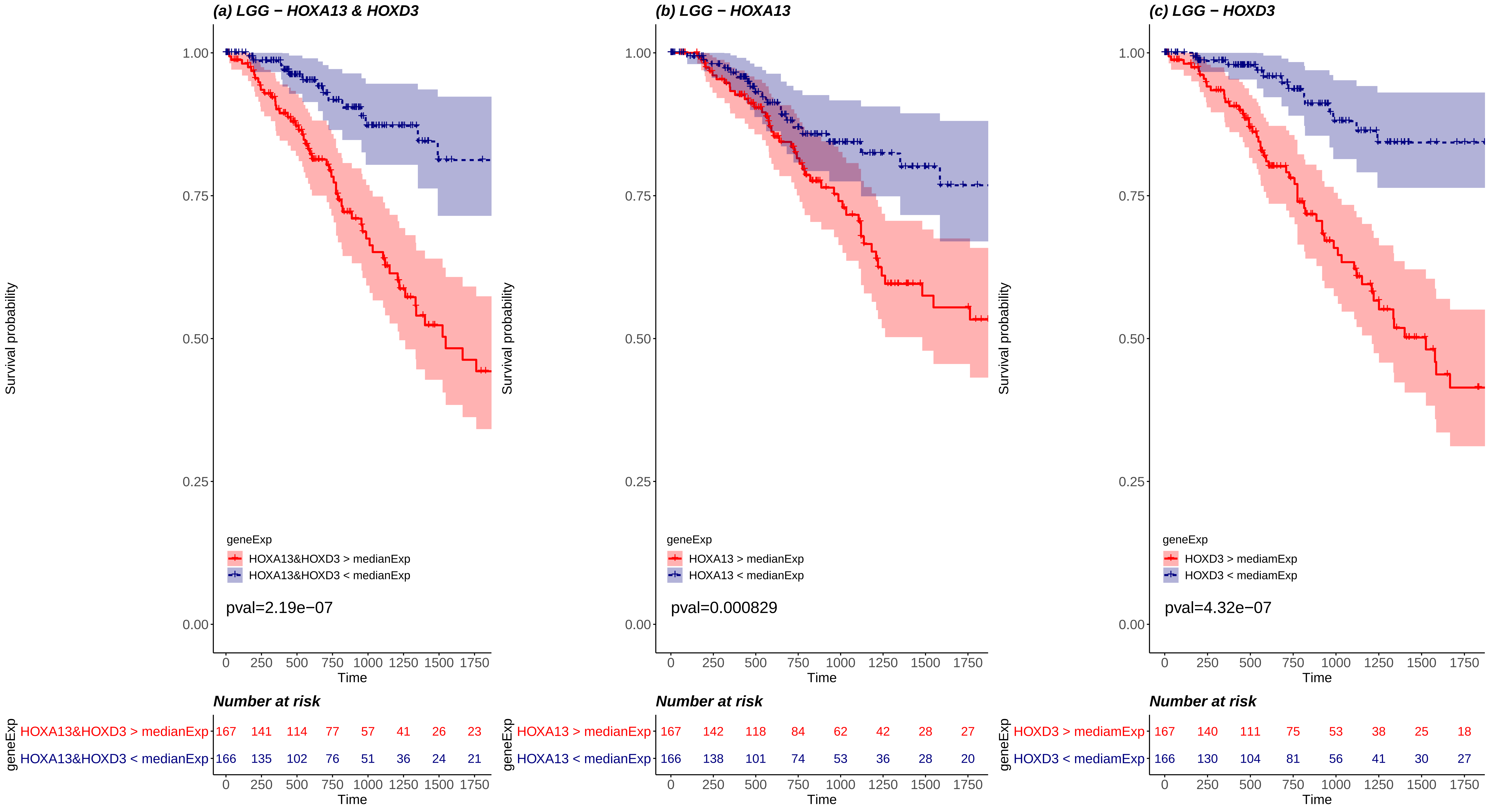

(a) LGG − HOXA13 & HOXD3
(b) LGG − HOXA13
(c) LGG − HOXD3
Survival probability
1.00
0.75
0.50
0.25
0.00
Time
0 250 500 750 1000 1250 1500 1750
geneExp
HOXA13&HOXD3 > medianExp
HOXA13&HOXD3 < medianExp
pval=2.19e−07
HOXA13 > medianExp
HOXA13 < medianExp
pval=0.000829
HOXD3 > mediamExp
HOXD3 < mediamExp
pval=4.32e−07
Number at risk
HOXA13&HOXD3 > medianExp 167 141 114 77 57 41 26 23
HOXA13&HOXD3 < medianExp 166 135 102 76 51 36 24 21
HOXA13 > medianExp 167 142 118 84 62 42 28 27
HOXA13 < medianExp 166 138 101 74 53 36 28 20
HOXD3 > mediamExp 167 140 111 75 53 38 25 18
HOXD3 < mediamExp 166 130 104 81 56 41 30 27

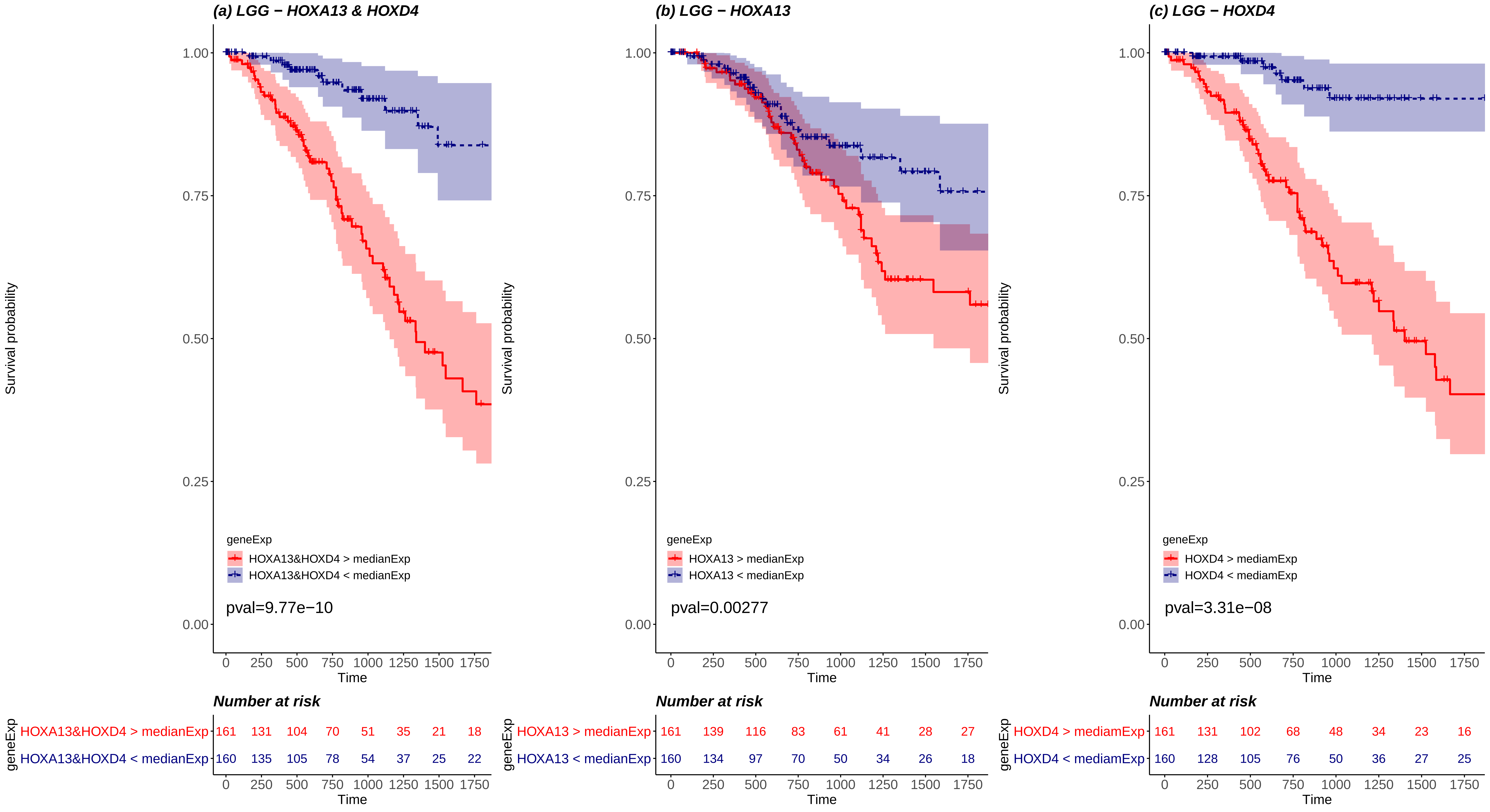

(a) LGG − HOXA13 & HOXD4
(b) LGG − HOXA13
(c) LGG − HOXD4
Survival probability
1.00
0.75
0.50
0.25
0.00
Time
0 250 500 750 1000 1250 1500 1750
geneExp
HOXA13&HOXD4 > medianExp
HOXA13&HOXD4 < medianExp
pval=9.77e−10
HOXA13 > medianExp
HOXA13 < medianExp
pval=0.00277
HOXD4 > mediamExp
HOXD4 < mediamExp
pval=3.31e−08
Number at risk
HOXA13&HOXD4 > medianExp 161 131 104 70 51 35 21 18
HOXA13&HOXD4 < medianExp 160 135 105 78 54 37 25 22
HOXA13 > medianExp 161 139 116 83 61 41 28 27
HOXA13 < medianExp 160 134 97 70 50 34 26 18
HOXD4 > mediamExp 161 131 102 68 48 34 23 16
HOXD4 < mediamExp 160 128 105 76 50 36 27 25

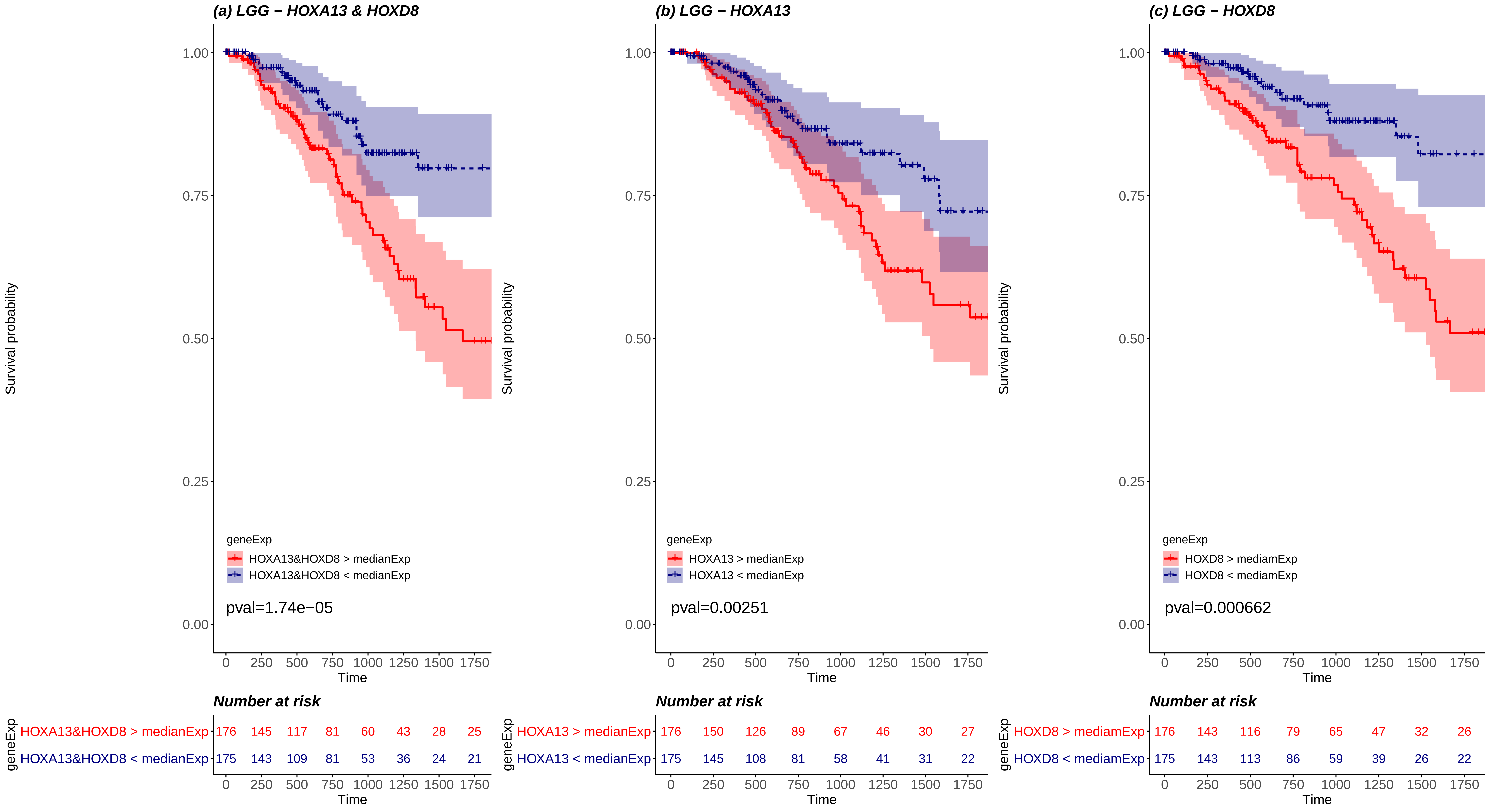
(a) LGG − HOXA13 & HOXD8
(b) LGG − HOXA13
(c) LGG − HOXD8
Survival probability
1.00
0.75
0.50
0.25
0.00
Time
0 250 500 750 1000 1250 1500 1750
geneExp
HOXA13&HOXD8 > medianExp
HOXA13&HOXD8 < medianExp
pval=1.74e−05
HOXA13 > medianExp
HOXA13 < medianExp
pval=0.00251
HOXD8 > mediamExp
HOXD8 < mediamExp
pval=0.000662
Number at risk
HOXA13&HOXD8 > medianExp 176 145 117 81 60 43 28 25
HOXA13&HOXD8 < medianExp 175 143 109 81 53 36 24 21
HOXA13 > medianExp 176 150 126 89 67 46 30 27
HOXA13 < medianExp 175 145 108 81 58 41 31 22
HOXD8 > mediamExp 176 143 116 79 65 47 32 26
HOXD8 < mediamExp 175 143 113 86 59 39 26 22

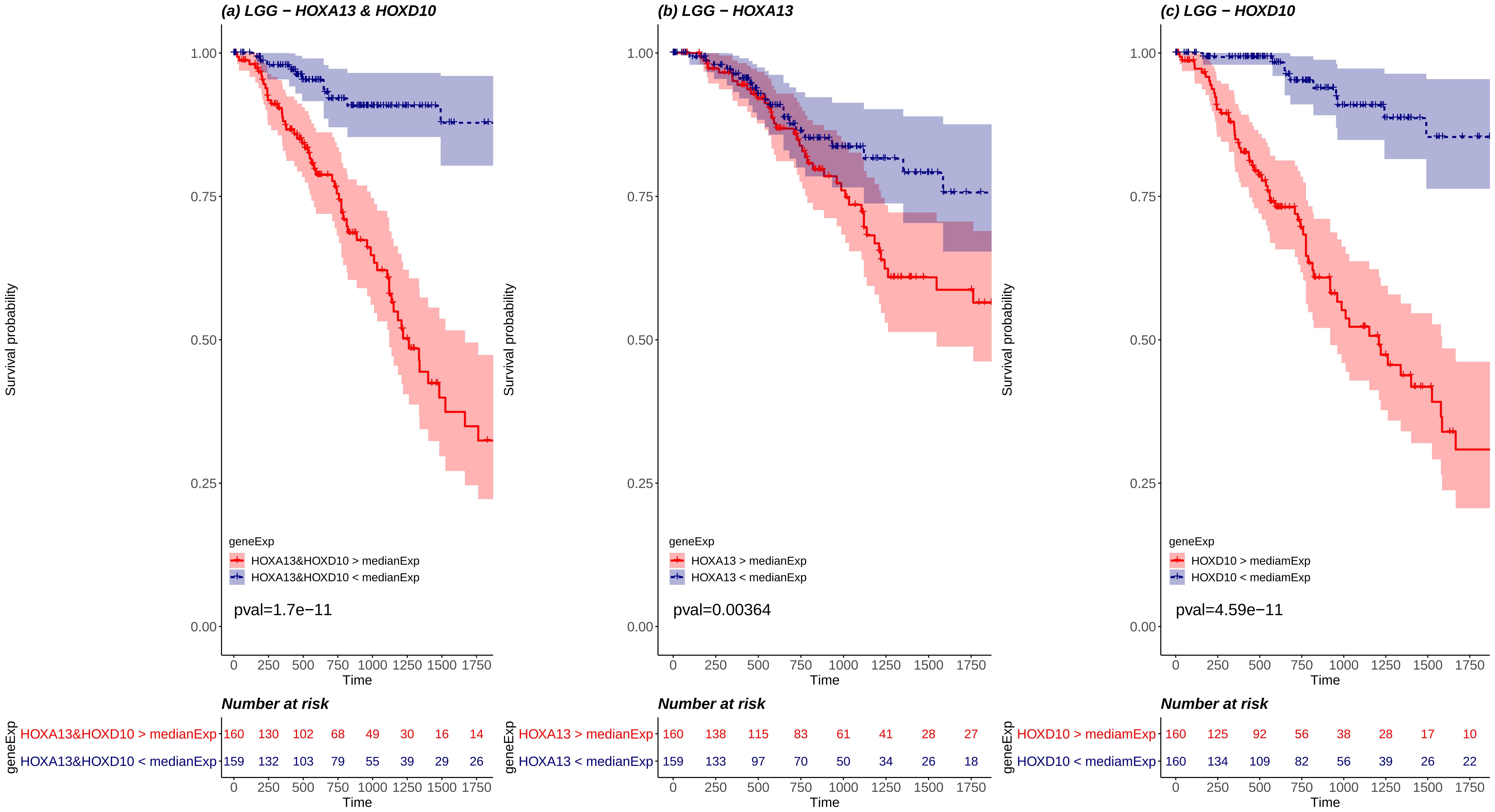

(a) LGG − HOXA13 & HOXD10
(b) LGG − HOXA13
(c) LGG − HOXD10
Survival probability
1.00
0.75
0.50
0.25
0.00
Time
0 250 500 750 1000 1250 1500 1750
geneExp
HOXA13&HOXD10 > medianExp
HOXA13&HOXD10 < medianExp
pval=1.7e−11
HOXA13 > medianExp
HOXA13 < medianExp
pval=0.00364
HOXD10 > mediamExp
HOXD10 < mediamExp
pval=4.59e−11
Number at risk
HOXA13&HOXD10 > medianExp 160 130 102 68 49 30 16 14
HOXA13&HOXD10 < medianExp 159 132 103 79 55 39 29 26
HOXA13 > medianExp 160 138 115 83 61 41 28 27
HOXA13 < medianExp 159 133 97 70 50 34 26 18
HOXD10 > mediamExp 160 125 92 56 38 28 17 10
HOXD10 < mediamExp 160 134 109 82 56 39 26 22

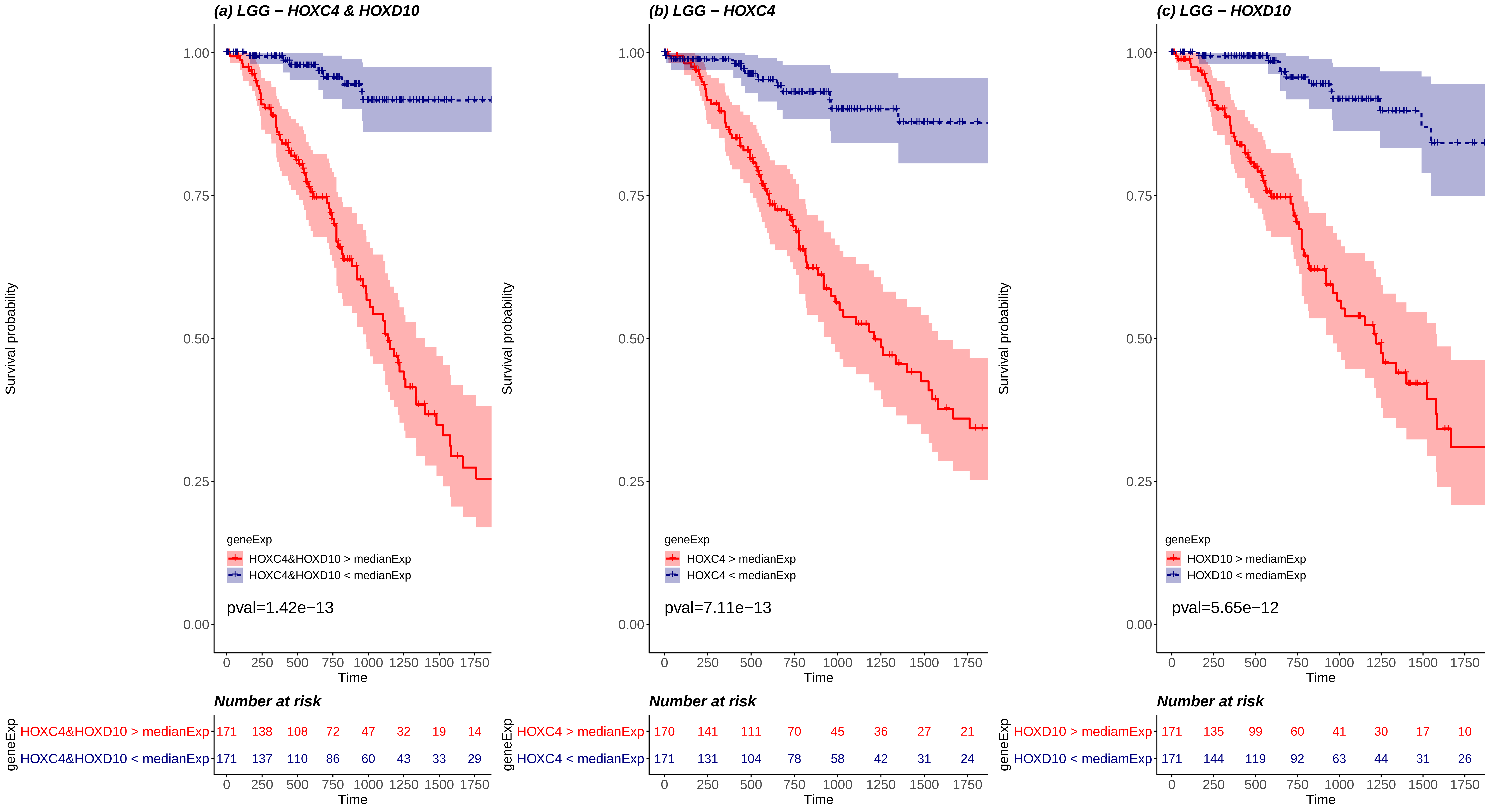
(a) LGG − HOXC4 & HOXD10
(b) LGG − HOXC4
(c) LGG − HOXD10
Survival probability
1.00
0.75
0.50
0.25
0.00
geneExp
HOXC4&HOXD10 > medianExp
HOXC4&HOXD10 < medianExp
pval=1.42e−13
HOXC4 > medianExp
HOXC4 < medianExp
pval=7.11e−13
HOXD10 > mediamExp
HOXD10 < mediamExp
pval=5.65e−12
0 250 500 750 1000 1250 1500 1750
Time
Number at risk
HOXC4&HOXD10 > medianExp 171 138 108 72 47 32 19 14
HOXC4&HOXD10 < medianExp 171 137 110 86 60 43 33 29
HOXC4 > medianExp 170 141 111 70 45 36 27 21
HOXC4 < medianExp 171 131 104 78 58 42 31 24
HOXD10 > mediamExp 171 135 99 60 41 30 17 10
HOXD10 < mediamExp 171 144 119 92 63 44 31 26

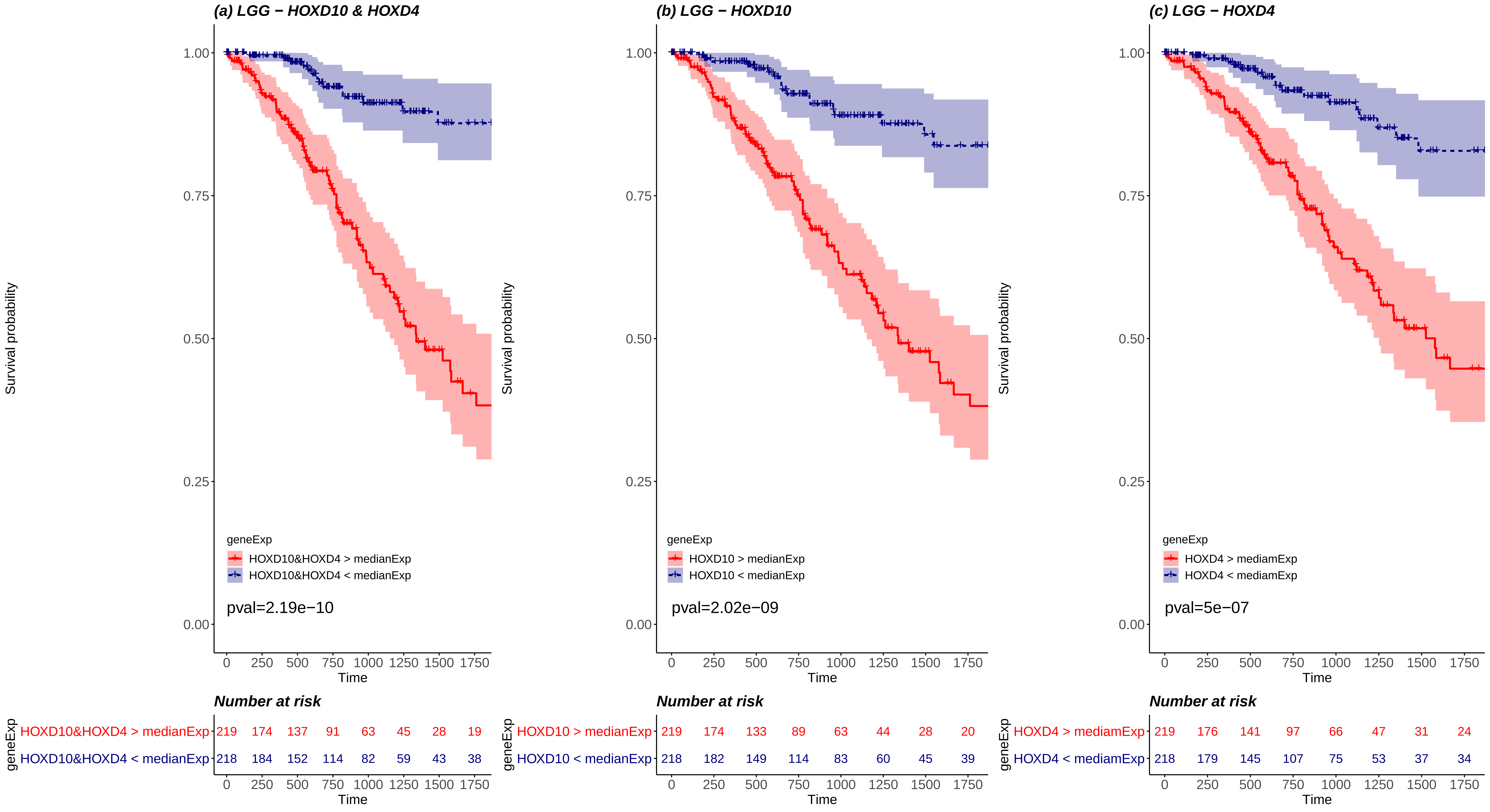
(a) LGG − HOXD10 & HOXD4
(b) LGG − HOXD10
(c) LGG − HOXD4
Survival probability
1.00
0.75
0.50
0.25
0.00
Time
0 250 500 750 1000 1250 1500 1750
geneExp
HOXD10&HOXD4 > medianExp
HOXD10&HOXD4 < medianExp
pval=2.19e−10
HOXD10 > medianExp
HOXD10 < medianExp
pval=2.02e−09
HOXD4 > mediamExp
HOXD4 < mediamExp
pval=5e−07
Number at risk
HOXD10&HOXD4 > medianExp 219 174 137 91 63 45 28 19
HOXD10&HOXD4 < medianExp 218 184 152 114 82 59 43 38
HOXD10 > medianExp 219 174 133 89 63 44 28 20
HOXD10 < medianExp 218 182 149 114 83 60 45 39
HOXD4 > mediamExp 219 176 141 97 66 47 31 24
HOXD4 < mediamExp 218 179 145 107 75 53 37 34
